# Integrative Analysis of Metabolome and Transcriptome Identifies Potential Genes Involved in the Flavonoid Biosynthesis in *Entada phaseoloides* Stem

**DOI:** 10.3389/fpls.2022.792674

**Published:** 2022-05-10

**Authors:** Min Lin, Zhuqing Zhou, Zhinan Mei

**Affiliations:** ^1^School of Pharmaceutical Sciences, South-Central University for Nationalities, Wuhan, China; ^2^Institute of Ethnomedicine, South-Central University for Nationalities, Wuhan, China

**Keywords:** *Entada phaseoloides*, flavonoids, metabolome, transcriptome, structural genes, transcription factors, transport genes

## Abstract

*Entada phaseoloides* stem is known for its high medicinal benefits and *ornamental* value. Flavonoids are one of the *main active constituents* in *E. phaseoloides* stem. However, the regulatory mechanism of flavonoids accumulation in *E. phaseoloides* is lacking. Here, phytochemical compounds and transcripts from stems at different developmental stages in *E. phaseoloides* were investigated by metabolome and transcriptome analysis. The metabolite profiling of the oldest stem was obviously different from young and older stem tissues. A total of 198 flavonoids were detected, and flavones, flavonols, anthocyanins, isoflavones, and flavanones were the main subclasses. The metabolome data showed that the content of acacetin was significantly higher in the young stem and older stem than the oldest stem. Rutin and myricitrin showed significantly higher levels in the oldest stem. A total of 143 MYBs and 143 bHLHs were identified and classified in the RNA-seq data. Meanwhile, 34 flavonoid biosynthesis structural genes were identified. Based on the expression pattern of structural genes involved in flavonoid biosynthesis, it indicated that flavonol, anthocyanin, and proanthocyanin biosynthesis were first active during the development of *E. phaseoloides* stem, and the anthocyanin or proanthocyanin biosynthesis branch was dominant; the flavone biosynthesis branch was active at the late developmental stage of the stem. Through the correlation analysis of transcriptome and metabolome data, the potential candidate genes related to regulating flavonoid synthesis and transport were identified. Among them, the MYBs, bHLH, and TTG1 are coregulated biosynthesis of flavonols and structural genes, bHLH and transporter genes are coregulated biosynthesis of anthocyanins. In addition, the WDR gene TTG1-like (AN11) may regulate dihydrochalcones and flavonol biosynthesis in specific combinations with IIIb bHLH and R2R3-MYB proteins. Furthermore, the transport gene protein TRANSPARENT TESTA 12-like gene is positively regulated the accumulation of rutin, and the homolog of ABC transporter B family member gene is positively correlated with the content of flavone acacetin. This study offered candidate genes involved in flavonoid biosynthesis, information of flavonoid composition and characteristics of flavonoids accumulation, improved our understanding of the MYBs and bHLHs-related regulation networks of flavonoid biosynthesis in *E. phaseoloides* stem, and provided references for the metabolic engineering of flavonoid biosynthesis in *E. phaseoloides* stem.

## Introduction

*Entada phaseoloides* (L.) is a medical and ornamental legume that belongs to the genus *Entada* and grows in the southern of China, other tropical, and sub-tropical regions worldwide. *E. phaseoloides* is notable for its large woody vines (stems) and seed pods. *E. phaseoloides* is a perennial, grows in a hollow of the hills or hillside mixed forest, and usually climbs trees using it as support. The genus *Entada* includes approximately thirty species, belonging to the subfamily Mimosoideae (Ohashi et al., 2010). *Entada* species are rich in saponins and flavonoids (Dong et al., 2012; Roheem et al., 2019; Xiong et al., 2021). The stems of *Entada* species have been widely used as a traditional medicine owing to its pharmacological activities, such as antioxidant activity (Diallo et al., 2001; Njayou et al., 2013; Njayou et al., 2015; Roheem et al., 2019), anti-microbial activity (Teke et al., 2011; Wan et al., 2020), and anti-inflammatory activity (Ayissi et al., 2013; Xiong et al., 2021). The ethyl acetate extracts from the stems of *E. phaseoloides* showed significant antioxidant activity by radical scavenging activity, β-carotene bleaching, reducing power, and superoxide radical scavenging effects *in vitro* experiments, and most of the compounds isolated from ethyl acetate fraction belonged to flavonoids and displayed antioxidant ability by above-mentioned tests (Teke et al., 2011; Zhao et al., 2011; Dong et al., 2012). Thus, flavonoids are considered as one of the important bio-activities in the stems of *E. phaseoloides.* However, the biosynthetic pathway of flavonoids has not been reported in *Entada* species.

Flavonoids are one of the most important polyphenolic compounds, consisting of over 8,000 metabolites widely distributed in plants (Wen et al., 2020). Flavonoids have been reported to have various pharmaceutical activities. They often act as antioxidants due to their effectively scavenging free radical ability (Bors et al., 1990; Grigalius and Petrikaite, 2017), in the rat brain mitochondria *in vitro* experiment showed that flavonoids inhibited the non-enzymatic lipid peroxidation (Ratty and Das, 1998). Flavonoids also exhibited anti-cancer (Kopustinskiene et al., 2020), anti-inflammation (Mower et al., 1984), antimicrobial activity (Cushnie and Lamb, 2005), neuroprotective activities (Spagnuolo et al., 2018), and antidiabetic effects (Sarkhail et al., 2007; Bansal et al., 2012; Yang et al., 2015) in many *in vitro* and animal models. In addition, flavonoids also possess multiple ecological roles, including regulation of plant–microbe and plant–plant interactions (Leoni et al., 2021), facilitating pollination (Saigo et al., 2020), improving resistance to biotic and abiotic stresses (Casas et al., 2016; Barcelo et al., 2017; Tohge et al., 2018) and UV-light protection (Tohge et al., 2016; Peng et al., 2017). Thus, flavonoids are widely used for the pharmaceutical, cosmetic, and food industries and have gained increasing attention (Soto-Vaca et al., 2012; Grigalius and Petrikaite, 2017; Khan et al., 2021).

Flavonoids are characterized by a common diphenylpropane (C6-C3-C6) backbone in which two aromatic rings are connected by a three-carbon chain (Stobiecki and Kachlicki, 2006; Wen et al., 2020). Based on the oxidation pattern of the heterocyclic C ring, flavonoids are divided into several categories, including flavones, flavanones, flavonols, flavanols, isoflavones, and anthocyanidins (Wen et al., 2020). The flavonoid synthesis pathway begins with the catalytic action of phenylalanine ammonia-lyase (PAL) on the precursor phenylalanine and then *trans*-cinnamate 4-monooxygenase (C4H), leading to the production of chalcone (Koukol and Conn, 1961; Russell and Conn, 1967; Ververidis et al., 2007; Jia et al., 2008; Hassani et al., 2020). The first committed step of flavonoid synthesis is mediated through chalcone synthase (CHS), which catalyzes malonyl-CoA and *p*-coumaroyl-CoA to generate naringenin chalcone (Kreuzaler and Hahlbrock, 1975; Kreuzaler et al., 1983). Then, naringenin chalcone undergoes isomerization to produce flavanone naringenin through chalcone isomerase (CHI) (Moustafa and Wong, 1967; Jez et al., 2000). Next, the flavanones act as substrates for a series of other enzymes, giving rise to different subclasses of flavonoids, such as flavanones, dihydroflavonols, and anthocyanins; these enzymes include flavonoid 3-hydroxylase (F3H), flavonoid 3′-hydroxylase (F3′H), flavonoid 3′5′-hydroxylase (F3′5′H), flavanone 3b-hydroxylase (FHT), flavonol synthase (FLS), dihydroflavonol 4-reductase (DFR), anthocyanidin synthase (ANS), and anthocyanin reductase (ANR) (Holton and Cornish, 1995; Winkel, 2006; Gebhardt et al., 2007; Tohge et al., 2017; Jun et al., 2018). Heterologously overexpression of *Ginkgo biloba* L. *GbF3*′*5*′*H1* can increase the contents of 4′,5-dihydroxy-7-glucosyloxyflavanon, epicatechin, and gallocatechin in transgenic *Populus* (Wu et al., 2020). High expression of the *CHS* gene enhances the accumulation of flavonoids in the leaves of *Perilla frutescens* based on the metabolomic and transcriptomic data (Jiang et al., 2020). Moreover, some transcription factors (TFs) have been proved to participate in regulating flavonoid synthesis. Overexpression of *Poplar MYB117* promotes anthocyanin synthesis in all tissues and enhances flavonoid B-ring hydroxylation by upregulating the *F3*′*5*′*H* gene (Ma et al., 2021). Overexpressed *FtMYB31* increases the rutin content of *Fagopyrum tataricum* Gaertn (Hou et al., 2021). Wang et al. (2018) prove that overexpression of *MdWRKY11* could promote the expression of *F3H*, *FLS*, *DFR*, *ANS*, and *UFGT*, thereby increasing the accumulation of flavonoids and anthocyanin in apple calli. In *Petunia hybrida*, an R2R3-MYB TF *AN4* promotes anthocyanin biosynthesis by enhancing the expression of anthocyanin biosynthesis genes, such as *CHS*, *CHI*, *F3H*, and *DFR* (Zhang et al., 2021). *Cucumis sativus CsMYB60* also can promote flavonol and proanthocyanidin biosynthesis by inducing the expression of *CsFLS* and *CsLAR* (Li et al., 2020). Among the TFs, R2R3-MYB, WD-repeat proteins (WDR), and basic helix-loop-helix proteins (bHLH) have been received more attention (Koes et al., 2005; Mehrtens et al., 2005; Jaakola, 2013; Xu et al., 2015; Karppinen et al., 2021).

In this study, untargeted metabolite analysis was performed to determine global metabolic profiles and analyze the characteristic of flavonoid accumulation in three developmental stages of stems from *E. phaseoloides*. Global gene expression profiling was explored during the development of the stem, and the structural genes of flavonoid biosynthesis and some TFs were identified and classified by transcriptome data. Meanwhile, the coexpression networks and candidate genes regulating the biosynthesis of flavonoids in *E. phaseoloides* were revealed by an integrated analysis of metabolome and transcriptome. This study not only clarified the accumulation of flavonoids in *E. phaseoloides* stem at different development stages but improved the current understanding of the MYBs and bHLHs-related molecular network of flavonoid synthesis in *E. phaseoloides* and provided gene sequences information of flavonoid biosynthesis and research clues for the future studies in other medicinal plants and legume plants.

## Materials and Methods

### Plant Materials

*Entada phaseoloides* stem tissue used for this study was obtained from the mixed forest of Botanical Garden of Xishuangbanna South Medicine at an altitude of about 553 m, Xishuangbanna, Yunnan Province, China. The *Mucuna sempervirens* Hemsl is next to *E. Phaseoloides.* The stems were collected at 15, 45, and 75 days at fruit developmental stages, corresponding to the stem_young (S1), stem_older (S2), and stem_oldest (S3), respectively. The temperature on the day of harvest was 27°C. Samples were collected on ice and frozen immediately in liquid nitrogen and stored at −80°C until further use. Additionally, all samples were divided into two parts for metabolites determination and extraction of RNA for transcriptome sequencing. To ensure the consistency of subsequent correlation analysis, the metabolites and transcripts were assessed from the same samples.

### Extraction and Separation of Metabolites

Each sample had six independent biological replicates. A total of 18 samples were collected as metabolite studies. Metabolite extraction for non-targeted metabolite profiling was conducted as previously described (Tohge and Fernie, 2010). The tissues (25 mg) were weighed and put into a 1.5-ml EP tube. A total of 800 μl pre-cooled precipitant (methanol: acetonitrile: water = 2:2:1) were added into the tube. The tissues were crushed with steel beads using a grinder for 4 min at 60 Hz. After ultrasonic extraction for 10 min (power 80 HZ) in an ice bath, the mixture was frozen for 120 min and centrifugated at 25,000 *g* for 15 min at 4°C. The supernatants were collected and repeated one time and then dried by a freeze dryer. Redissovle by adding 600 μl of 10% methanol solution, sonicate for 10 min (power 80 Hz) in an ice bath, and centrifuge for 15 min at 4°C to take the supernatant; about 50 μl of each sample was taken and mixed into a QC sample.

High-resolution mass spectrometry (MS) was performed on a Xevo G2-XS QTOF mass spectrometer (Waters, United Kingdom) following chromatographic separation on a Waters 2777C ultra-performance liquid chromatography (UPLC) system (Waters, United Kingdom) using a Waters ACQUITY UPLC HSS T3 column (100 mm * 2.1 mm, 1.8 μm, Waters, United Kingdom), with column temperature control at 50°C. The elution gradient at 0.4 ml/min using solvent A (water + 0.1% formic acid) and solvent B (acetonitrile + 0.1% formic acid) was applied/set as follows: 0–2 min with 100% phase (A), followed by a 2–11 min gradient from 0 to 100% (B), 11–13 min with 100% (B), 13–15 min gradient from 0 to 100% (A), the injection volume for each sample was 5 μl. The QTOF mass spectrometer was operated in both positive and negative ion modes. The MS data were acquired in Centroid MSE mode. The TOF mass range was from 50 to 1,200 Da, and the scan time was 0.2 s. For the MS/MS detection, all the precursors were fragmented using 20–40 eV, and the scan time was 0.2 s.

### Pre-identification and Analysis of Metabolites

Mass spectrometry raw data were preprocessed and analyze using Progenesis QI (v2.2, Waters, Milford, MA, United States) and metaX (Wen et al., 2017). The ions meeting a relative standard deviation (RSD) ≤ 30% threshold were selected for downstream statistical analysis. Metabolites were identified by a comparison of the measured molecular mass-to-charge ratio (m/z) or molecular weight with information available in the KEGG database. The information of adducts and isotopes is utilized to assist in metabolite identification if it is present. The mass deviation was 10 ppm. The pre-identifications are Level II following the standardization of metabolomics data after the International Metabolomics Society. Rutin, myricitrin, and acacetin were verified based on the authentic standard. The related information (MS/MS spectrum) was added to Supplementary File 1.

The variable importance in projection (VIP) values of the first two principal components in the multivariate PLS-DA model, combined with fold-change (FC) and *q*-value of univariate analysis, were used to choose differential metabolites. The filtering rules are VIP ≥ 1; fold-change ≥ 1.2 or ≤0.8333; and *q*-value < 0.05. Metabolic pathway analysis is based on the KEGG database.

### Transcriptome Sequencing (RNA-Seq) and Analysis

The stem samples of three developmental stages used for RNA-seq were the same as metabolome analysis samples. Each sample had three independent biological replicates. The total RNA and mRNA from *E. phaseoloides* stems were obtained as previously described (Fu et al., 2021). The RNA-seq libraries were constructed according to Illumina instruction and then sequenced using an Illumina HiSeq X Ten platform. Low-quality raw read sequences were removed using SOAPnuke with default parameters (-l 15 -q 0.2 -n 0.05) (v1.5.2^1^). The clean reads were mapped to *E. phaseoloides* reference genome sequence using HISAT with default parameters (--phred64 --sensitive --no-discordant --no-mixed -I 1 -X 1000) (v2-2.0.4^2^) and then assembled by StringTie with default parameters (-f 0.3 -j 3 -c 5 -g 100 -s 10000 -p 8) (v1.3.3b^3^). The fragments per kilobase per million mapped fragments (FPKM) values and transcripts per million (TPM) values were then calculated for each gene in all samples. Differentially expressed genes (DEGs) were identified by DESeq2 (v1.34.0, fold-change ≥ 2 and adjusted *p*-value ≤ 0.05) (Anders and Huber, 2010; Love et al., 2014) and annotated to Nr, GO, and KEGG database.

### Identification of Enzyme-Coding Genes and Transcription Factors

To identify the candidate structure genes involved in the biosynthesis of flavonoids, the genes that are annotated to these pathways (ko00940, ko00941, ko00942, ko00943, and ko00944) using KEGG (*E*-value ≤ 1e--10) were retrieved. Subsequently, the identified candidate genes involved in flavonoid biosynthesis were confirmed using the knowledge-based identification of pathway enzymes (KIPEs^4^) (Pucker et al., 2020a).

The identification and classification of MYB and bHLH were based on the signature domain and phylogenetic tree as described in the previous studies (Pucker, 2021; Song et al., 2021). The MYB domain sequences of published multiple plant species were merged to generate a bait sequence collection. MYB candidates are checked for conserved domains and assigned to orthologs in other plant species (Pucker, 2021).

Protein sequences of *Arabidopsis* from the TAIR database were established as references. The pipeline for automatic identification and annotation in a bait sequence data set was used to get *E. phaseoloides* MYB candidates. Non-redundant MYB genes were obtained by mapping identified gene models of the previous step to unique loci in the genome. For the bHLH, the most conserved sequence in the bHLH region (Toledo-Ortiz et al., 2003) was used for tBLASTn searches. Protein sequences of *Arabidopsis* from the TAIR database were established as references. The intact bHLH domain was analyzed by the HMMER (v3.0). Other steps were similar to MYB processing. Multiple sequence alignments of the predicted genes and *Arabidopsis* were performed using muscle (v3.8.31) with default parameters. Phylogenetic trees were constructed by FastTree (v2.1) using the maximum likelihood (ML) method. *E. phaseoloides* genes homologs were classified according to their relationships with corresponding *Arabidopsis*. The resulting genes were named following the position of the chromosome.

### Integrated Analysis Between Metabolite Profiling and Transcriptome

To identify the potential key genes and networks involved in flavonoid biosynthesis in *E. phaseoloides*, correlation analysis was performed by calculating the Pearson correlation coefficient (PCC) values in the R software between each set of variables (DEGs and differentially accumulated metabolites) across the profiles. The | PCC| ≥ 0.80 and *p*-value ≤ 0.05 between DEGs with metabolites were selected as significant correlations. The correlation network was visualized by Cytoscape software (v 3.6.1). The sequences of genes involved in the network are presented in the Supplementary Files 2, 3.

## Results

### Metabolite Profiling of Stem Samples

**FIGURE 1:**
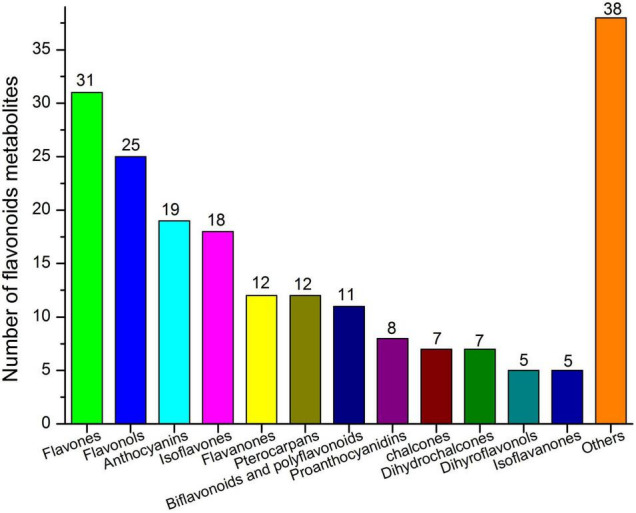
Classification and statistical analysis of all detected flavonoids in all samples. The *x*-axis represents the different categories, and the *y*-axis represents the number of each category.

Untargeted metabolite analysis was performed to determine the global metabolic profiles of *E. phaseoloides* stem tissues. Among all the detected phytochemical compounds, terpenoids, flavonoids, and alkaloids are the main metabolites (Supplementary Figure 1). Partial least squares discriminant analysis (PLS-DA) was conducted to obtain an overview of metabolic changes and plot comparison of differences and similarities. The PLS-DA of the metabolites showed a clear separation in the first principal component for the composition of stems metabolites in both positive and negative modes, explaining 32.06 and 35.63% of the variation in the data, respectively; the metabolite profiling of stem_oldest (S3) was obviously different from stem_young (S1) and stem_older (S2) tissues (Supplementary Figure 2). Flavonoids are one of the main important active ingredients in *E. phaseoloides* stems (Teke et al., 2011; Dong et al., 2012). A total of 198 putative flavonoids were detected, and flavones (31), flavonols (25), anthocyanins (19), isoflavones (18), flavanones (12), pterocarpans (12), biflavonoids, and polyflavonoids (11), chalcones (7), proanthocyanidins (8), and dihydrochalcones (7) were the top 10 most abundant classes (Figure 1 and Supplementary Table 1). This is the first time that so many types of flavonoid compounds were isolated in *E. phaseoloides*, including myricitrin and acacetin. Combing VIP in the PLS-DA model variable, FC, and q-value, 366 differentially accumulated metabolites (DAMs) were identified among all the compared samples. KEGG pathway enrichment analysis indicated that these DAMs mainly enriched in pathways of flavonoid biosynthesis, flavone and flavonol biosynthesis, anthocyanin biosynthesis, and isoflavonoid biosynthesis (Supplementary Figure 3). Taken together, the flavonoids were analyzed in detail in this study.

### Flavonoids Differences Among Different Stem Samples

**FIGURE 2:**
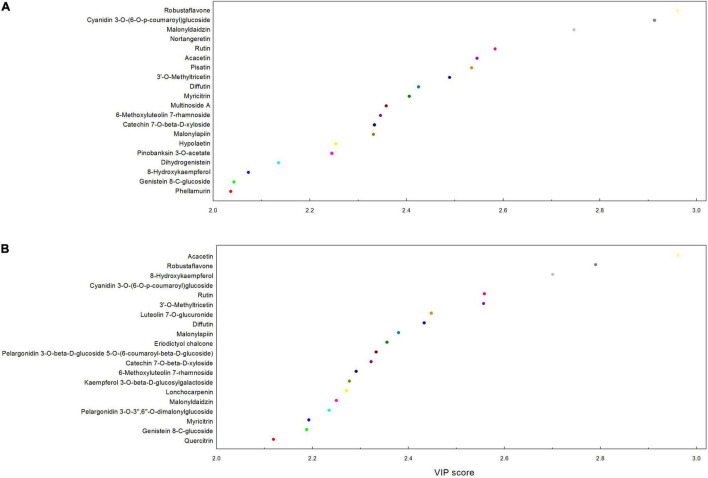
Variable importance in projection (VIP) score of the top 20 flavonoids in each compared group. Results were from the PLS-DA. **(A)** S2 versus S3 and **(B)** S1 versus S3. The VIP scores displayed the relative contribution of these flavonoids to the separation among different stems. The higher the VIP score, the larger the contributions. Stem_young, stem_older, and stem_oldest were renamed as S1, S2, and S3, respectively.

A total of 97 differentially accumulated flavonoids (DAFs) were detected among all comparison groups. Flavones (17), flavonols (17), and anthocyanins (10) are the three major categories in DAFs (Supplementary Table 2). In detail, there were 0, 72, and 84 significantly DAFs, respectively, in the 3 comparison groups, including S1 versus S2, S2 versus S3, and S1 versus S3; there were 61 DAFs changed in common between S2 versus S3 and S1 versus S3. Pairwise comparison of three groups revealed that the difference of content of flavonoids between the S1 and S2 was small, whereas large between S2 versus S3 and S1 versus S3 (Supplementary Table 2). Based on the VIP values of the PLS-DA, the 20 dominant components responsible for the separation among different stems in DAFs were identified and shown in Figure 2. In the comparison between S2 and S3, these 20 flavonoids included five flavones, three flavonols, two isoflavones, two dihyroflavonols, one anthocyanin, one biflavonoid, and polyflavonoid, one flavan 3-ol, one flavan, one flavonoid glycoside, one isoflavan, one o-methylated flavonoid, and one pterocarpan (Figure 2A and Supplementary Table 3); whereas for the S1 and S3 comparison groups, there were five flavonols, four flavones, three anthocyanins, two isoflavones, one chalcone, one 3-arylcoumarin, one biflavonoid and polyflavonoid, one flavan 3-ol, one flavan, and one O-methylated flavonoid (Figure 2B and Supplementary Table 3). Among them, rutin, myricitrin, 6-methoxyluteolin 7-rhamnoside, 3-methoxyapigenin, cinnamtannin A1, and (-)-epiafzelechin showed significantly higher levels in S3 than both S1 and S2, whereas the content of acacetin, pinobanksin 3-O-acetate, 8-hydroxykaempferol, phellamurin, quercitrin, pelargonidin 3-O-(6-caffeoyl-beta-D-glucoside), desmethylxanthohumol, dasytrichone, delphinidin 3-(6-*p*-coumaroyl) glucoside, and eriodictyol chalcone was significantly lower in S3 then both S1 and S2 (Supplementary Table 3).

### Transcriptome Analysis of Stem Samples

To gain the global gene expression in three different types of stem samples, a total of 57.85 Gb of clean data were obtained, and the clean reads ranged from 42.07 to 43.44 Mb in each library, with an average of 96.85% of bases scoring Q30. All the clean reads were mapped to the reference genome sequence with mapping ratios in the range of 86.23–91.81% (Supplementary Table 4). These data demonstrated that the transcriptome sequencing was high quality and could be used for further analysis. Totally, 24,240 genes were detected for further analysis, and their gene expression levels were estimated by FPKM and TPM values. Among these genes, 21,462 were expressed in all three different stems (S1, S2, and S3), and 316, 456, and 581 specific expressed genes were found in S1, S2, and S3, respectively (Supplementary Figure 4).

**FIGURE 3:**
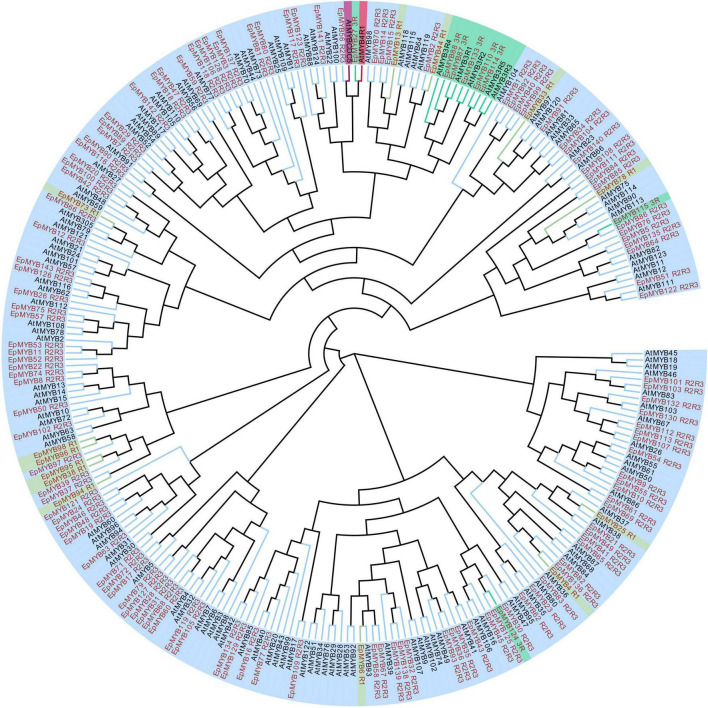
Phylogenetic tree of *EpMYBs*. The *EpMYBs* are marked in red, *AtMYBs* are marked in black. The subgroups are marked in different colors on the periphery of the circle.

**FIGURE 4:**
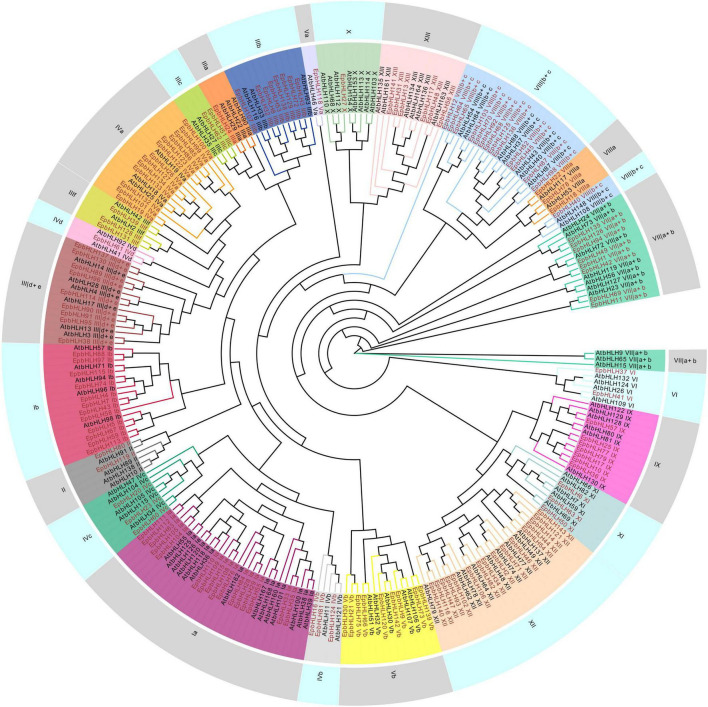
Phylogenetic tree of *EpbHLHs*. The *EpbHLHs* are marked in red, *AtbHLHs* are marked in black. The subgroups are marked in different colors on the periphery of the circle.

A total of 143 MYBs and143 bHLHs were identified in *E. phaseoloides* (Supplementary Table 5). To unravel the evolutionary relationship of these genes, the phylogenetic trees were constructed. The *Arabidopsis* MYB family (131 members, including 124 R2R3-MYBs, five MYB3R, one CDC5, and one MYB4R) and bHLH family (116 members) were used, respectively. A set of 120 R2R3-MYBs proteins, 13 R1-MYB proteins, 8 R1R2R3-MYB (MYB3R) proteins, and 2 unclassified MYB proteins (pseudo) were identified (Supplementary Table 5 and Figure 3). R2R3-MYBs account for the largest subfamily size in *E. phaseoloides. E. phaseoloides* bHLHs were classified into 23 subfamilies, including subgroup Ia, XII, IVa, Ib, VIIIb+c, IIId+e, Vb, III b, VIIa+b, IX, XIII, IIIc, IVb, IVc, VIIIa, XI, II, IIIf, VI, IIIa, IVd, Va, and X based on *Arabidopsis* bHLH subgroups. Among them, Ia (16 members), XII (16 members), IVa (13 members), Ib (12 members), and VIIIb+c (14 members) were the largest top five subgroups of *EpbHLHs*, while subgroups IIIa, IVd, Va, and X were the smallest, each with only one member (Supplementary Table 5 and Figure 4). The number of subgroup XII was similar to *Arabidopsis* and *Ficus carica* L., but the number of subgroup X was significantly less than these two species.

### Differential Gene Expression Analysis

**FIGURE 5:**
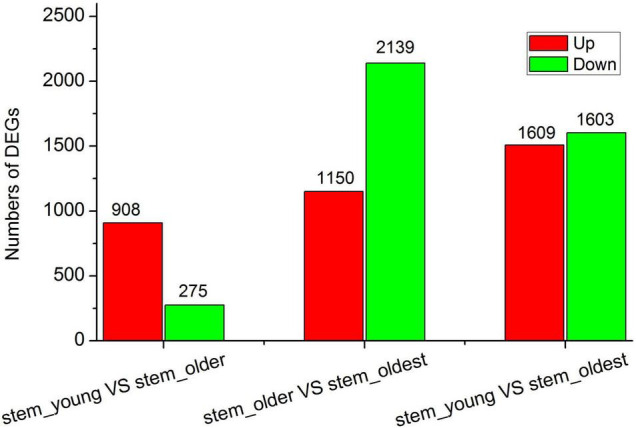
Numbers of DEGs among all comparison group.

Based on the fold-change of gene expression and a false discovery rate (FDR), a total of 1,183 (908 up- and 275 downregulated), 3,289 (1,150 up- and 2,139 downregulated), and 3,112 (1,509 up- and 1,603 downregulated) DEGs were found in the S1 versus S2, S2 versus S3, and S1 versus S3, respectively (Figure 5 and Supplementary Table 6). The results demonstrated that the expression profile of S1 and S2 is closer, whereas the gene expressions between S2 and S3, S1, and S3 were obviously different, which is similar to the results obtained based on the metabolome analysis. This implied that the DAMs in these samples have certain correlations with regulation by DEGs.

To explore the potential biological significance of these DEGs, GO, and KEGG function annotation and enrichment analysis were performed. GO functional classification analysis showed that all the DEGs could be grouped into 48 functional groups, including 23 biological process, 15 cellular component, and 10 molecular function categories (Supplementary Table 7). Within the biological processes, cellular process, metabolic process, and biological regulation were the most enriched terms, in the cellular component category, the top three represented terms were membrane, membrane part, and cell, and under the molecular function category, the most abundant terms were catalytic activity, binding, and transporter activity. To further illustrate the alterations of metabolic pathways during stem development, the KEGG analysis of all the DEGs was conducted. A total of 976 (S1 versus S2), 2,665 (S2 versus S3), and 2,554 (S1 versus S3) DEGs were grouped to 117, 130, and 133 KEGG pathways, respectively, and five pathways that are related to the biosynthesis of flavonoids were enriched in all comparison groups, including phenylpropanoid biosynthesis, flavonoid biosynthesis, flavone and flavonol biosynthesis, anthocyanin biosynthesis, and isoflavonoid biosynthesis (Supplementary Table 8). KEGG significantly enrichment analysis showed a marked difference. Starch and sucrose metabolism and flavonoid biosynthesis were significantly enriched in the S1 versus S2 comparison group, and the number of upregulated DEGs were significantly more than downregulated DEGs in these pathways. Starch and sucrose metabolism, plant hormone signal transduction, phenylpropanoid biosynthesis, other glycan degradation, and MAPK signaling pathway were significantly enriched in the comparison group of S2 versus S3. Other glycan degradation, flavone and flavonol biosynthesis, and MAPK signaling pathway were significantly enriched in the S1 versus S3 comparison group (Supplementary Table 8). The genes involved in these significantly enriched flavonoid biosynthesis-related pathways may contribute to the changes in flavonoid content. It further illustrated a close relationship between flavonoids and DEGs.

### Patterns of Expression of Flavonoid Biosynthetic Structural Genes

To further explore the molecular mechanism of accumulating flavonoids in *E. phaseoloides* stems, the genes related to flavonoids biosynthetic pathways were identified, and then the expression patterns of these genes were analyzed. A total of 34 structural genes of flavonoid biosynthesis were identified based on KEGG pathway annotations and KIPEs in this work, including PAL (3), C4H (3), 4CL (2), CHS (5), CHII (1), CHIIII (1), F3H (1), F3′H (4), F3′5′H (1), FHT (1), FNSII (1), FLS (3), DFR (2), ANS (2), LAR (2), and ANR (2) (Table 1). In addition, functionally relevant amino acid residues of most identified genes showed highly conservative, and no F3′5′H, FHT, ANR, and LAR candidates with conservation of all functionally relevant amino acids were detected (Supplementary Table 9). Most candidates showed high transcriptional activity at least in one stem developmental stage in our transcriptome data had a high gene expression level (TPM > 10). However, there were some isoforms of candidates that displayed very low transcript abundance at all the stages. It indicated that other isoforms may perform functions during the process of flavonoid biosynthesis in *E. phaseoloides* stems (Table 1).

**TABLE 1:** Candidates in the flavonoid biosynthesis of *E. phaseoloides*.

Gene_id	Gene_name	S1_TPM	S2_TPM	S3_TPM
Maker00007222	PAL_1	481.54	458.53	387.47
Maker00017096	PAL_2	209.03	178.75	281.66
Maker00024415	PAL_3	31.77	75.52	39.38
Maker00000730	C4H_1	113.46	325.01	236.68
Maker00016708	C4H_2	156.16	107.80	38.85
Maker00024431	C4H_3	9.57	24.52	6.57
Maker00005215	4CL_1	155.10	187.25	70.86
Maker00021972	4CL_2	2.24	2.45	1.33
Maker00016317	CHS_1	115.70	75.10	30.24
Maker00015698	CHS_2	87.03	240.23	153.12
BGI_novel_G000863	CHS_3	113.09	380.78	64.24
BGI_novel_G002275	CHS_4	322.93	149.11	43.15
BGI_novel_G002056	CHS_5	0.27	0.99	1.74
Maker00010747	CHII	20.69	70.02	7.72
Maker00010788	CHIII	15.23	18.21	20.11
Maker00011936	F3H	323.40	779.63	178.18
BGI_novel_G002728	F3′H_1	93.11	166.92	114.37
BGI_novel_G002452	F3′H_2	17.80	17.94	29.65
Maker00021903	F3′H_3	17.80	17.94	29.65
BGI_novel_G002451	F3′H_4	0.68	0.47	1.95
BGI_novel_G000284	F3′5′H	3.59	16.52	3.95
Maker00004594	FHT	0.17	0.46	3.20
Maker00012388	FNSII	1.14	1.47	11.36
Maker00001108	FLS_1	14.25	30.86	16.89
Maker00000877	FLS_2	0.31	0.15	2.48
Maker00008739	FLS_3	0.47	0.26	0.03
BGI_novel_G001177	DFR_1	105.39	511.09	88.79
Maker00011254	DFR_2	0.36	0.26	0.35
Maker00011665	ANS_1	28.83	74.64	9.08
BGI_novel_G000615	ANS_2	5.69	57.63	3.00
Maker00002624	LAR_1	59.19	113.37	145.87
Maker00020310	LAR_2	19.73	63.16	97.15
Maker00017345	ANR_1	71.18	186.12	49.29
Maker00019292	ANR_2	42.55	170.62	18.27

*Candidates are ordered by their position in the respective pathway. The expression level (mean TPM) of the candidates in different samples is shown: S1, S2, and S3. The background color showed the transcript abundance.*

**FIGURE 6:**
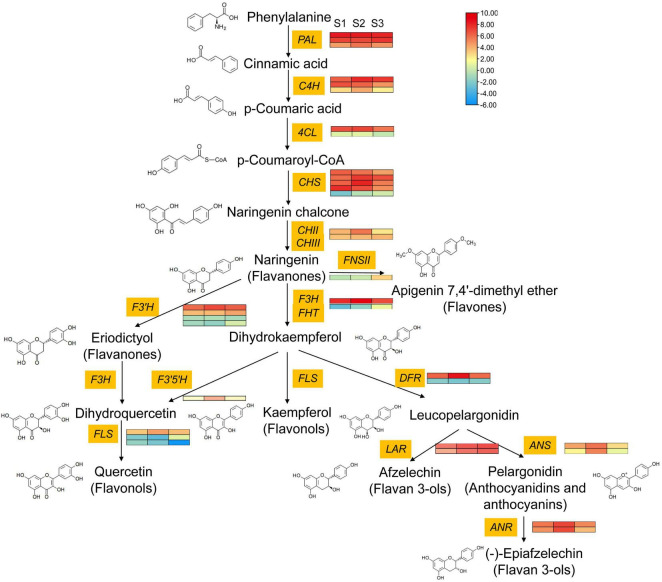
Comparative transcriptomic analysis of structural genes involved in the flavonoid biosynthesis pathway at different stems. The three columns for each gene represent the expression level at S1, S2, and S3 stage, respectively. Red represents the high expression level; blue represents the low expression level. Stem_young, stem_older, and stem_oldest were re-named as S1, S2, and S3, respectively.

Among them, there were 20 structural genes differentially expressed, including three C4H genes, one 4CL gene, four CHS genes, one CHII gene, one F3H, one F3′5′H, one FHT, one FNSII, one DFR, two LAR genes, two ANS gene, and two ANR gene (Supplementary Table 10 and Figure 6). Among these genes, two C4H (*C4H_1* and *C4H_3*) genes, two CHS genes (*CHS_2* and *CHS_3*), one CHII gene, one F3H gene, one F3′5′H gene, one DFR (*DFR_1*) gene, two ANS gene, two ANR gene, and one LAR (*LAR_2*) were obviously upregulated in S1 versus S2, and one CHS (*CHS_4*) was remarkably downregulated during all the stem developmental stage. However, in the comparison group of S2 and S3, most of the differentially expressed structural genes were significantly downregulated, including one 4CL (*4CL_1*), two C4H (*C4H_2* and *C4H_3*), two CHS genes (*CHS_3* and *CHS_4*), one CHII, one F3H, one F3′5′H, one DFR (*DFR_1*), two ANS, and two ANR. Only one FHT and two LAR were upregulated in S1 versus S3. FNSII gene was significantly upregulated during the late stem developmental stages. Most of the differentially expressed structural genes were significantly downregulated from S2 to S3 stage (Supplementary Table 10).

### Correlation Analysis Between Transcripts and Flavonoids

**FIGURE 7:**
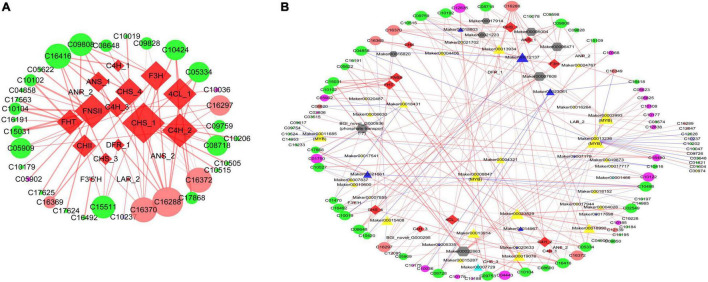
Connection network between flavonoid biosynthesis related genes and flavonoids. **(A)** Connection network between flavonoid biosynthesis structural genes and flavonoids; **(B)** connection network between flavonoids and MYBs, bHLHs, WDRs, transport genes, and structural genes. The magenta circle represents flavonols; the Indian red circle represents anthocyanins; the gray violet circle represents proanthocyanidins; the green circle represents other flavonoids; the red diamond represents the structural genes; the triangle represents TFs, the yellow triangle represents MYB families, the blue triangle represents bHLH families, the Caribbean green triangle represents WDR families; the gray hexagon represents transport genes. The size of the shapes in the graph shows the number of connections of the input genes and flavonoids in the network. The red line indicates a positive correlation, and the blue line indicates a negative correlation.

Many studies have already been performed to investigate the synthesis of flavonoids, and many structural and regulatory genes were identified in other plants (Falcone Ferreyra et al., 2012; Liu et al., 2020); it is still not clear which genes control the production of which flavonoids in *E. phaseoloides*. To reveal the regulatory network and candidate genes of flavonoid biosynthesis in *E. phaseoloides*, correlation analysis between the DAFs content and the expression of DEGs was performed. All detected correlations between DAFs and DEGs are shown in Supplementary Table 11. There were 34 flavonoids that showed higher correlation coefficient values (*r* > 0.8, *p*-value < 0.05) with 17 differentially expressed flavonoid biosynthesis structural genes (Supplementary Table 12), and their interaction networks are organized in Figure 7A. The 17 structural genes contained one 4CL, three C4H, three CHS, one CHII, one F3H, one F3’5’H, one FHT, one FNSII, one DFR, one DFR, two ANS, and one ANR, which are the major structural genes in the flavonoid biosynthesis pathway. The 34 flavonoids include seven flavones, six anthocyanins, four biflavonoids and polyflavonoids, three flavanones, three pterocarpans, two chalcones, two flavonols, one dihyroflavonol, one flavan 3,4-diol, one dihydrochalcone, one isoflavan, one isoflavone, one flavonoid glycoside, and one proanthocyanidin (Supplementary Table 12).

The previous research showed that many MBW complexes are involved in regulating flavonoid synthesis (Ramsay and Glover, 2005; Xu et al., 2015; Xu et al., 2021). In this study, the expression of 47 genes encoding MYB, bHLH, and TRANSPARENT TESTA GLABRA 1 (TTG1) homologs was highly correlated with the amount of 81 flavonoids, including 32 MYBs, 13 bHLHs, and 2 TTG1 homologs (Supplementary Table 13). Furthermore, the expression level of 86 transporters was also showed highly associated with quantitative changes of 76 flavonoids (Supplementary Table 14). The solute carrier family, glutathione *S*-transferase, ABC transporter B family, and multidrug resistance protein were the most members in the 86 genes. It was in accordance with the genes involved in flavonoid transport observed in pepper fruit (Liu et al., 2020). The previous studies found that H^+^-ATPase are associated with flavonoid uptake efficiency in the vacuoles and cytoplasm (Kitamura, 2006). Mutations of P-type H^+^-ATPase (AHA10) lead to vacuolar morphology defects and reduced proanthocyanidin content in Arabidopsis seeds (Baxter et al., 2005). Maker00021223 and Maker00005004, a member of the H^+^-transporting ATPase, are closely positively related to one flavonol (3-*O*-methylquercetin) and one flavone (tricetin), two anthocyanidins (pelargonidin 3-O-[6-caffeoyl-beta-D-glucoside] and delphinidin 3-[6-*p*-coumaroyl] glucoside), one chalcone (isobavachalcone), one dihydrochalcone (isouvaretin), and one pterocarpan (glyceollin III), respectively.

In addition, the phosphate transporters 1–7 (BGI_novel_G000936 and BGI_novel_G000295) were significantly positively related to one flavanone (isosakuranetin), three flavanones (acacetin, luteolin 7-*O*-glucuronide, and morusinol), three flavonols (quercitrin, 8-hydroxykaempferol, and kaempferol 3-*O*-beta-D-glucosylgalactoside), two flavones (acacetin and morusinol), and one anthocyanidin (cyanidin 3-*O*-[6-*O-p*-coumaroyl] glucoside), respectively. The TRANSPARENT TESTA 12-like gene (Maker00022863) is *obviously* positively associated with five flavones (nortangeretin, 6-methoxyluteolin 7-rhamnoside, lucenin-2, malonylapiin, and apiin), four flavonols (quercetin 3-*O*-glucoside, robinetin, rutin, quercetin 3-*O*-[6-*O*-malonyl-beta-D-glucoside], and myricitrin), and one flavanone (silandrin). The previous studies have shown that TRANSPARENT TESTA genes are associated with flavonoid accumulation (Appelhagen et al., 2011; Appelhagen et al., 2015). The results indicated that the transporter genes are conserved in plants.

The correlation regulatory network of some significant DAFs and structural gene, MYBs, bHLH, TTG1 homologs, and key transporter genes in each comparison group is displayed in Figure 7B (Supplementary Table 13). In this coexpression network, many hub genes were identified, including structural genes, MYBs (Maker00011685, Maker00013236, Maker00003993, and Maker00008847), bHLHs, TTG1 homologs, and transporter genes, which correlated with multiple different flavonoids. *FHT* (Maker00004594) and *FNSII* (Maker00012388) are positively correlated with 6-methoxyluteolin 7-rhamnoside, lucenin-2, malonylapiin, nortangeretin, and 3-methoxyapigenin. *CHS_4* gene (BGI_novel_G002275) is positively correlated with pelargonidin 3-O-3′′,6′′-*O*-dimalonylglucoside, delphinidin, 3-(6-*p*-coumaroyl) glucoside, isobavachalcone, phellamurin, apigenin 7,4′-dimethyl ether, and desmethylxanthohumol. *EpMYB41* (Maker00003993) is positively correlated with seven flavonols, four flavones, one anthocyanidin, and one dihydrochalcone. Maker00007729 is belonged to WDR protein and was the homolog of *AtTTG1*-like (*AtAN11*). The amino acid identity between them was 92.8%. Maker00007729 is positively correlated with one dihyroflavonol and two flavonols. Some MYBs genes clustered in the subgroup of confirmed flavonoid biosynthesis-regulating *Arabidopsis* MYB family (Figure 3 and Supplementary Table 13), which also significantly correlated with flavonoids in this study. *EpMYB84* (Maker00010600, *AtMYB4*) and *EpMYB76* (Maker00016264, *AtMYB123* [*AtTT2*]) are positively correlated with one anthocyanin (pelargonidin 3-*O*-beta-D-glucoside 5-*O*-[6-coumaroyl-beta-D-glucoside]) and two isoflavones (luteone, sayanedine), respectively (Figure 7B and Supplementary Table 13). *EpMYB135* (Maker00004321, *AtMYB5*) is positively correlated with two isoflavones (luteone, sayanedine) and negatively correlated with one flavonol (quercetagetin). *EpMYB64* (Maker00021702, *AtMYB82*) is negatively correlated with one dihydrochalcone (isouvaretin). *EpMYB51* (Maker00018152, *AtMYB12* [*PFG1*]) is positively correlated with one flavone (6-hydroxy-4′,5,7-trimethoxyflavone).

Furthermore, there were also exist one metabolite associated with multiple genes (Figure 7B and Supplementary Table 13). The expression level of Maker00007729 (*AtTTG1-like* [*AtAN11*]), *EpMYB41* (Maker00003993, *AtMYB36*), EpMYB119 (Maker00013914, *AtMYB4*), *EpMYB118* (Maker00013934, *AtMYB44*), and *EpbHLH53* (Maker00018803, IIIb, *AtbHLH116*) was showed highly associated with the content of kaempferol 3-sophorotrioside (flavonols). Meanwhile, the accumulation of glepidotin B (dihyroflavonol) was also positively corrected with the expression of Maker00007729 (*AtTTG1-like* [*AtAN11*]), EpMYB119 (Maker00013914, *AtMYB4*), and *EpbHLH53* (Maker00018803, IIIb, *AtbHLH116*). The structure genes [*CHS_1* (Maker00016317), *CHS_4* (BGI_novel_G002275), *CHII* (Maker00010747), *F3H* (Maker00011936), *ANS_1* (Maker00011665)], *EpbHLH128* (Maker00012137, IIIf, *AtbHLH2*), and transport genes [ABC transporter B family (Maker00006471), H^+^-transporting ATPase (Maker00005004)] are positively correlated with delphinidin 3-(6-*p*-coumaroyl) glucoside (anthocyanin). It suggested these genes may coregulated the biosynthesis of flavonols and anthocyanins.

## Discussion

### Analysis of Flavonoids in *E. phaseoloides* Stem

The specialized metabolism of *legumes* mainly includes terpenoids, flavonoids, and alkaloids (Wink, 2013; de Souza et al., 2019). As a member of the Leguminous family, many previous studies on *E. phaseoloides* metabolites have focused on terpenoids (Iwamoto et al., 2012; Xiong et al., 2013; Xiong et al., 2014). In our study, it was shown that flavonoids are also the main metabolites in *E. phaseoloides* stem by untargeted metabolite analysis (Supplementary Figure 1). The number of detected flavonoids type from *E. phaseoloides* stems was far more than previously reported (Zhao et al., 2011; Dong et al., 2012). It suggested that untargeted metabolite analysis can be applied for the comprehensively detected phytochemical compounds in the absence of knowledge of chemical components. In addition, although the types of flavonoids in different developmental stages of stem were the same, the content of compounds showed a markedly different, particularly between the young stem and the old stem. It suggested that flavonoids exhibited quantitative pattern between different developmental stages in *E. phaseoloides* stem. Thus, the metabolic profiling of flavonoids can provide a theoretical basis for selecting the appropriate development stage of stem to collect samples, to obtain more accumulation of active ingredients from *E. phaseoloides.* However, accurate characterization and quantification of flavonoids in *E. phaseoloides* require targeted metabolomics and more standards in future.

### Analysis of Structural Genes, Transcription Factors in *E. phaseoloides* Stem

Flavonoids are synthesized by many pathways (including phenylpropanoid biosynthesis, flavonoid biosynthesis, anthocyanin biosynthesis, isoflavonoid biosynthesis, and flavone and flavonol biosynthesis) and are regulated by many structural genes (Koukol and Conn, 1961; Russell and Conn, 1967; Jez et al., 2000; Jun et al., 2018; Wu et al., 2020). Herein, thirty-four structural genes involved in flavonoid biosynthesis were identified (Table 1). Functionally relevant amino acid residues of most identified genes showed highly conservative. Hydroxylation of the 5′ position by F3′5′H is a particularly important step, which determines the B-ring tri-hydroxyl flavonoid end-product (epigallocatechin-3-gallate or delphinidin) synthesized in plants (Bailey et al., 2003). The F3′5′H candidate showed 60.36% similarity to previously characterized *Glycine max* F3′5′H sequences, but lacking functionally relevant amino acids P33, P34, P36, and P40, and one substitution of an amino acid residue in R/T389. The lacked functionally relevant amino acids may be due to the obtained transcript sequence is incomplete. Thus, it needs additional confirmation.

Dihydroflavonol is the common precursor for flavonol, anthocyanin, and proanthocyanin. Flavonol biosynthesis is competing with anthocyanin or proanthocyanidin biosynthesis. The anthocyanin or proanthocyanidin biosynthesis rate-limiting genes were significantly upregulated in the S2 stage: (*DFR_1* (log2Ratio[S2/S1]: 2.23), *ANS_1* (log2Ratio[S2/S1]: 1.35), *ANS_2* (log2Ratio[S2/S1]: 3.03), *LAR_2* (log2Ratio[S2/S1]: 1.58), *ANR_1* (log2Ratio[S2/S1]: 1.37), *ANR_2* (log2Ratio[S2/S1]: 1.86)). Although flavonol biosynthesis key gene *FLS_1* was upregulated, the *p*-value is greater than 0.05 and the expression level was obviously lower than anthocyanin or proanthocyanidin biosynthesis key genes (Table 1 and Supplementary Table 10). Thus, it speculated that the anthocyanin or proanthocyanidin biosynthesis particularly anthocyanin biosynthesis was more active at the S2 stage. In addition, FNSII was significantly upregulated in the S3 stage (log2Ratio[S3/S2): 2.88) (Table 1 and Supplementary Table 10), which indicated the flavones biosynthesis was active at this stage. In brief, with the development of *E. phaseoloides* stem, flavonol, anthocyanin, and proanthocyanin biosynthesis were first active, and the anthocyanin or proanthocyanin biosynthesis branch was dominant. Subsequently, the flavones biosynthesis branch was active at the late developmental stage of the stem.

In addition to structural genes, TFs also play important roles in flavonoid biosynthesis (Baudry et al., 2004; Davies et al., 2012; Lloyd et al., 2017; Liu et al., 2021). As two types of the most reported TFs regulating flavonoid synthesis, the MYBs and bHLHs were identified and classified. The number and type of MYB genes identified in *E. phaseoloides* were in the same range as previously reported in other plant species, but no MYB4R proteins (Stracke et al., 2001; Pucker et al., 2020b). As showed in MYB phylogenetic tree (Figure 3), a total of 11 R2R3-MYBs, one R1-MYB, and one 3R-MYB were closely clustered into the clades of flavonoid biosynthesis-related *Arabidopsis* MYBs. Among them, eight MYBs (*EpMYB108*, *EpMYB111*, *EpMYB84*, *EpMYB85, EpMYB78, EpMYB115*, *EpMYB86*, and *EpMYB76*) were in the same clade of *AtMYB75*/*AtMYB90*/*AtMYB113*/*AtMYB114*, which have been confirmed to be related to anthocyanin biosynthesis (Borevitz et al., 2000; Teng et al., 2005; Gonzalez et al., 2008), and three MYBs (*EpMYB5*, *EpMYB135*, *EpMYB64*) and two MYBs (*EpMYB51* and *EpMYB122*) were assigned into a big clade of *AtMYB123* and *AtMYB11*/*AtMYB12*/*AtMYB111*, which have been confirmed to regulate the biosynthesis proanthocyanidin (Nesi et al., 2001; Hancock et al., 2012) and flavonol (Stracke et al., 2007; Stracke et al., 2010), respectively. The results indicate that these MYBs may have the potential ability to regulate the biosynthesis of flavonoids.

Most bHLHs involved in regulating anthocyanin biosynthesis are belonged to subgroup III, which is functionally conserved and has been demonstrated to participate in regulating plant development and defense response (Ludwig et al., 1989; Bailey et al., 2003; Heim et al., 2003; Song et al., 2021). A total of 23 subgroup III bHLHs were noted in this study, including nine subgroup IIId+e, eight subgroup IIIb, three subgroup IIIf, two subgroup IIIc, and one subgroup IIIa. It suggested that *similar bHLHs regulatory* for regulation of flavonoid synthesis may exist in E. *phaseoloides*. These predicted genes and related information will facilitate research on further investigations of the potential function of these genes in *E. phaseoloides* and other plants.

### Analysis of Key Candidate Genes Involved in Flavonoids Accumulation in *E. phaseoloides* Stem

At present, transcriptome and metabolome combined analysis has been widely used to explore the relationships between the expression of genes and metabolites related to biosynthetic pathways, so as to obtain metabolites-related functional genes and regulator networks (Liu et al., 2020; Du et al., 2021; Fu et al., 2021; Hou et al., 2021; Zou et al., 2021; Wu et al., 2022). The transcriptome and metabolome *association analysis* results showed that some transporter genes were also obviously positively associated with various types of flavonoids. For example, the TRANSPARENT TESTA protein [Maker00022863] was the homolog of TRANSPARENT TESTA 12-like (TT12-like), encoding a multidrug and toxin extrusion (MATE) transporter, which is involved in proanthocyanidin biosynthesis in *Arabidopsis* seed coat and *Medicago truncatula* hairy roots (Debeaujon et al., 2001; Marinova et al., 2007; Zhao and Dixon, 2009). It is positively correlated with five flavonols, five flavones, and one flavanone accumulation in this study. It indicated that the TT12-like gene may be involved in other flavonoids transport, such as flavonols, flavones, and flavanones.

As shown in the phylogenetic tree results (Figure 3), *EpMYB84* (Maker00010600, *AtMYB4*) and *EpMYB76* (Maker00016264, *AtMYB123* [*AtTT2*]), *EpMYB135* (Maker00004321, *AtMYB5*), *EpMYB64* (Maker00021702, *AtMYB82*), and *EpMYB51* (Maker00018152, *AtMYB12* [*PFG1*]) were closely clustered into the clade of anthocyanin and proanthocyanidin or flavanols biosynthesis-related *Arabidopsis* MYBs, respectively. *EpMYB84* and *EpMYB76* are positively correlated with one anthocyanin and two isoflavones, respectively. However, *EpMYB135* is positively correlated with two isoflavones and negatively correlated with one flavonol. *EpMYB64* is negatively correlated with one dihydrochalcone. *EpMYB51* is positively correlated with one flavone. It is speculated that these genes may regulate different types of flavonoids with different additional regulators.

Moreover, there was also existed one metabolite associated with multiple genes. A previous study of *TTG1*-like (also known as *AtAN11*, Light-regulated *WD1*, or *LWD1*) indicated that it acts as a clock protein, contributing to the regulation of circadian period length and photoperiodic flowering (Wu et al., 2016). AtMYB4 has been shown to act as a transcriptional repressor for the expression of early phenylpropanoid genes *C4H* (Jin et al., 2000). Our correlation results showed that the Maker00007729 (*AtTTG1-like* [*AtAN11*]) is positively regulated glepidotin B (dihyroflavonol) and kaempferol 3-sophorotrioside (flavonol) biosynthesis by interaction with bHLH proteins such as EpbHLH53 (Maker00018803, IIIb, AtbHLH116) and EpMYB119 (Maker00013914, AtMYB4). These findings suggested that the WDR gene *TTG1-like* (*AN11*) regulated dihydrochalcones and flavonol biosynthesis in specific combinations with IIIb bHLH and R2R3MYBs in *E. phaseoloides* stem.

This was the first report for flavonoid synthesis in the genus *Entada*. Our data suggested that some genes might be regulated together as a regulator network involved in flavonoid biosynthesis, particularly flavonols and anthocyanins in *E. phaseoloides* stems. Meanwhile, it also indicated that some genes regulate not only one type of flavonoid but several distinct subclasses of flavonoid biosynthesis. Taken together, these results indicated that the process of flavonoids metabolism keeps notably activated during the development of stems in *E. phaseoloides*, and it is a complicated process coregulated by multiple genes. However, these results were based on the bioinformatic analysis data. Experimental verification such as transgenic studies will be needed to confirm the roles of these genes in flavonoid biosynthesis in *E. phaseoloides* in the future.

In spite of this, this study offered candidate genes involved in flavonoid biosynthesis and characteristics of flavonoids accumulation, improved our understanding of the MYBs and bHLHs-related regulation networks of flavonoid biosynthesis in *E. phaseoloides* stem, and also provided references for the metabolic engineering of flavonoid biosynthesis in *E. phaseoloides* stem.

## Data Availability Statement

The sequencing raw data have been deposited to China National GeneBank (CNGB: https://db.cngb.org) with project accession ID: CNP0002249 (S1: CNR0454679, CNR0454680, and CNR0454681; S2: CNR0454682, CNR0454683, and CNR0454684) and CNP0001900 (S3: CNR0390213, CNR0390214, and CNR0390215).

## Author Contributions

ZM designed the project. ML accomplished the experiments. ZZ helped with sample treatment and data analyzing. ML wrote and revised the manuscript. All authors read and agreed to the final manuscript.

## Conflict of Interest

The authors declare that the research was conducted in the absence of any commercial or financial relationships that could be construed as a potential conflict of interest.

## Publisher’s Note

All claims expressed in this article are solely those of the authors and do not necessarily represent those of their affiliated organizations, or those of the publisher, the editors and the reviewers. Any product that may be evaluated in this article, or claim that may be made by its manufacturer, is not guaranteed or endorsed by the publisher.

## Footnotes

1
https://github.com/BGI-flexlab/SOAPnuke/

2
http://daehwankimlab.github.io/hisat2/

3
http://ccb.jhu.edu/software/stringtie/

4
https://github.com/bpucker/KIPEs
