# Supplementary figures and images for: Integrative Analysis of Metabolome and Transcriptome Identifies Potential Genes Involved in the Flavonoid Biosynthesis in Entada phaseoloides Stem
Source: Front Plant Sci. 2022 May 10;13:792674. doi: 10.3389/fpls.2022.792674 (PMC9127681; doi:10.3389/fpls.2022.792674)

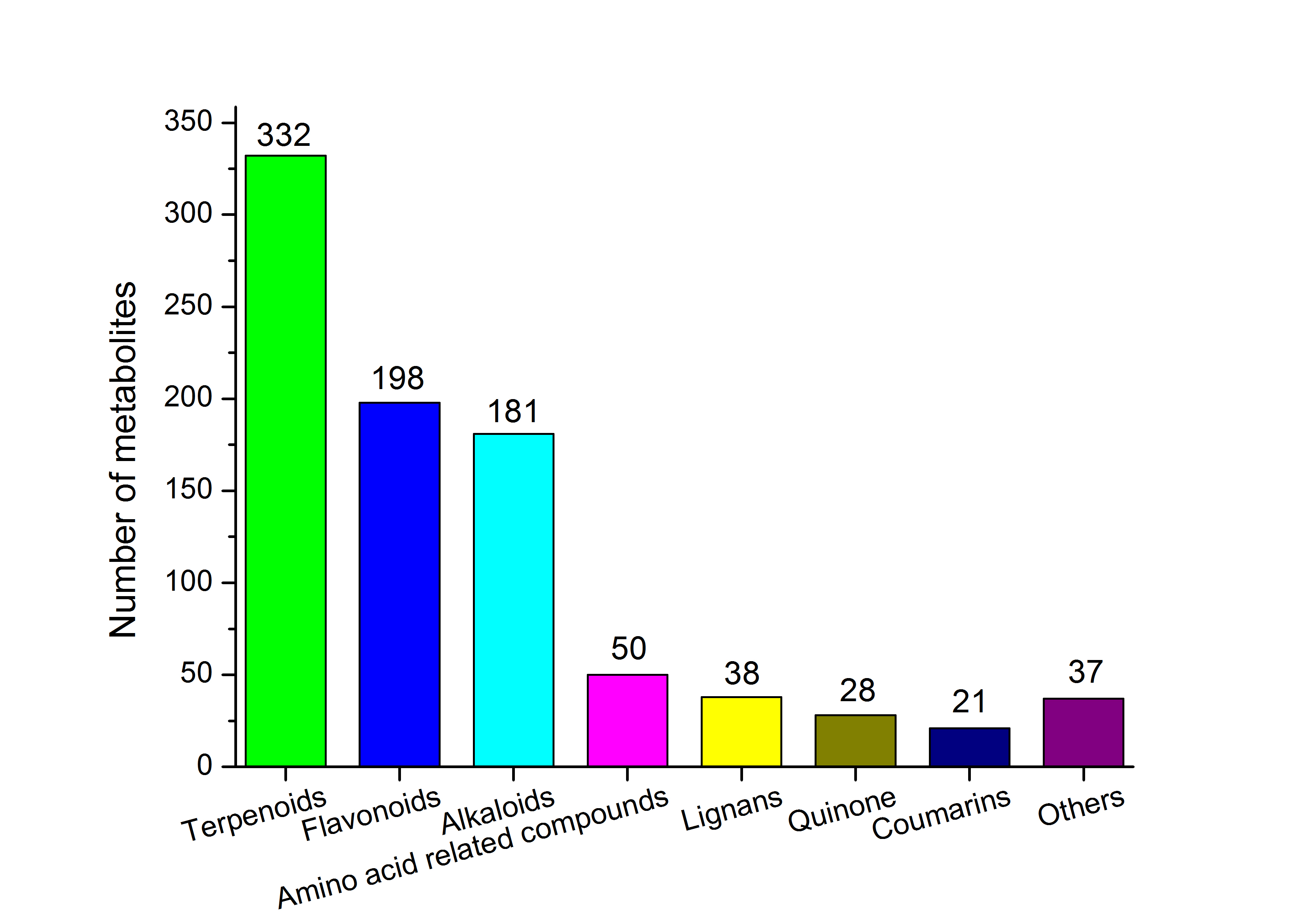

Supplement: Supplementary file 1 [file Image_1.JPEG]

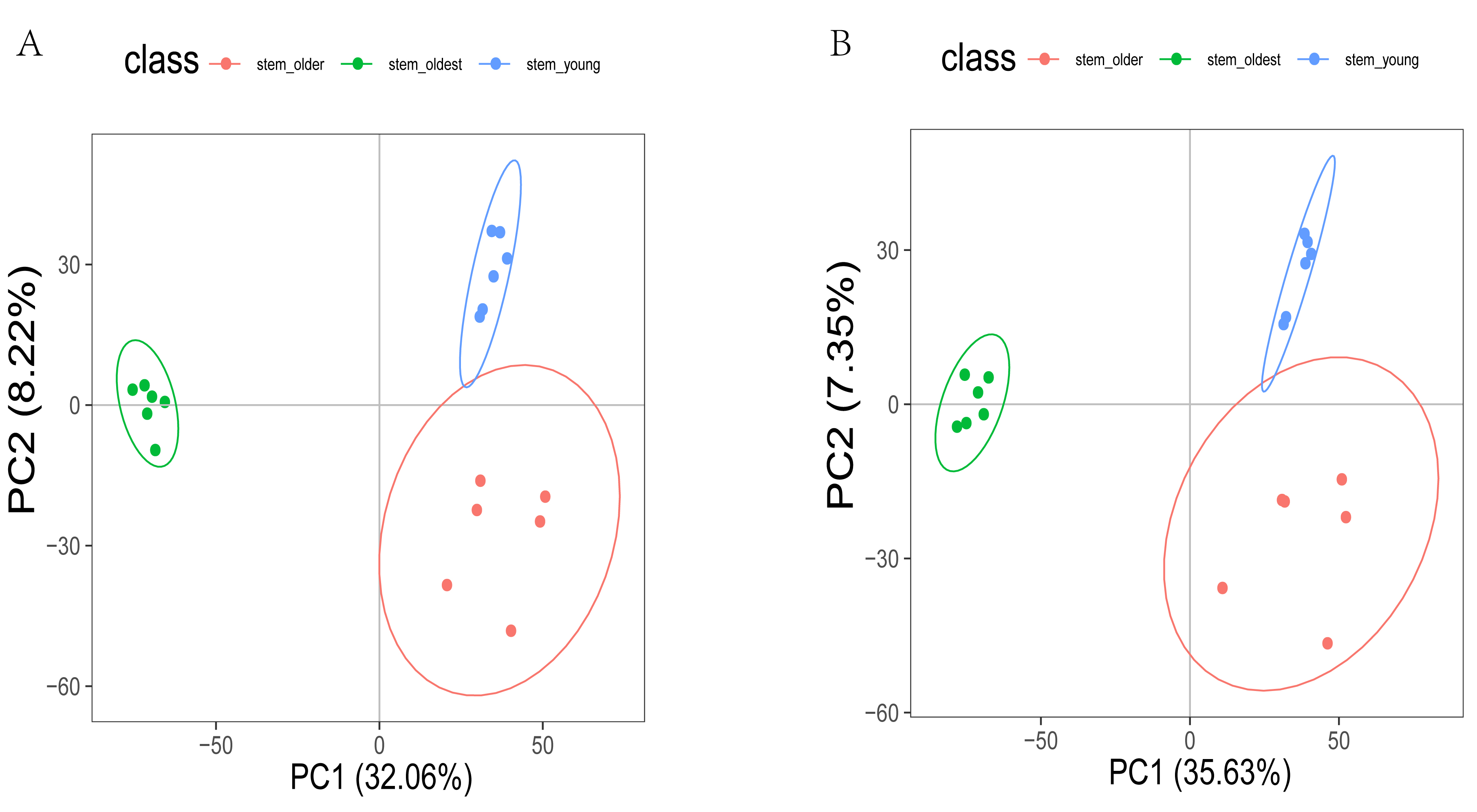

Supplement: Supplementary file 2 [file Image_2.JPEG]

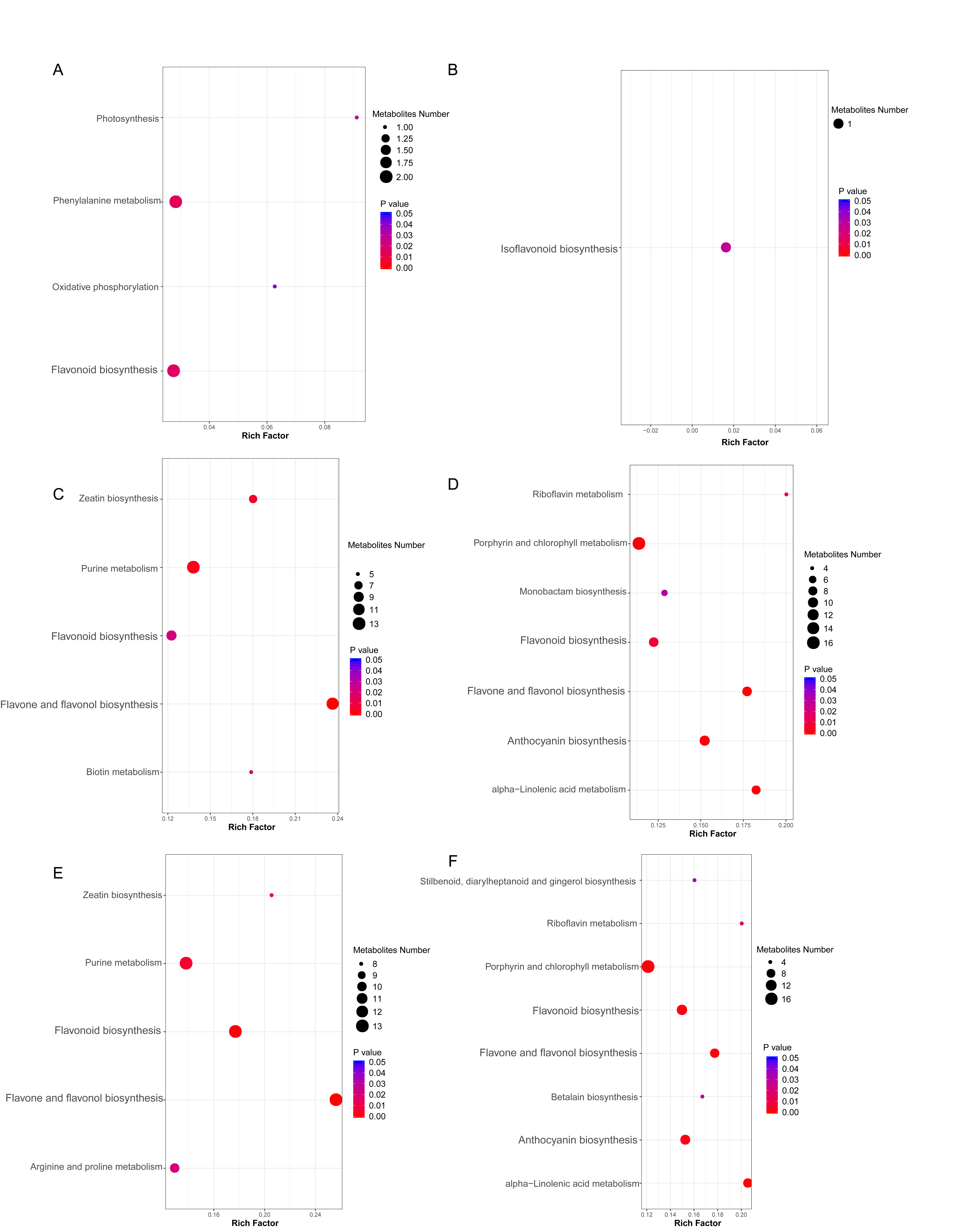

Supplement: Supplementary file 3 [file Image_3.JPEG]

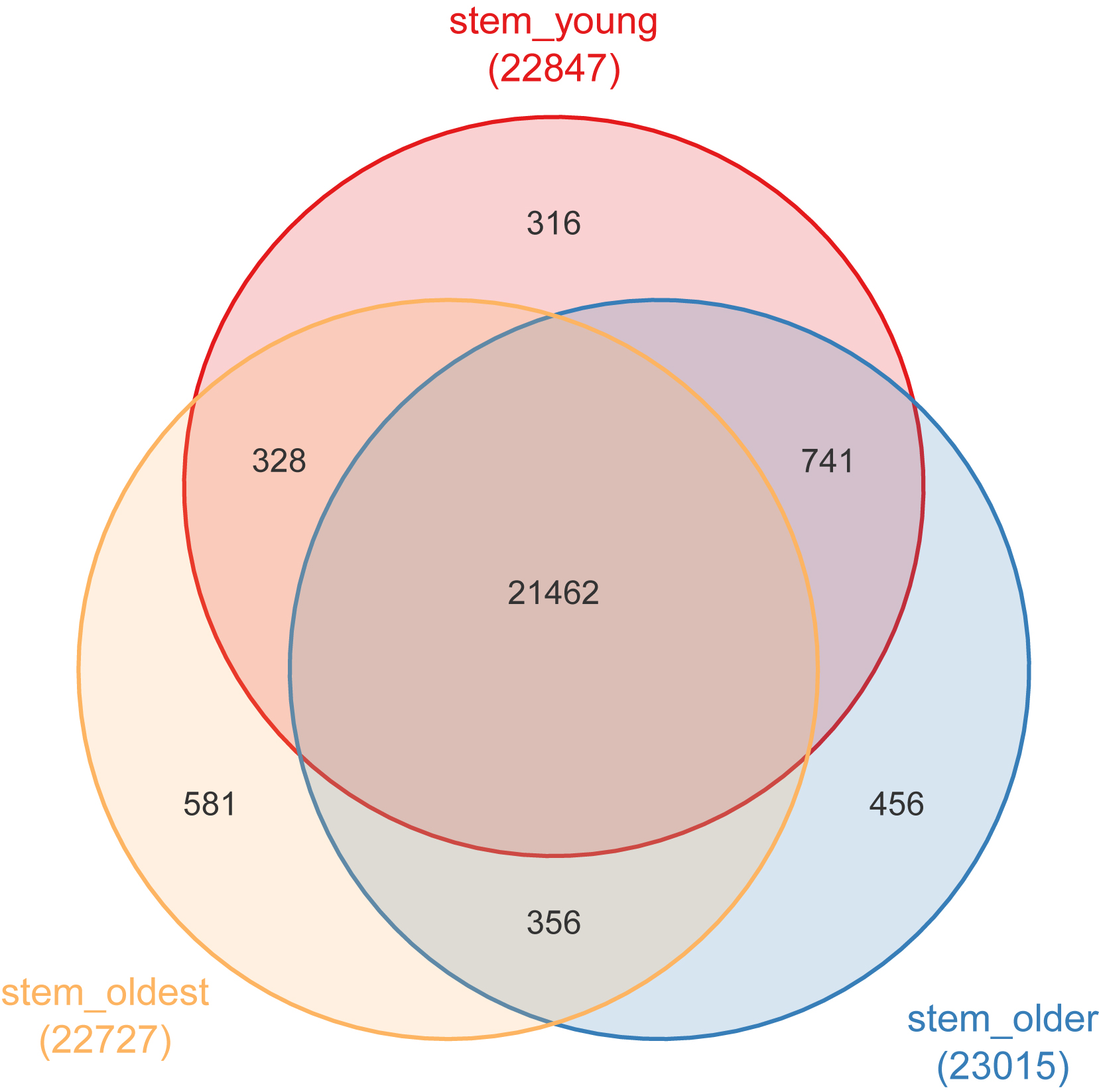

Supplement: Supplementary file 4 [file Image_4.JPEG]
