# Supplementary material for: Integrative Analysis of Metabolome and Transcriptome Identifies Potential Genes Involved in the Flavonoid Biosynthesis in Entada phaseoloides Stem
Source: Front Plant Sci. 2022 May 10;13:792674. doi: 10.3389/fpls.2022.792674 (PMC9127681; doi:10.3389/fpls.2022.792674)
Supplement: Supplementary file 5 [file Table_1.DOCX]

**Supplementary File 1.** The MS/MS spectrum of rutin, myricitrin and acacetin in authentic standard and the *E. phaseoloides* stem.

1. Rutin (neg)

| Standard m/z | Characteristic Peak Intensity | Stem sample m/z | Characteristic Peak Intensity |
| --- | --- | --- | --- |
| 271.0249 | 1.84E+04 | 271.0286 | 5.35E+03 |
| 300.0298 | 6.91E+05 | 300.0315 | 1.87E+05 |


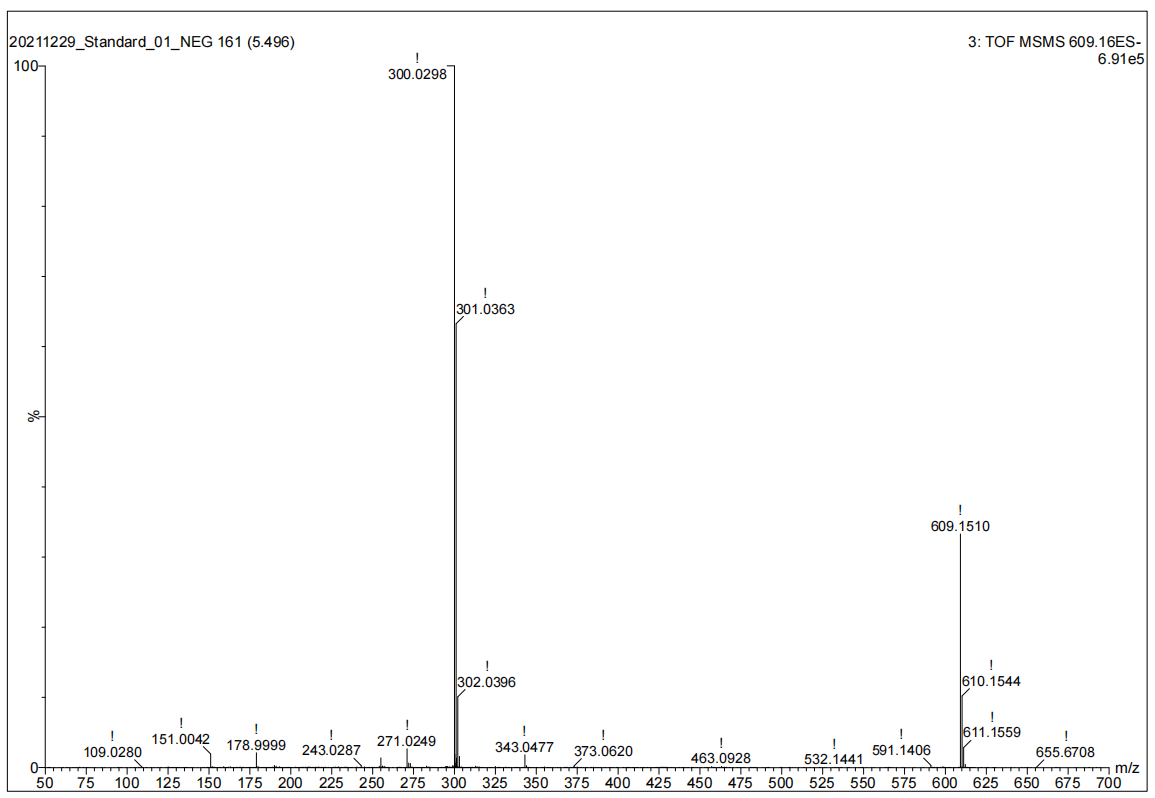


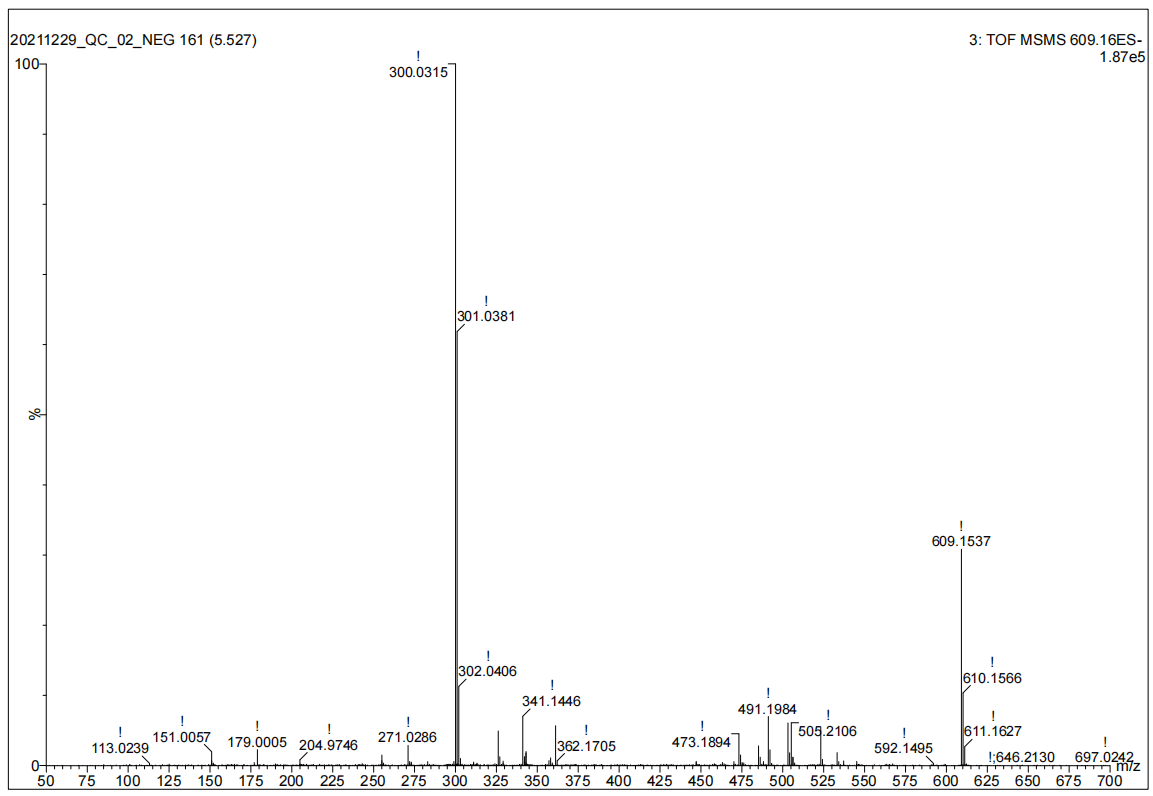


1. Myricitrin (neg)

| Standard m/z | Characteristic Peak Intensity | Stem sample m/z | Characteristic Peak Intensity |
| --- | --- | --- | --- |
| 178.9995 | 1.70E+04 | 179.0034 | 6.30E+02 |
| 316.0251 | 1.08E+06 | 316.0263 | 4.48E+04 |


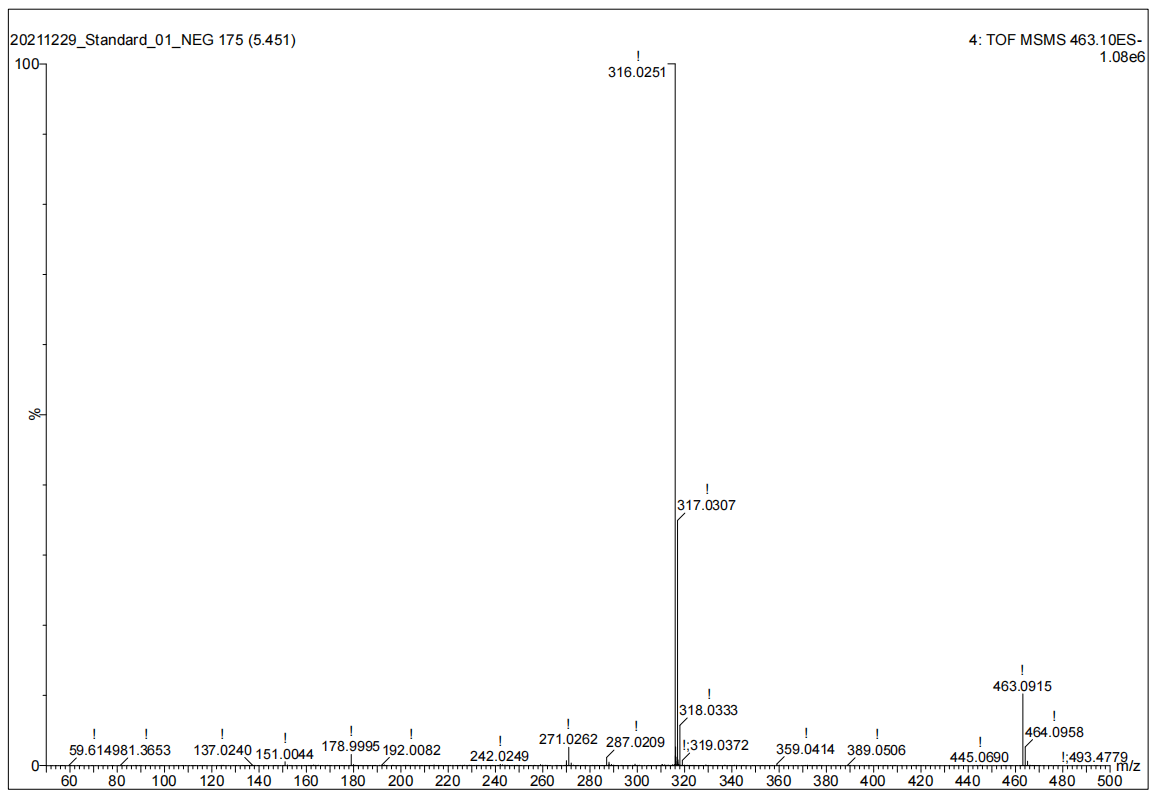

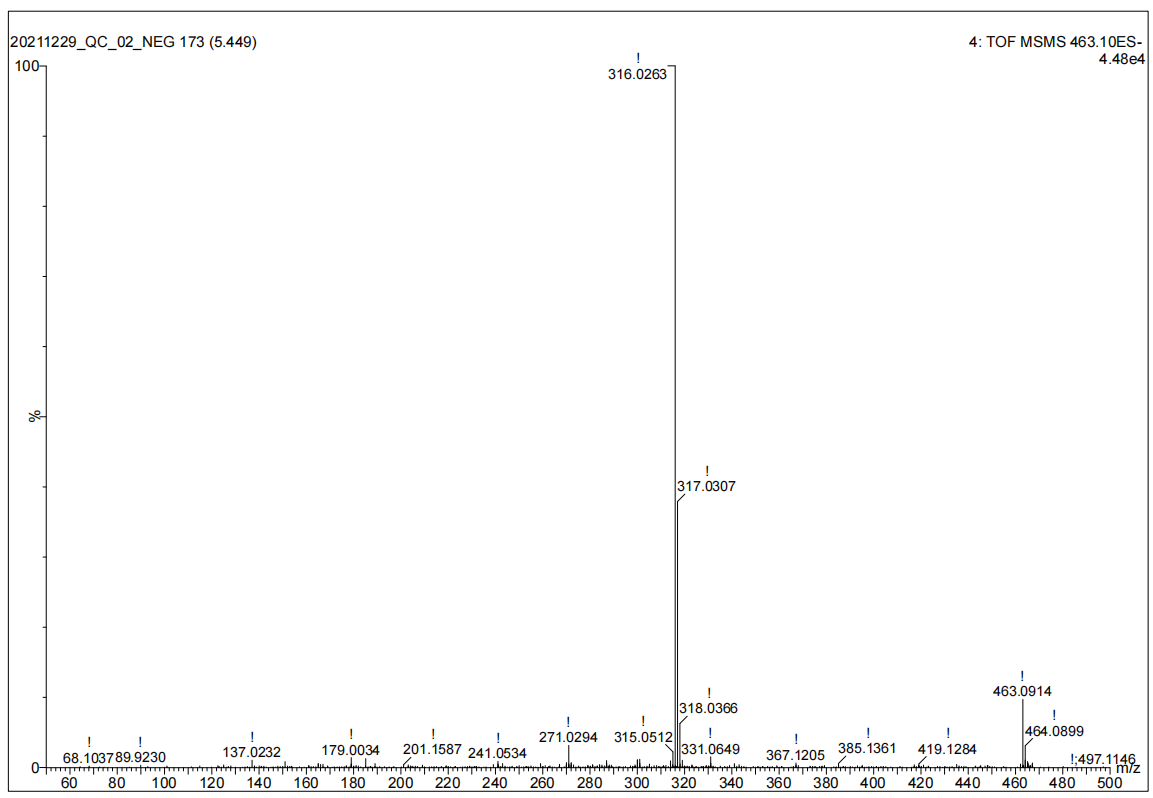


1. Acacetin (pos)

| Standard m/z | Characteristic Peak Intensity | Stem sample m/z | Characteristic Peak Intensity |
| --- | --- | --- | --- |
| 133.0648 | 7.08E+04 | 133.0661 | 2.21E+03 |
| 243.0611 | 3.48E+05 | 243.0622 | 2.04E+04 |


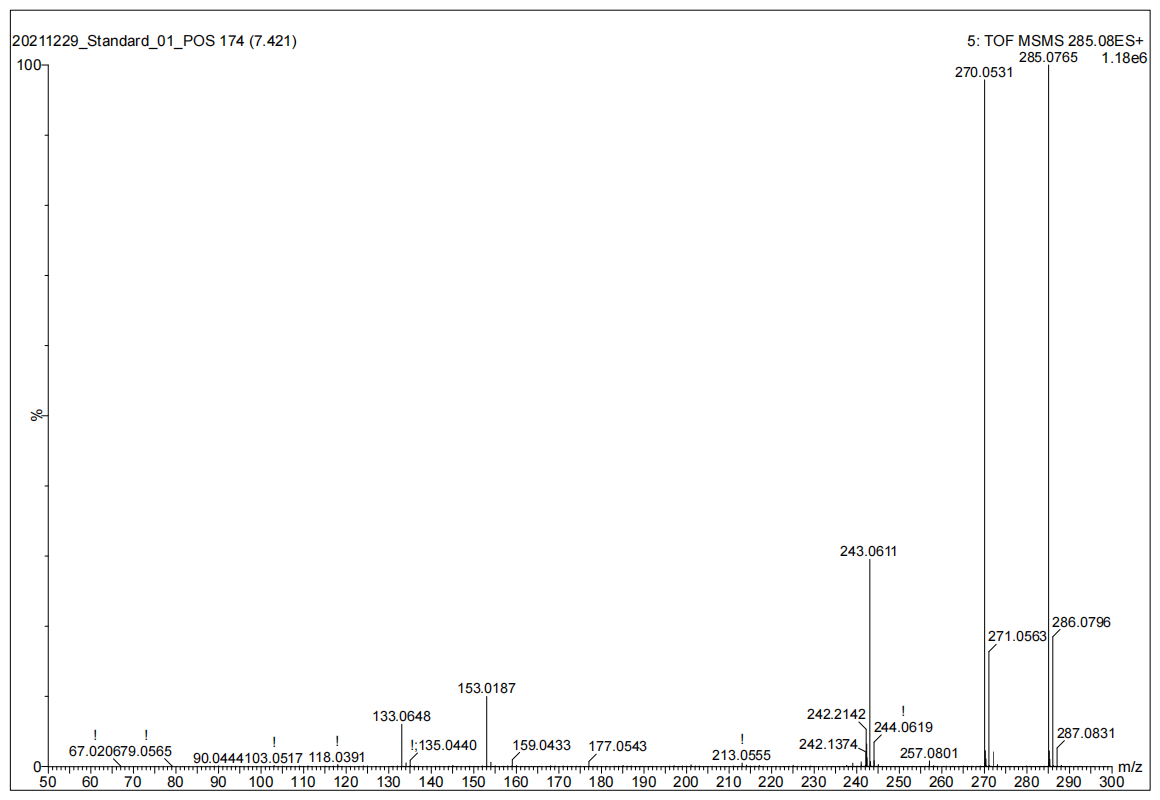

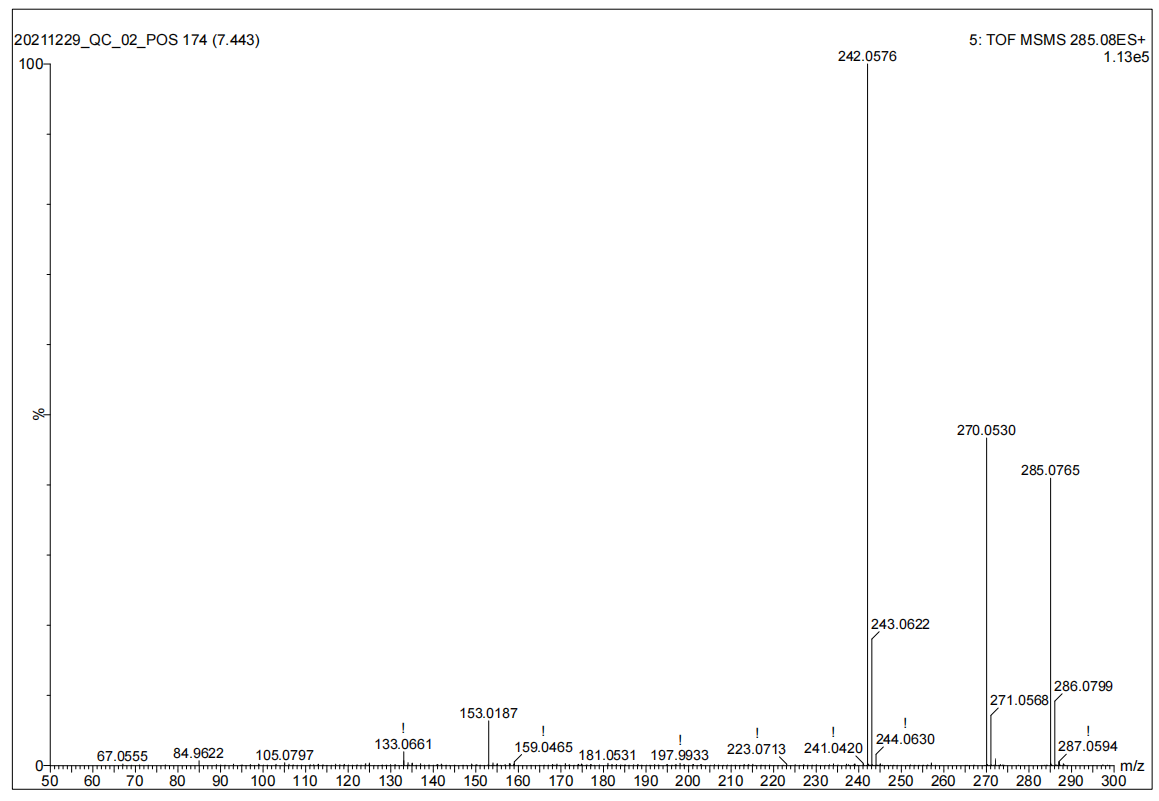


1. Myricitrin (pos)

| Standard m/z | Characteristic Peak Intensity | Stem sample m/z | Characteristic Peak Intensity |
| --- | --- | --- | --- |
| 303.0433 | 7.01E+02 | 303.0495 | 2.43E+04 |
| 319.0454 | 1.15E+05 | 319.0458 | 3.13E+03 |


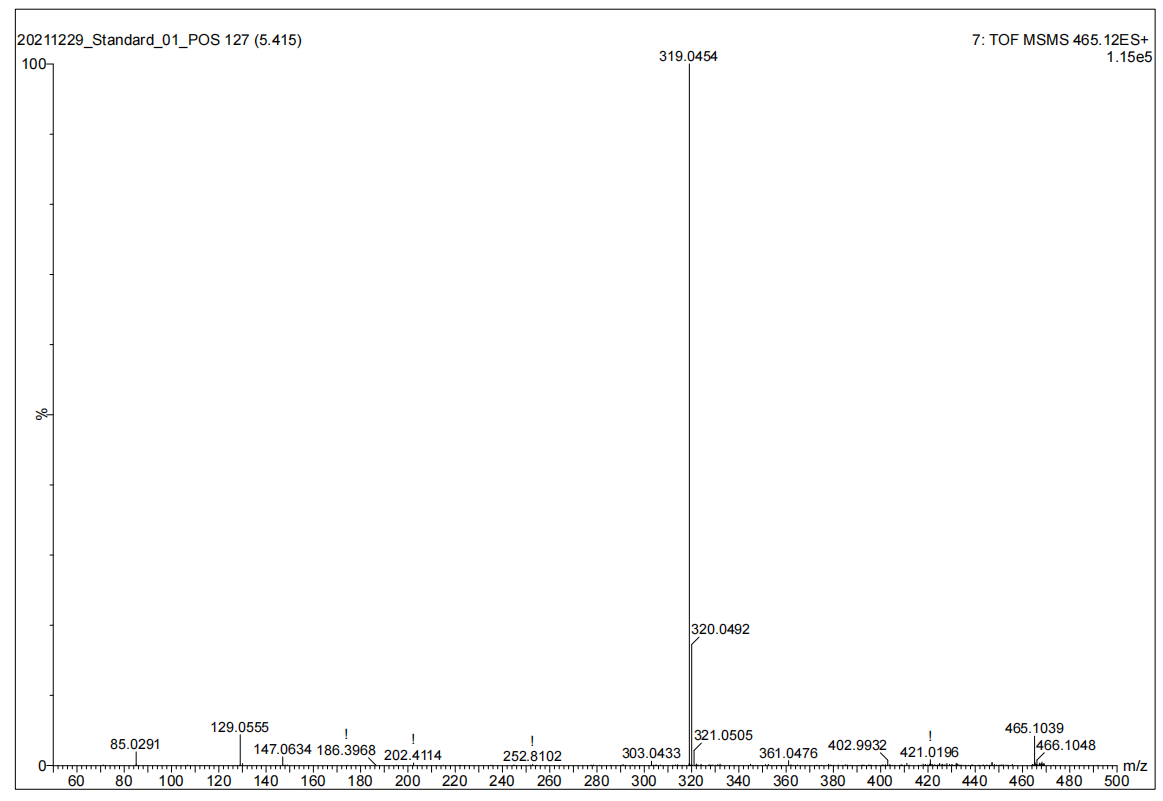


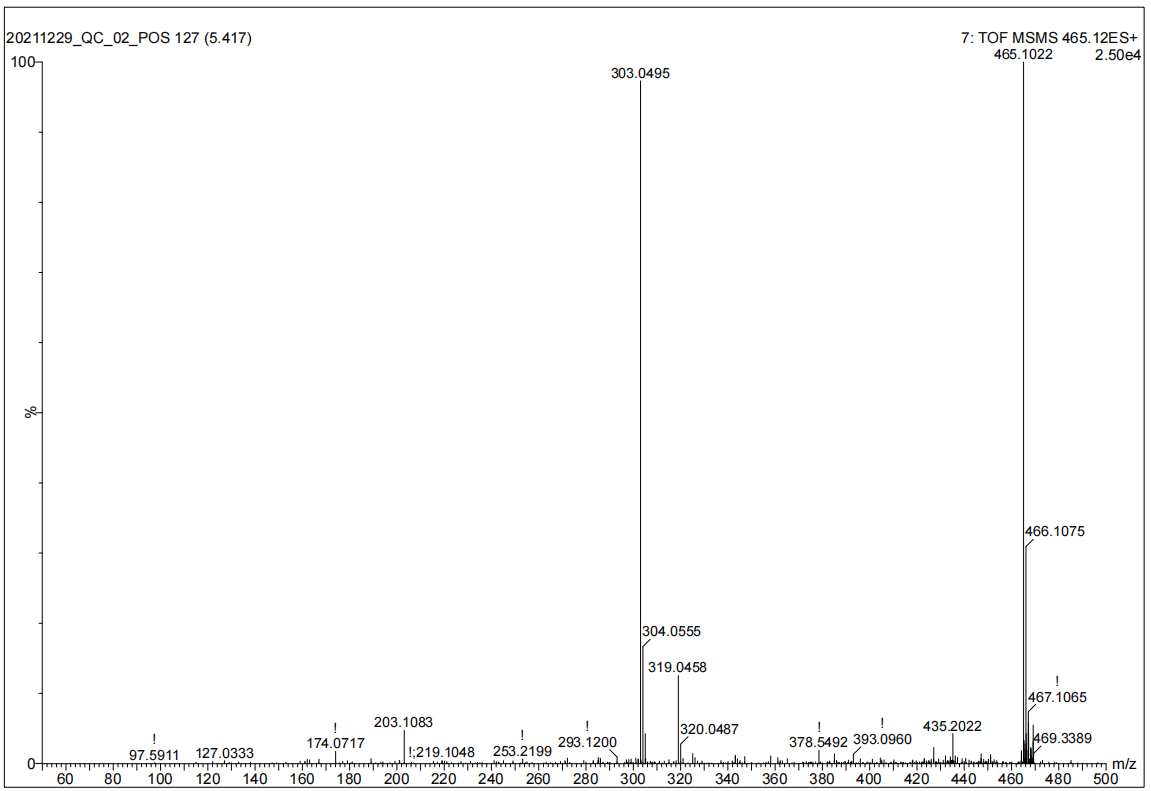


**Supplementary File 2.** The sequences of MYBs, bHLH and WDRs identified in the *E. phaseoloides*

>Maker00001333 EpMYB1

MANSNNHNDAENKEHVLLPIIGTDEGDAGEGAGGAPAVAEEMALKKGPWTAAEDAVLIDYVTKHGEGNWNAVQKNTRLARCGKSCRLRWTNHLRPNLKKGAFSPEEEKLILELHAQFGNKWARMATLLPGRTDNEIKNYWNTRVKRRQRQGLPLYSNDSDQPTTSAAPTASATRPTTGSTATKFEYCHQNQYPLSSPHSGSSYSTHTPFLDPSSLSSSSSFLSFPIHQPSSLHITPLRSKRYRTSSSLLNVTAPLVDLDGINFPSQLNSSSSQVFQTPLESSYSAPGILNPSSIFSTKMELPSNQLSESSDDLLGDLLEAQVLVSGQNLKKRDYSTFVEGNDFFEECLGLDNLPQGSVSWSSGLNPKPHSLNLSKSMNDLSTLISGIPSTIQVPEWHNDDIAAQVSNGQSSDAVTADADDTFGVDYNSIASLLPNTTTANSIENPGYVSWDTLPGLC

>Maker00000319 EpMYB2

MRGKGRESERTSFRRQRQTVNTANEAQIIPNVLEKQAQVMGNKAGNEMVVGKRVKKGSSSASLIKGQWTDEEDRKLVKLVNEYGVRKWAQIAENLEGRAGKQCRERWHNHLRPDIKDSWSEEEEKILVETHAKVGNRWAEIAKRIPGRTENAIKNHWNATKRRQNSKRKHKTANQTSNNNARKPPSSILQDYIRSKTLNNNPTIDVESPPQNQFKLVVSDQEPSHESVMSEAFEDELLFMQQFFSESYQNQQVESTLNFYQTDGGHEVYLPEQSPAISGAPMKYHHHSDLFLAQLLNGVGCDSSVCCFDNGKDQNLNMGLQVWDDQQAWYEGKREMDLMELVSSQFSTSTSNRSF

>Maker00000435 EpMYB3

MEAINRCSSSTSSSDSSTSGSSLSRNPDRHDRIKGPWSAEEDQILTRLVDQYGPRNWSLISRYIKGRSGKSCRLRWCNQLSPSVEHRPFSQQEDDTILAAHAQYGNRWATIARLLPGRTDNAVKNHWNSTLKRRVRGQRAAEEGSVAVGCERVLSSPRSAQPCMGLEMDNDPLTVLTLAPPGASGAMANRPPSSENVPAGFWDVMRDVIAREVREYVSSTFSENSGFH

>Maker00001279 EpMYB4

MGRAPCCDKANVKRGPWSPEEDAKLKSYIEQHGTGGNWIALPQKIGLKRCGKSCRLRWLNYLRPNIKHGGFSEEEDHLICSLYGVNKSINVVDSLLLPENSDTQNPYWPHQQMMPVLPPLPYSNQTPSFNDQDSIRRLLIKLGGTFSEHYEGFNFQSPDASSSTQLPVYQVSSASASAINSNQIQFPHSHYYSGLNNLVQGGSTLTQAQEEVSVSYPSALDGLQYLYMINDDDKICPTSSCDTNSMVYPPLIASDFECIPSQEFAFQELRYPGSH

>Maker00001149 EpMYB5

MDPLRSQQRKKEPSRGAWTPEEDRKLTQVIELHGPRRWKFIAAKAGNLSRCGRSCRLRWLNYLRPNIKRGNISDQEEDLILRLHKLLGNRWSLIAGRLPGRTDNEIKNYWNTYLSKKIRPRKQNRGSKTDSKLDITTASNAAAVRNEEQSNPRVVEDSKSGSVEDLDLFIPSCSEGPLNLEWMSKFLEMGEAWFGFADL

>Maker00000202 EpMYB6

MGRSPCCDENGLKKGPWTPEEDEKLVHYIQKHGHGSWRALPKLAGLNRCGKSCRLRWTNYLRPDIKRGKFSQEEEQTILHLHSILGNKDGGGFIFLLASERLLRFLDSCDLQ

>Maker00000176 EpMYB7

MEDSSSSAADDAKTCPRGHWRPSEDEKLRQLVQQYGAQNWNSIAEKLQGRSGKSCRLRWFNQLDPRINRRPFTEEEEERLLAAHRIHGNKWALIARLFPGRTDNAVKNHWHVIMARKQREQSKLCGKRSFQDVYTYNDSNYTSNKITSNSYQDVLHSSRFFDFRSLDHKDRIFADSPSTSLASWSFATSSSNGSPVADSFRREGRDYFNSHSSHFISEISKASDRFHYRINPNSSANYRRVVPSGFGLFGSSDGRIKRDLTGLCDNSTLTKLRASLERGQADHKPIEHKEVPFIDFLGVGVSSS

>Maker00007837 EpMYB8

MARTPCCEKMGLKKGPWTPEEDQILISYIQRYGHGNWRALPKQAGLLRCGKSCRLRWINYLRPDIKRGKFSKEEEETILKLHRILGNRWSAIAARLPGRTDNEIKNFWHTHLKKRLERCEERNVSVSSSCTRAFSSTGGMMPPEQSSAANFGFAGKVSMDADNVSASMTCVATVNPGFQGAHSCDATGERETVGSHCSHKISEEMEFWFNLLMKAGQPSQGHENLG

>Maker00007858 EpMYB9

MGRHSCCYKQKLRKGLWSPEEDERLLNYITKHGHGCWSSVPKLAGLQRCGKSCRLRWINYLRPDLKRGAFSQQEENLIIELHAVLGNRWSQIAAQLPGRTDNEIKNLWNSCLKKKLRQRGIDPNTHKPLSEVENDKDKSPAADKSHQKASMGSNEPSVAEPLKPKPTAMPAERRSLEVSSSFKINNSCTINSNLTSNRPSQDLFLDSSMTSCGPPDIMGYFSFQQLNYAPHAGLSANPNGSLCFMSGSSSSQVMSELNSSIAPAMLNSASSSIFPTTHVSTTVSLQSNNPSISSGDIDGVQNWEASTLGSNGNKSIGRSGSIQLQSSNNFLENSLAWGLADSLKADKDAQVPLLAEQEDIKWSEYLNTPFFLGNTAQNQNCQSIYSEVKPEAGFIIDESSTSWHHTQHQSVFQASDIYSKDLQRFSVAFGQTL

>Maker00006435 EpMYB10

MGRHSCCYKQKLRKGLWSPEEDEKLLRHITKYGHGCWSSVPKQAGLQRCGKSCRLRWINYLRPDLKRGTFSQEEENLIIELHAVLGNRWSQIAAQLPGRTDNEIKNLWNSCLKKKLRQRGIDPVTHKPLSEVENGDPDKDGEKVAAVNSNELNLLKSESSKSDGTSYEHKPCSSNIAPKAYATDMEGSSSTSKINTRIHTNFTTDFSNKDLFLDRFMTTHQESYSTPDLMMNFPLHMTYNNNNNPSTDSSRWFNQTGGRPFDTNTEFTSGAMSTVFPLSTASFLPTSICYKPSLPVPSDDISTASFSMNESQYWEASASTHSNRNSPLNLQTSTVSWGLTDCGAAAKETHQINMMESQTEEAKWAEYFHSNPILMLAAVQNQGPESLCSEIKPASHLVPDTLGAMLPHHKQQDQPLQTSSTIFSKDIHKLTAAFGHI

>Maker00008111 EpMYB11

MDGKMKSESAANQSEDDMCIRKGPWTVEEDTILANYIATHGEGRWNSVARCAAGLNRTGKSCRLRWLNYLRPDVRRGNITLQEQILILDLHSRLGNRWSKIAEHLPGRTDNEIKNYWRTRVIKQAKQFKCEVNSKQFRDAMRYVWIPRMLERIRASSEDKHGSGMEWIKPSYPCSTLNSGWELQEGLKLERSETSSDPIGNGSVTQTWEQGESVENLWNDENIWFLRQQLCD

>Maker00006510 EpMYB12

MDKKPCNSSQDPEVRKGPWTMEEDLILINYIANHGEGVWNSLAKAAGLKRTGKSCRLRWLNYLRPDVRRGNITPEEQLLIMELHAKWGNRWSKIAKHLPGRTDNEIKNYWRTRIQKHIKQAENCQQQHPQLSSETADHQQASSSHVSNNTMADSQPMDTYSPSSFHHQPTLPDHQPFPTHPFPTVLDHSSTCTSHETSYWSMEDLWSNMQILNGD

>Maker00007239 EpMYB13

MDFDPNFQESFSLLSGLFPENPPKPQQLSVSMSHNQPSSQTNNPPLFNTNQNLHHHFHPHLNLNGSNPHSVFLREGPSSSSHIPYSSPTVPFDTSYANESLRMDALHGYHTGRGFWDLSHKLSLPSQALTSQMGPSLPPFFHQAYASPLKPKLQDELLLFFPGNNRWEDDAPSDHRDDDRRKKLQTRKGILKNTNIIKGQWTPEEDRMLVQLVERFGMKKWSQVAKALNGRKEAWTEEEDMILIEAHKEVGNKWAEIARRLPGRTENTIKNHWNATKRRQNAKKQRNKGSTSRPSLLQEYIRQVTSTQATSKQLDIKEEQADVYEAYYNNDVNNVRVRYESASDDEDTSSGDWDVEMYAPEGEEEQEEERMARESSNNGEEELATEMNSLIREEGMKKEMDLMEMICGPGKY

>Maker00007313 EpMYB14

MEFDPNFQEHFPRYSGTFSENSPNLSAPPPSNLPLQSDSFQHDRNLHHFQQLPQLSLNDPNPNPMFVKEGPSSSSLQPNLSFDITYENECSGDFVNTFHQPLDALHGCHIDGSFWAVSKNLPSLSTIPPLYHHIYGPRLWSQDPNSPSFYCTHSHNMVPRQGGAPKNKNIVRAQWTAEEDRLLVQLANQLGMKKWPQVAKAFPGRVGKQCRERWHNHLRPNIRKEAWTEEEDKILIEAHKEIGNKWAEIARRLPGRTENTIKNHWNATKRGLHTKKHRNKGSTSTPSLLKNYIRQVTSIEGPSTQTENIKAEQDVDDEACSNINTTTINNNNIKIRYESTSDDDDTSSGEWEFEALEQEGGAFNNVLRYGVSLIFNACRG

>Maker00007617 EpMYB15

MEYDPNFQDSFFRFCGTFSENPPNPPLISPMLPLHLDSFQNHNFHHFQQQSFNSSMFAKEGLSSSSIPFSSSNIPSESTFGNESFEDFINTYHQPYLVNNNMMDHRTLQCYNMDWSQELPPLAPTSQMVSTTPPIYHQLYYGPHMWNQHDHIVQKHTGNVSNNKNIIKGQWTLEEDRLLVQLVNQLGMKKWSLVANAFTGRVGKQCRERWHNHLRPNIRKEAWTVEEDMILIEAHKEVGNKWAEIARRLPGRTENTIKNHWNATKRGLYSKKHRNNGSAMSSSSPSLLKDYIRQVISLEGTSRQSEDVVKVEQDVGEAASNHINNNSIKIRYESTSDDDDTSSGEWEIIETFEQGVVMP

>Maker00006550 EpMYB16

MGRQPCCDKVGLKRGPWTIEEDHKLMNFILNNGIHCWRMVPKLAGLLRCGKSCRLRWINYLRPDLKRGGFTETEEDQIIQLHSRLGNRWSKIAAHFPGRTDNEIKNHWNTRIKKKLKLLGLDPVTHMPIEQKEVTDDEKLRIQSRLSSSKGSEENVEIIKSFDTSGCTEEASEEQGKKEESNKVTWGDNSNKNEITCSNLDLGSWMNQEKNNSTSSFSSSSFSLEDCSHLCMSESSQLQEKSLLEWVDSISMESILEWDAFNPLDRDLFYFENR

>Maker00006630 EpMYB17

MRSPRVRGKITPCCSKVGIKRGPWTPEEDEVLANFVRKEGEGRWRTLPKRAGLQRCGKSCRLRWMNYLRPSVKRGHIAPDEEDLILRLHRLLGNRWSLIAGRIPGRTDNEIKNYWNTHLSKKLISQGIDPRTHKPLLTLDNNSRSQTAYHESNTSTSNPQSVLEIVHVATSARQIGSCSQQQNQTKAIQNIGNNWGRDSSKVVGLEGGVEVEGERGKIIGEEDTLSSFLDSLINDNEVLGNQQTQVVNHVFPAPPPHSLASSRQFFDLSFWEAEFQSFLEDQHQDPPFK

>Maker00007887 EpMYB18

MSFQHFKNGGGDGFRGAIKQFPPAPLLVSQPSPLSRTFGVAGHLSEIESNKSEIGFQFLSPSFMETRETKESVGEAGEHFGVESNDLSLKLCGEAEAKSSAADKSGYTKLCARGHWRPGEDAKLKELVAQYGPQNWNLIAEHLEGRSGKSCRLRWFNQLDPRINRKVFSEEEEERLLAAHKAYGNKWALIARLFPGRTDNAVKNHWHVIMARRQREQSHVFRRRKPSSENIPKGLKLTLTNNAGSESSISSTRDESASTCTNLSLSPSSAKPPPFFSNPSRQSHGILIDVLRDVGFDKLIRGRNGGRRKGKGVNQSYNSDSKSEVSASESVGTNGIVNLSYSGESENVGEKIDTPFIDFLGVGATA

>Maker00007100 EpMYB19

MGRPPCCEKVGIKKGPWTPEEDIILVSYIQEHGPGNWRSVPTNTGLARCSKSCRLRWTNYLRPGIKRGNFTHHEEEMIIHLQALLGNKWAAIASYLPQRTDNDIKNYWNTHLKKKLKKFQAGLDPHLAANTTTTGEFASKSFNERSSLDPMINHDSSNRLSQSQSSTYASSTENISRLLEGWMRSSPKPDATTLLKENQYHKELQNSNIENGSNMVRSALNSESNLTKNDPEEGGGDIVSHEEFDSILSYENLNNAAWDKSTGDSMPQTHESSKVAAAGDKVRVMAYDERNRQRSENTADPPLSVLEKWLLDENVGQVEEMMELSLTF

>Maker00007775 EpMYB20

MHQFCPRVSSHYPLTLFPTSLFPFIFSLLLPFPFERVVYMALRTLQIRGDVHCDLILINMIINQSMKQEILRKGPWLQEEDEQLTTFVTCFGERRWDFLAKAAGLKRSGKSCRLRWMNYLRPNLKRGPFSVEEEQVIVQLHQQWGNKWSKIARSLPGRTDNEVKNYWRTHSRKKARDEFQYKPDNTEQGAFCERSINDNACKDYSQVNLECQENVTGTKNNAFQDFQSSEWQSTSPPYEIQISDWISTLQKDQSEIEYEIHCNNGTQSSESHPTCFENDCDTWDYSYPASIWDMN

>Maker00007855 EpMYB21

MRVSQTEGGREMGRAPCCDKASVKKGPWSPEEDAQLREYIEKHGTGGNWIALPQKAGLKRCGKSCRLRWLNYLRPNIKHGEFSRDEDRIICCLFASIGSRWSVIASQLPGRTDNDIKNYWNTKLKKKMMGMNVPSAHHQATLLSLLQNSASSSSSSSASSPLSFTDSTNSYCHPPYASFASADQSISFPSSSFQNAHSSCASAGSFYQPQESLMSPVQNYQVKDGMFMFGGEAASCSSSNGNCNNSSQMSHVKEAEAGYGGDHGTLVEQMMMTVNNSNYEYLQGGMAMEESQKVMGCDGDYVSGWTEKQSGLWEETPTLDYGIEEIKQLISTSSCNDFLFDENMTQKRSLYYF

>Maker00006420 EpMYB22

MGRAPCCEKVGLKRGPWTPEEDQILINHVNKYGHSNWRALPKQAGLLRCGKSCRLRWINYLKPDIKRGNFTPEEEDAIIKLHEMLGNRWSAIAAKLPGRTDNEIKNVWHTHLKKRLPQSQQNHLQTTRKPKLKHVLPTVMENPPFSPSQSSSDESTLTTNNDNSIITTASAVANDDDMSININVQSSAPENSLALDDDFWSEVLSADSSGETADIPATGRVESELHFLLSQLDAAQVEGDRDHVSSSMSDGMDFWFDVYTRAEEFMELPQL

>Maker00007206 EpMYB23

MLFLPLHILNGNTFHHFIVTSHPNFASPLHNSLTSFSCISLSLFQNMGRPPCCDKSNVKRGLWTPEEDAKILAYVANHGIGNWTLVPKKAGLNRCGKSCRLRWTNYLRPDLKHDSFTLQEEELIIDLHQAVGSRWSLIAKRLPGRTDNDVKNHWNTKLRKKLMKMGIDPVTHKPVSQVLSDLGSISGLSNVTSETQATSTNLMVSNQQAQEVQSQIIHPENDVQPHHNEAASSCSSSSSSSSSNITRLSSPPQVTTTAAAVLPPCSSFDWSEFLLSDPFMCTEFQQLNQYDIHGVLSSLSPSVIMQNQIQVSNNASDQDSGFVAFEVRMRQLSNMQYCEDTSSSSGDSFVDAILNTDSEIGAAFPQLLDEPIDY

>Maker00006771 EpMYB24

MGRPPCCDKEGVKKGPWTPEEDIVLVSYIQEHGPGNWRAVPTNTGLSRCSKSCRLRWTNYLKPGIKRGNFTDQEEKMIIHLQALLGNRWAAIASYLPQRTDNDIKNYWNTHLKKKLKKLQPDDQGNFGDGSSVSQSMLRGQWERRLQTDIHLAKRALSEALSPDQTPSGISESNPSKPATLGLSYASSADNIARLLKGWMKNPPKACTSSAMTRSSINNLAASASGNDTASSKGISTIASKGNSVQLSDTSSESLAGFEFLDCSSNSDFSPEAILESKPDSTVAEMPLYLLEKWLLDDAATITSFEDADAHFV

>Maker00007632 EpMYB25

MGRAPCCDKANVKRGPWSPEEDATLKNYLQKHGTGGNWIALPKKAGLKRCGKSCRLRWLNYLRPHIRLGGFTDDEDKLIFTLYATLGTAQLPGRTDNDVKNHWNTKLKKKFLAEKSCNGAISSNMSEGTNFVPFSYLPPQTPQGDAFVLDHKDWEYFEPCVLELEQNTATISGSKPQKFEGSDVGSCLMSESCSIPYSVQVSSSSGSLEEKNDHHAKWFGFKLEDTSMSDLVDDDLFSGFAPYDQILINESFPNQ

>Maker00007894 EpMYB26

MSTSKSESSCSEDDNELRRGPWTLEEDTLLTQYISTHGEGRWNLLAKHSGLKRTGKSCRLRWLNYLNPEVKRGNLSPEEQILILELHSKWGNRWSKIAQRLPGRTDNEIKNYWRTRIQKQARHLNIDTSSTEFREIVGRFWLPRLMQKARETASSAAMSIQNQTIPLPFHSVSQCSQIATVPTQIPWQGPCVMNETVQTTSCMSSSSSESSNILEMSQFQALENTDMSSFMYDGYYVNNNTYNMETFNLGTTVTAEDMQSNWVSNDVACSMWDMDELWQFRNLQE

>Maker00008171 EpMYB27

MAPRADECDDDELSSSDDDDDLKEDMEALARACIIAGTNPDDLKTTSAVAPDDENADAVVPKSQDPLLGPGDALVPLADSDDEQSDIQYLERVQERYQPSSLKPLRVLTALSLSDDDEDDMETLRAIMRRFSAYGRGPGEDNVDKMAEGGHASSLGFEEEMDNSMTYKESDPNDSFPVSQDAINATNFLAGDDEKQPCKNNERCKPDACKLSNLPDRRSKFPLSAQGFIDAIKKNRSFQKLLRSKLIQMEAKIEENKSLRERVKLLKDFQVSCIRRTGQAISLKKDPRILLISTKRSPASSNSKINDKKKSPMYYGPAENAHVAKYRMVLKQFPLLLDRKKWSSVERENLAKGFKQQFQETVLQMSVNQVSSEGSFGDANDFCNMHESIKDLEILPENITEFLPKVDWDRLASMYVVGRTGAACEASSLTIVSCLCPFYEEYLSPQALQSSMTIPSQTVYHFWVSIRVQDQHRSCRWLNCDHPLINHSPWASEEDKLLLHIVQETGNRNWFKIAALLNTNRTPFQCLARYQRGLNPSMLNSEWTEEEDVQLCSAVAIFGVSDWQSVASVLERRTGPQCSNRWKKALSPDRKGSFTLEEDERLTVAVHLFGRKWNQIAKFVPGRTQAQCRDRYVNSLDPSLKWGGWTEEEDSRLKAAIGRHGFCWSKVAADMPPRTDSQCRKRWRVLCPDQVPMLQAARKMQKSTLISNFVDRESERPCLSLNDFLPLALLGPPCDPDDVKSPRKRKKKSSNTSKKMRPKKRTKKASSCLKEVKGDKVEPHNGNDVHQPMITEPYLEDNSVETVLTTYHQEDLGGHNNTDRIIESNSTWKGSNELLEESHTCIIPCENPEAGKSDGHCRKIASMDSSKHQIGRKSMLSASISDGVAVGGKVTRNSTSKKGRKKIGKQSTIVKSGVTAEDHLVKNKMPKPGRLSSMQILESQDEDNMTLATFLHNLSNKRFKNTNQLTQDAFSTSSCSDSVLVFDQNFATHGGVRNSQGVKILTDHDGDTAVTCFPVNESQESTPAAKQTRGCLSSSLKKGFGLLPKEGHNDHDRNQVMSVMQDMVSDNGCTDVTPGTLSFQNHSHNSKEMSAIKVDDNGKAMPRSVEEQTMVLDGVTEPKNINAGEDESTDITLASLLKKKESKGKAMVGPVDEQTIALNGVTEPKSINVGEDESFDVILASFLKRKRRQGRTAKRRL

>Maker00006745 EpMYB28

MRKPCSDKPDSQKRSWSKEEDQKLIDYVQTHGEGCWPSLPQAAGLRRCGKSCRLRWLNYLKPDIKRGHFGEDEEDLIIRLHALLGNRWSLIAGRLPGRTDNEVKNHWNTHIRKKLIKMGHDPNNLHRLRGSSPGTSNSINESNHRRKDNETANDQVNYLHADNIHRHHHQVFESTSQPQFNLNAFPDLNLDLTLAIN

>Maker00008181 EpMYB29

MCSRGHWRPAEDEKLRELVERYGPHNWNAIAEKLRGRSGKSCRLRWFNQLDPRINRTPFTEEEEERLLASHRIHGNRWAVIARLFPGRTDNAVKNHWHVMMARIRRERSKLYAKRPHPHQCSPQPDPDDQDHHIDYDTRSSTNNSLLLPSLVEKYYDKQAYPYCLSYNNPFLQFPNKFHFQHPTSCSPAMLHDRNQSVEFYDFLQVNTDSNRSDVTDNARREDEEVNQDGRQNKDGVPFIDFLSVHGSS

>Maker00024486 EpMYB30

MGRSPCCDKVGLKKGPWTPEEDQKLLAYIEEHGHGSWRALPAKAGLQRCGKSCRLRWTNYLRPDIKRGKFSLQEEQTIIQLHALLGNRWSAIATHLAKRTDNEIKNYWNTHLKKRLAKMGIDPVTHKPKNDALVSRDGSHSKCAANLSHMAQWESARLEAEARLARESKLRASQSFTSSAHPVLKAWNNVGWLKPTDQSINSGTGDLESPKSTLSLSENTPPIMTMIEFVGTSGSCEAEGMVKDEGDQREWNHGNGMENSMSYTSSLHHESTISNMEGAWTPQEPPLMTGTSGDTVAEDEGFTNLLLNTNPDHRSLSEEGGAESNNWESSGGGSGSGMLCEDNKNYWNSILNLVNSDSPSDSPMF

>Maker00019416 EpMYB31

MGRSPCCEKAHTNKGAWTKEEDQRLIDYIRLHGEGSWRSLPKAAGLLRCGKSCRLRWINYLRPDLKRGNFTQEEDDLIIKLHSLLGNKWSLIAARLPGRTDNEIKNYWNTHIKRKLISRGINPQTHRPLNETTTSVDSAAEWHLKTSPAGGNDVVAKKQKQPSDAFNCIDFSSVKAIEDLNNTRSGTTTEEEPSSLHRDSSLHGAGEPNLELSIGLEPFQSESIRAAQGNSAESKLRSVQPPVTAAQTVACLQWQFDFQRWGSV

>Maker00019487 EpMYB32

MMERTALKIDESDGLKKGPWTAEEDRILVDHIEKHGHGSWRALPKLAGLNRCGKSCRLRWTNYLRPDIKRGNISEQEQQLIINLHAVLGNKWSTIAAHLPGRTDNEIKNLWNTHLKKKLLQMGLDPVTHRPRSQHLNLLSNLQHLLSALTTMNPPHNNPWDILHTIQTLTPSQLAKLHLIYNVLQVLATAAAAASFAPSNLDPLSHPSMMDHLLFQDHQTIGNFLSQYSNLPNVEDLLHHHRQQAPQSVLSDHQLHKMMPCFAALTSTSSLLPNLVLSTPECSPTLNEVKNPSERFDINDPSSTSTAYNALEDFMDEQANEAYWKDLME

>Maker00014356 EpMYB33

MEREFVRGTYIKKGPWSAEEDEILLKHVKKNGARDWSSIRSKGLLPRTGKSCRLRWVNKLRPNLKTGCKFSAEEERMVIELQAQFGNKWAKIATYLEGRTDNDVKNFWSSRRKRLERMLQKPSPSKTLRNKAKAPIHQPLVEEQDREFQVPSFSSNHLEENPSGIEELKMMNPPKFQNLEAVEAVPLHMVPSFESSSQYTFSQVIPESHQVDFGLLPVCDGLADPEHFDAAFLDVFEQQKVLECACSSKKVESRLPGLALGGIAQSSSPANGFFEDFPSEIFDYFEDLPNSSNARFGSSGQMAVM

>Maker00014151 EpMYB34

MSQMTHDIEDGELPNDQTGPQLNDEGNGGSANGILLKKGPWTSAEDAILVDYVKKNGEGNWNAVQKHSGLSRCGKSCRLRWANHLRPNLKKGAFTAEEERLIAELHAKMGNKWARMAAHLPGRTDNEIKNYWNTRIKRRQRAGLPLYPPEVCLQDLQESQQGQSTVGKNGDGGHNDFLQENSYQIQDPVFDSLSANQGGLPYVPELPDISANNILLKGFGSSQHCNFLPSTLSFHKRLRQSEMPFLSSTDMNRNGFYPLHQIEDNSYDKIAQSFGYHSPLDPGPSSQNSLCYSHSLSNGNSSTSKPTIEAVKLELPSLQYPETDLGSWGTSPPPPLLDSVDIFVGTPQPTSLLESDSSSPYNSGLLDALLYQSKNLSSTQNHCSDKSSNSSTATPGDRADSSTLHMYETECEDYADCVSPFGATSILNECLTVGAKGNSVDELQSAQTVNGNVMKSESIYLDLTPNSEDQDLSMLNIPRPDVLLASDWFELGSAFGKNQADTNNSIATLLGDDLATDYDHISAEISSPNQVWGLDSCTWNNMPAVCQMSDLP

>Maker00003938 EpMYB35

MTKPSSNNDQKNGLKKGPWTPEEDQKLVDYVQKHGHGKWRILPKNAGLKRCGKSCRLRWTNYLRPDIKRGRFSFEEEETIIQLHSVLGNKWSTIAASLPGRTDNEIKNYWNTHIKKRLLKMGIDPVTHTPCLDIQQLSSILTSSLYNLPHLDYPLFGTGSNVMNPSLLSLLTAFLSSSQNSNALNHNFHQNQLNNPIQAFPIQENPSCAASQPLNLESQLMKSKLDQISSDTADFCYQNSLPDLGQYDGAHYSSNLLETQIALNNKGVPNFTLGSLLSYSTSPSSSTSPSTLNSSASSTTFVNGTTEEERDTYCSNMLIYNISNGLNIDDSGLL

>Maker00003683 EpMYB36

MGAFAIKTEFAFANRSNKVSLSNFRSTVAIPSLLQENTFQLRLSFWRLSTSILSASTMGRAPCCDKNGLKKGPWTPEEDLKLINYIQAHGPGNWRTLPNNAGLQRCGKSCRLRWTNYLRPDIKRGRFSFEEEEAIIQLHSIMGNKWSAIAARLPGRTDNEIKNYWNTHIRKRLLRIGIDPVTHAPRLDLLEISSVLRSALTNQSLLNLQSLLGAQALMNPEFFKLATTLLALKNESPDLVSQNLQQTVAENHQADSQVTPFNQFQIQTQTTNVEDLSGNVTSMSCSSWLENSVPSYLCENILNPQQNQGDLRGNPAFGQRLINNENIQNMGHKSVLSTPLSSPTPLNSSSTYLNSNAEEEKDTHCSDLFKFEIPENLDISDFL

>Maker00003971 EpMYB37

MVRSPYYDKNGVKKGAWSREEDDQLRAYVLRYGHNNWRQLPKLAGLARCGKSCRLRWMNYLRPDLKHGNFTPEEEELVIRLHHQLGNKWSLIAEKLPGRTDNDIKNHWHSHLKKTLNRNDSKYLKTSEYTESESPCEFVGADSESHHILESSMARPISSETSYIQENHSSFAQPTIQSDTSEELSSDFWSRPFLVEDTSGYDIAPMFWFPYEGTDVSDLQGLLDW

>Maker00003854 EpMYB38

MVRTPSFDKSGLKKGTWTPEEDRKLIAYITRYGCWNWRQLPRFAGLARCGKSCRLRWMNYLRPNIKRGNYTPEEEELVIKMHKKLGNRWSAIAAHLPGRTDNEIKNHWHTTLKKRLQSNPSSSLTDENRPKKSKPTSTSTSTSTPTPLITSGSPPSSGAPHHTILDSTPLSPQSFVTATSSVNSCLEDDLFFLDAFSEVDTQHFLTDPHTDRLFSAQSETMPILSPPRAPSVPHAPLVDDDDFALLDSLSQAAIFENSLIETQVADMCWQCCPQTSLLAVELEYLHPLYDLDLWSQNNLCV

>Maker00004028 EpMYB39

MVRTPSCDKSGLKKGTWTPEEDRKLIAYVTRYGCWNWRQLPKYAGLARCGKSCRLRWLNYLRPNIRRGNFTPEEEDTIIKLHHELGNRWSAIAAKLPGRTDNDIKNYWHTNLKKRVEDKSGTQQKSREESQVEDKEYSAELPVKNDIGSCDHHHQMEDSCLLSPQPSTSMITMELETTAASNALSLIVEDDQFAFLDAYADVNFWTDPYMGDIFCDPTEVGSLPPSLTQSDYFTTDPSCDVHLWTTTNVYDQSIGLF

>Maker00003767 EpMYB40

MKKGPWTAAEDAMLIEYVKKHGEGNWNMVHKNSGLARCGKSCRLRWTNHLRPNLKKGSLSPYEQNIIIHLQSKIGNKWARMASLLPGRTDNEIKNFWNTRLKRCLKAGMPLHHHHPPPSQPSNAFASHFISAPPSLSSPPHLPFFSAPNLFHSHTNPFHLLYTSNIPANLALVTSLGSSSSSYTLHVPVESLPFVNGYTTAPSTPASGFTHDDCCEVAPLILSQPNNGLLEDLVVEAYALSRNDDSSSTTLSDLSNRRKHTTTETQGDDLLSFPQSTGKKPRSGEDPMGKDDDLLGLLASFSSEFPMSDWFGGESQSLGFECQSNTSQGLTGQECLPWALGNCRNNLSGTC

>Maker00003993 EpMYB41

MGRAPCCDKENVKKGPWSPEEDSKLKEYIEKHGTGGNWIALPLKAGLKRCGKSCRLRWLNYLRPNIKHGEFSDEEDRIICALYANLGSRWSIIAAQLPGRTDNDIKNYWNTKLKKKFMCMLPHPHHSKPSFHSSHHDLSFPSSQPLSSSMYTDCSSLSSTILDPPYVPSNYKGRAFPSFQNQESIFLSGCAMQYYGMRDNLIMSGSEGSCSQVHSRVVKDTMSIQNSMPSGYEEYSNKFMLSSSSNNEQGAEKSTEYFGETPMETPMDNYRIEDIKQLINCSSSNSNDGYSGYYFNIDE

>Maker00004096 EpMYB42

MVKQEFRKGPWTEQEDVKLVSFVGLFGDRRWDFIAKVSGLNRTGKSCRLRWVNYLHPGLKRGKMTPQEERLVLELQAKWGNRWSRIARKLPGRTDNEIKNYWRTYMRKKAQETKWAATAPSTSSCHSSLSSNNHTVGSHPSKESGEESFYDTGGPNVIDSTKIKIGKEQEGEQGYSMDDIWKDITLSEVNIQHPVYAGHIEEECNFFCPDLPSPSWEYSSDSLWVIDEKESKMLLPTSDQFISSFERGRAFLTG

>Maker00017936 EpMYB43

MGRAPCCDKNGLKKGPWTTEEDQKLVDYIKKHGYGNWRTLPKNAGLQRCGKSCRLRWTNYLRPDIKRGRFSLEEEETIIQLHSILGNKWSAIASRLPGRTDNEIKNYWNTHIRKRLLRMGIDPVTHSPRLDLLDLSSLLYASSSSSQLNNDLSSLQFSMNPPLLNPHLLKLASSLLSAQRRDHINNNHSHNHQTLSFCDQIPDTSLFQEMPIPEAQSTMVQPNVDPYISSNFTDFCDWQSNTDGISSSTATTTLTQDYVPEVLLPSFQYCNSDEYQNLMDPQSNNNFSLASVRSTPSNSNSTYINGGCSSTEDERESYHSSNMFKFEIPELLDVIDFM

>Maker00017704 EpMYB44

MYRSVPPLTALDSFLWGQQSRNHNITAWPHCTEGSNFIDGLLLANEQAFNWAHTQVPALMCLRKQQEHDDDDVVVVRRRIKKRGPCVSLIKGQWTDEEDRKLVRLVKQYGVRKWAEIAENLDGRAGKQCRERWHNHLRPDIKVVFSFSPQSLSLGSMFSCSIADSIPCSDLSKDSWSEEEERILVETHAKVGNRWAEIAKSIPGRTENAVKNHWNATKRRQNFKRKKKNTHTIIRKPRSSVLEDYIRSKILISDAVQNTAENQSEPSSESAVLNGESVITEPYDDELLVLQRFLSDIQNQKQSLADSQHSENSPTSYSLDFCQTHDEQHLITDATTEPRFVYSISNQDKKINLYESLLAEPIIKHSESDIYLSHLLNGPPSSSLLCGYGSQNLKVDLHMGYQDGSESKREMDLMELVSSSKSSTSIDNSI

>Maker00017238 EpMYB45

MGRSPCCDKVGLKKGPWTPEEDQKLLAYIEEHGHGSWRALPAKAGLQRCGKSCRLRWTNYLRPDIKRGKFSLQEEQTIIQLHALLGNRWSAIATHLPKRTDNEIKNYWNTHLKKRLAKMGIDPVTHKPKNDALLSIDSQSNKSAANLSHMAQWESARLEAEARLVRESKLRSHSLQHQLCNSGFSSSSSATTPALVASKSEAPPISYRSLDVMKAWNGGWLKSTEGGGDLESPSSSLTFSENAPPIMTSGLGESTMPTIEFVGTSGSSETGIVKEEGEQEWKGYESSVHLPEYKDGMDNSLSFASSLHELTMSMEGTWTSESLRTSTDAIAEEGFTDLLLNSNSDDRSLSEEGGGESNNCDGNGGSGSDFYEDNKNYWNSILNLVNSSPSDSPMF

>Maker00017014 EpMYB46

MGRPPCCDKEGVKKGPWTPEEDIILVSYIQQHGPGNWKAVPTNTGLSRCSKSCRLRWTNYLRPGIKRGNFTDQEEKMIIHLQALLGNRWAAIASYLPQRTDNDIKNYWNTHLKKKLKKLQTGLEEDSARDGFSASHSMPRGQWERRLQTDIQMAKKALREALSPHRPSCSSQSNPTNIGTFSSTCAKPTQSLCYASTADNIARLLKEWKKNPPKRNPSRTNSVVTHNSFENLVACSKEAPSDEARKGSSFEQCKMLESLFGLESLDSSNSEFSQSLSPDPEPNTLFQDESKPEFGAELPLSFLEKWLLDEVALPGIS

>Maker00016816 EpMYB47

MSSRSFNNGGDGSGRDVHLLHPVPSYASPFLVQTPHLSRSNTSGQIDDEMGFQILSPSFRPHGNRGTTRCDGEAGGHSGAQNKSLALKLGEEDMEAKSSYGIRNGHMKLCARGHWRPAEDAKLKELVAQYGPQNWNLIADHLEGRSGKSCRLRWFNQLDSRINRRAFSEEEEERLLTTHRMYGNKWATIAKLFPGRTDNAVKNHWHVIMARKHREQSSGCRRRRSSSEIRREALNLTLSSNAVSESTNSSNMIDESASSSTDLSLSPTFFSDPSPQGQGSMMGVCNDAKLRTGDVGFDRFIVGWKGWDEGGGYIGKVVDQSNSDSNSEISVSESVATNRTNLSISGESENVGVNINTPFIDFLGVGAL

>Maker00018290 EpMYB48

MGRPPCCDKVGIKKGPWTPEEDIILVSYIQEHGPGNWRSVPTNTGLSRCSKSCRLRWTNYLRPGIKRGSFTPHEEGMIIHLQALLGNKWAAIASYLPQRTDNDIKNYWNTHLKKKLKKFQAALDPHLIVSSDSTTSAQFVSKSFHDRTSIDMSNTSSFRFCQPQSQSHSSSSSSTYASSTENISRLLQGWMRSSPKPDTGLLKGTKDKELQDNNINSFQNSKPMVKSASVPAALNCVSYHHPTKDEQEVGDMVSHEEFDSILSFENLNNAAWDKSTCDSVPDKISEAAAEKVHDKQRSDSSSAPPLSFLEKWLFEENAGHEEMMELSPMF

>Maker00018431 EpMYB49

MGRAPCCDKVNVKKGPWSPEEDETLKAYIEKHGTGGNWIALPQKAGLKRCGKSCRLRWLNYLRPDIKHGQFSDDEDRIICTLFSSFGSRWSIIAAHLPGRTDNDIKNHWNTKLKKKVLMNPSAQGNKPRQSTPLTILQNSTPSSSLSFPDSTNCYTGHADLSVSGSSSLLSGNISASAANSIFQKQDGSFLGPIQNQQVNMVGGEAASCSSSDGSYNNQMLHVKEAEFVYGRDGTLIQQVGGAHDFFNNGLWGEDLLDYGIDEIKQLVSTDSCSNFFFG

>Maker00017717 EpMYB50

MGRAPCCERMGLKKGPWSTEEDQILINHIQSHGHGNWRALPKQAGLSRCGKSCRLRWTNYLRPDIKRGNFTSQEEHTIIHLHHILGNRWSAIAARLPGRTDNEIKNVWHTHLKKRLPLNQQNLPTNPNPIPKKQHPVTASTHHHVHPVVIKAESSDYCMISTPPSPPQCSVSDTSTLTTSDHPERNSNDVSTNDDVQTPTHTIETLDDDFWSEVLSADSSGERGEFAAVGACADPHFEFPFSPLTVTGVSSMCDGLSIDFWDDNAYTRPQELTGLLPQL

>Maker00018152 EpMYB51

MGRAPCCEKVGLKKGRWSAQEDEILLQYIQVNGEGSWRALPKNAGLLRCGKSCRLRWINYLRADLKRGNISVQEEELIIKLHSSFGNRWSLIASHLPGRTDNEIKNYWNSHLSRKIYAFKRMASTDQTQPAATTDTNKKRRRGRTSRWAMKKNRSNTQKATEKTNQNSVEVPVPPTPALENEGLSITSAVDQLEDFTVSEGGRVESETETVQECRESIIDGGGVLGFNDMVDGWLQEEGGGGVLGLSQERGRNDKVSCFDYRMTAAAEEPETQASNTQHWYSCSPMLSVSEYDYWDNWEIVVDEHLIQNESSSWEDKQNLLSCLWEDDDWENDRQIPG

>Maker00017944 EpMYB52

MDQDQVIDYCNMNIKFEMVRSSSSDCGSSSGTCGSEEEMGIRKGPWTEEEDAILANYISLHGEGHWNSLARSAGLRRTGKSCRLRWLNYLRPNVRRGNITLQEQLLILELHSRWGNRWSKIAQELPGRTDNEIKNYWRTRVVKQAKQLKCDVNSKQFRDALRFVWIPRLMERIQTQSSHSPLLPVEPTSLYSHSTTSTVDEDACSAPAAAPTSGDVFDYSRDFQAFEAPNADFWTYENICFLQQQLADDS

>Maker00017541 EpMYB53

MEMKVKGGCKSRSQPCEDEMGVRKGPWTVEEDTILVNYIATHGEGRWNSVARCAGLRRTGKSCRLRWLNYLRPDVRRGNITLQEQIMILDLHSRWGNRWSKIAQHLPGRTDNEIKNYWRTRVMKQAKQLKCDVNSKQFRDTMRYVWMPRLLERIRTAASEYNPTTPSNPNHSSPTPLGFNPSFSPAQHSAHHVSGSYQNGSDVGLESEQGTGGEWVETLWNDDNIWFLQQQLLEDL

>Maker00018158 EpMYB54

MGRHSCCYKQKLRKGLWSPEEDEKLLRHITKYGHGCWSSVPKQAGLQRCGKSCRLRWINYLRPDLKRGTFSPEEENLIIELHAVLGNRWSQIAAHLPGRTDNEIKNLWNSCLKKKLRQRGIDPVTHKPISEVQNGDDKDKVLNELKPLKSESPNSDATKVYTHDMEGCSSFKFNNNTGNTNLMSSCGSKELLPDGFMTSYATSDLMGNFPLQMNYASTDDTLPTNSVPSQWFTQTGRPLFNMNSEFASNSMSTILAPPATSYLPSVGVPCEDICATSYPENAAQSHFWGLTDSSKETQIQVLETQTAEARWSDYVQNPILMLPVTHLISDNLGAMLPHNKQQDEPSQCCNMFSKDIQQLTAAFGHI

>Maker00017835 EpMYB55

MGRHSCCYKQKLRKGLWSPEEDEKLLNYITKHGHGCWSSVPKLAGLQRCGKSCRLRWINYLRPDLKRGAFSQQEENLIIELHAALGNRWSQIAAQLPGRTDNEIKNLWNSCLKKKLRQRGIDPNTHKPLSEVESDKDKPSSADRTNQKASTSNEHTWASESAKPKPLQVSSASNISNSYDLFLDRSSSDMVGYFSFHHFSCYTPNPDTNTSLCFIPSSTSSSQIMSDLNSTVTPTMLHSVLSTHVSPTLTFQSDNNPSPHIHGVQAWETTALNGHGGKHSGINSDIQLQNSNDFIENNTLTWEKDTHGNGVPLQAEQEDMKWSEYLNTPFLLGNQISQSIYSEVKPETGFTTDEASGGWHHIQHPQVFQASDIYTKDMQRFSVAFGQTL

>Maker00017689 EpMYB56

MRIMIKGGVWKNTEDEILKAAVMKYGKNQWARISSLLVRKSAKQCKARWYEWLDPSIKKTEWTREEDEKLLHLAKLMPTQWRTIAPIVGRTPSQCLERYEKLLDAACVKDENYEPGDDPPGNCALIDPNPESKPARPDPVDMDEDEKEMLSEARARLANTKGKKAKRKAREKQLEEARRLASLQKKRELKAAGIDIRQRRRKRKGIDYNAEIPFEKRPPPGFFDVTDEDRPVEQPKFPTTIEELEGKRRIDVEVQLRKQDMAKNKIAQRQDAPSAILHANKLNDPETVRKRSKLMLPPPQISDHELEEIAKMGYASDLVGNEEITEGSGATRALLANYAQTPSQGMTPLRTPQRTPGGKGDAIMMEAENLARLRESQTPLLGGDNPELHPSDFSGVTPKKKEIQTPNPMLTPSATPGGAGLTPRSGMTPARDGFGFGMTPKGTPLRDELHINEDMDMHESTKLELHRQADLRRSLRTGLSSLPQPKNEYQIVMQPVPEDVEEPEEKIEEDMSDRIAREKAEEEAQLQALLRKRSKVLQRELPRPPAASLELIRNSLIIADGDKSSFLIRRGKKGVKRAANGSVVVPLIEDFEEEEMKDADQLIKEEAQYLRVAMGHESEPLDEFVEAHRTCLNDLMYFPTRNAYGLSSVAGNMEKLAALQNEFENVRKKLDDDKEKVVRLEKKVIVLTQGYEMRAKKSLWPQIEATFKQMDTAATELECFRVLQKQEQLAASHRINNIWAEVEKQKELEKNLQKRYGDLLAELERIQSIMNQYRAHSQQEEEIAAKKRSLELAEIESDKASMQGLENHDSAPRADEHGSALLVNPTEDETVDQQVDLMQAQAMSGPKNDMDVDADKEQMTHETDVKLEDATPVAEGASATVVPVKSTDSCMGHRETITGVPEDTRSIEGCREDQDFKNPENAAEAENSRDDKVLETTALD

>Maker00009014 EpMYB57

MDVIARAPNSPALQTDDLDDLDLRRGPWTVDEDLTLINYITIHGEGRWNSLARSAGLKRTGKSCRLRWLNYLRPDVRRGNITLEEQLLILELHSRWGNRWSKIAQYLPGRTDNEIKNYWRTRVQKHAKQLKCDVNSKQFKDTMRYLWMPRLVERIQAAAATAATATTTASSQTLSTTTSVTTNTTYNQYSNSNSLNSNMENMLLIPAINNDFGGSQVSQSYTPENSSTGASSDSYGTQVSTMSELTDYYSTVPDQEYYQPSQISYSDCITSPSGFFPQAMDCSQVMEGSNPWIHGGDTLESLWNVENMLFLQQQLNIDSM

>Maker00008746 EpMYB58

MGKSPCCDEIGLKKGPWTPEEDQKLVSYIQEHGHRSWRAVPKLAGLNRCGKSCRLRWTNYLRPDIKRGKFSQEEQQTILNLHAILGNKWSAIASQLPGRTDNEIKNFWNTRLKKKLIQMGYDPMTHQPRTEFFSSLPYLLALANLKGLLDHQPWQHSVASRPQAEAAQHARLQGPQHLHSQLQPSANLNSNFTDMDAININLLKSLCLSNKNTVLSSTQLENNINNLSPTRVNNLQLDDDLFSNMPDSKSPCDYHQPPVAVIQGESYPNSPHLLSISSSISSPSTVPEILLTESVRYGEAGPSMWPDLLMEDSLDSLFPEISYSA

>Maker00008663 EpMYB59

MPRSSSCETKSCNRGHWRPAEDEKLKQLVEQYGPQNWNFIAEHLDGRSGKSCRLRWYNQLDPNIVKRPFTEEEEEILLALHKVKGNKWAAISRFLPGRTDNAVKNHFHVLMARRKRERFPLFGDRSSIHTHNINHHHHHIPILGSATYRPTSGINFTFQSNRNMSIFNVSSSHSLLLNPPWNTQSGTTSSITSELVSFEASATANKPRNDHSSASTPASFLLSSYNSFTAPGIPSIGKIVPLPHWPSKSHYGQRTEDYSMKSMTGKTIMEEQVEKLKGVSFIDFLGVGSCSSRDCGS

>Maker00008794 EpMYB60

MGRSPCCEKEHTNKGAWTKEEDQRLINYIKLHGEGCWRSLPKAAGLLRCGKSCRLRWINYLRPDLKRGNFTEEEDELIINLHSLLGNKWSLIAARLPGRTDNEIKNYWNTHIKRKLYSRGIDPQTHRPLSAAAVPNTASARDTATVVRSNSDSSNNNNSKFQLVTELGTANSMNATKMVGTESACEDSNSSSGVTTDEAIYAHTQLNLNLSIGLPCDQPPPVNQGGLKRQPQEQQPEGQVLHNWYGNFISTGSQGVCLCCGLGLQSKGQACCCKSAMGAVAAGVTTTASPPAVTENTDNLYRFYSPLNI

>Maker00008847 EpMYB61

MGRHSCCVKQKLRKGLWSPEEDEKLFNHITRFGVGCWSSVPKLAGLQRCGKSCRLRWINYLRPDLKRGMFSQQEEDIILSLHEVLGNRWAQIAAQLPGRTDNEIKNFWNSCLKKKLLKQGIDPATHKPFTQPQIKQENNTTEAPSLSVHNMHLSNLASSQSSQGMPFLISDSSYYDAAMLTEGSRDDIFTTMSKPEWVDPLCYNESSSSQYQYGTGSNYGICSMPSLTSSEHNMSVITELSDNIKSASKMSSSNSSNMSSYMMENVGLLSLEGENKMEYPWFQFQGINGIKSDELMMNTCSWQEAVQLQNHNSIDFNSYPLTSFFSQDLTAASYDVFRHI

>Maker00008587 EpMYB62

MVKPPYCEKLSVKRGVWTPEEDAKILAFVSKHGSGNWTSLPKKAGLKRCGKSCRLRWTNYLRPDLKHDSFTPQEEDLILKLHAAIGSRWSIIAQQLPGRTDNDVKNYWNTKLKKKLTDMGIDPITHKPFSKLLADYGNIGGIQKPSTRIGSLSKDLKNAVLFKSDPFPKLSSTSPKLEPPTESSFLSNRDMMEKETVSAAILGEGFLSSISSPSNSSSCSNAAQEGLSLPFCWNDFLLEDVFPPAADNPENENVILSENKEEPFTYGLIGDEESSVKMVAISLYRGNLHRVPDVPRRWLMPAPKISLKDFKSLLARRSKALSRLLDDGAITTTTTFSNPSHASLSKGQNEVPAIDKPSLAIQPESVKVIDKEEGPSWEGGVHQDSELFNSFINKPIDGFDSLTNSKTDVSEKASELVDGDATLVTKQPETIGDKVDMLNDQEKRKKEVEDKLQVLNAKKHNLVLILKQILNTEEELKRRNSVQAIALRPSVPFQTEGTNDTGLLSRHTTPRLGSEANLGGDVEGSETDDPSNHNMHYRNVHWMNNMSPSESPLMRTPSVHQNMVSQPSLRTSVGVTESPSRFALSHQGNAMNPASVSVSGTGYMASSPSPAASGGTSVFRDARHPSPWN

>Maker00021281 EpMYB63

MFVMGRPPCCDKIGIKKGPWTPEEDIILVSYIQEHGPGNWRSVPTNTGLMRCSKSCRLRWTNYLRPGIKRGNFTDHEEKMIIHLQALLGNRWAAIASYLPERTDNDIKNYWNTHLKKKLKRLQTGTEGDDQEDSSCSQSKGQWERRLQTDIHMAKQALCEALSLDKPAEMDTKPSNHTIQTSPYASSTDNIARLLQNWMKKSPAETSSPGGCLSNMVTTGSSSSSEAATQNNSTNNNALDSLWSFNSDASQQSVSVDDTTNYNNSMFQDESKPSLESQLPLTLIETWLFDDGTTQNHQDLINESLMEETTPMLF

>Maker00021702 EpMYB64

MADILRSQKIEFLMPVTLVTILTPEVGYWAPSKALHFRNFSHPMERKGLALKPQAKKNLWKPEEDLVLKTYIEAHGEGNWEAVSKNSGLNRGGKSCRLRWKNYLRPDIKRGGMSEEEEDLIIRLHKLLGNRWSLIAGRIPGRTDNEVKNYWNTHLNKRCPRGKRKAIDSNKDHQETSKKLGTSNSDFPISASIPETGLLDERKQESSNITNSWVHETQSFDCDLDFPIVPGSSVEFGFDDESFMKNLDSIVLFETEALDYGWLESNSHTQCFMP

>Maker00021607 EpMYB65

MGRAPCCDKANVKKGPWSPEEDAILKTYIDKHGTGGNWIALPQKIGLKRCGKSCRLRWLNYLRPNIKHGGFTEEEDNIISSLYISIGSRWSIIAAQLPGRTDNDIKNYWNTRLKKKLLGRRKKENIDKDNSNISDHTLSNSALERLQLHLQLQNPNFSFYNNPALLPKLRPFQQKMIHNTLIQSLNHTSNFATTPQKYSELCKPASANNTDAGQRDSVKISSPGSSGPVTGDGTNSVDTGRTEFDMGIDQVSVLQTELDDILMSGYKTQQQKDQTGEYDCFGEIMSGFSTDNLNWWSSDFDAKSASSNSWDSTNVASTPHILQPEDQRLFQDYELGYIS

>Maker00012299 EpMYB66

MGWSIIEEEGWRKGPWTAEEDRLLIEYVRMHGEGRWNSVSRLAGLKRNGKSCRLRWVNYLRPDLKRGQITPQEESIILDLHARWGNRWSTIARSLPGRTDNEIKNYWRTHFKKKVRSPTNTVEKAKNCLLKRQQFQQQQQRQQQQLQINLDMKGIMALFEDNNTNEYKVPSSLCSMASMNLNTLEEQGCFYYSMINNNNNNAAASEASNEDIFWEGLWNLDDIHGNFNATNATTKPNM

>Maker00003290 EpMYB67

MGRSPFSDDISGLKKGPWTPQEDHKLTHYIQKHGHGSWRALPKLAGLNRCGKSCRLRWTNYLRPDIKRGKFSQEEEQTILNLHAVLGNKWSAIARHLPGRTDNEIKNFWNTHLKKKLIQMGYDPMTHQPRTDIFSSLSHLLALANLKQLFDHNSSSLQFLQHLLQPEARTASYQANCNTNALNSAFITNMDSITHLNSLQNINDSISFSHMPELITHSSSSSTYQIPPLISKDDMVQIPESTEIKEESGSLNTASFVQAETAHAINTLEINGMADNRSKACDFGEVLGQYIWPESDELFLEDPFFNEI

>Maker00019076 EpMYB68

MGRSPCCEKQHTNKGAWTKEEDQRLINYITLHGEGSWRSLPKAAGLLRCGKSCRLRWINYLRPDLKRGNFTQEEDDVIINLHSLLGNKWSLIAARLPGRTDNEIKNYWNTHIKRKLYSLGIDPQTHRPLNAAAAAAAAAITAPVISRNKRNSSSNDNNRKIKSVSGEDSNSNSSSGITTEESTYSDHHQLSLELSIGLPSPNNNNNVVVVNPSDNMKRQRQQEEQVVLRWYGNDGSGQQVCLCCSLGFQSNSGNQACSCKAMRSTSSVITTPTAAAASDKNVFRFFTPLNI

>Maker00018990 EpMYB69

MGRHSCCVKQKLRKGLWSPEEDEKLFNYITRYGVGCWSSVPKLAGLQRCGKSCRLRWINYLRPDLKRGMFSQQEEDLIISLHEVLGNRWAQIAAQLPGRTDNEIKNFWNSCLKKKLLKQGIDPTTHKPLITEPQPIKEEEKSVTDTETLTPSMQIPFLVSASTTSLGFSSSQGSALVGSDSNQFENNNGLDPLSLFELQMTGFDQSGYGFNMCSIVPSLTSSMTEFSSDNNSGSRISSSNSSNMCRISNSLMMDPFYQFQVNDMKNEEDYQLMKTGSWKEDGHNILTQNSIDFTATYPFTSLSEDLTPANFDAFHHM

>Maker00018800 EpMYB70

MDPKANISENYVSQPMQLQEMNMKSNIQDLELSNFMVPLSQGNFLQDLFHMDPFQAHGSSSNPVFGIRTANLANPSDNFSYECSSTDFDFNESKPYVKGSANKGLAHVMDDFRPKGNNYSLNVSRERNIQMDMVDTDLRKFYLPFESQETKHMNFVVPDDEVSIISPDQRFFGRVILNNKNNRLSTSVRRACKVRNKSNVVKGQWTVEEDRLLIQLVEQYGVRKWSHIAQSLPGRIGKQCRERWHNHLRPNIKKDTWSNKEDKILIEAHIEIGNKWAEIAKKLPGRTENSIKNHWNATKRRQYSKRKCRSKYPRHSLLQEYIKSLNLDKNPPIDYRKKSSSSAKPNTARKAAAAASDQFCPDNDRLVPIHDFNEVSDFCFDDDINLFQEGCNLDALLDDMPCAPPILNVGDFEAKLQYYGDVANDDVGGNNHFDSGSLLQELEFKKEMDLVEMEQILKDDIEMGGCVSEA

>Maker00018818 EpMYB71

MGRPPCCDKIGVKKGPWTPEEDIILVSYIQEHGPGNWRSVPTNTGLMRCSKSCRLRWTNYLRPGIKRGNFTDHEEKMIIHLQALLGNRWAAIASYLPQRTDNDIKNYWNTHLKKKLVKKGENGMEGSSNDPQDGGYSLSSSQPNKGQWERRLQTDIHMAKQALCEALAIDKPSPSINPITTHNFPISDSKPSLSYNPITSQWPNQAAAALSSSSSSYASSADNIARLLQNWMKKTPNSISCSTSSEGAQSGSGATTPDHQGLDYSSLLSFNNSSSNNMNNNSDVSTESNSLLQVESKPNLDTHVPFTLLENWLFDDATASQGHEDLLNMSLQETTADLFQTAAAQLAKDKFQGLINN

>Maker00015408 EpMYB72

MRKPSPSTTSRAAQGAGANPSIKTSTPCCSKVGLKRGPWTPEEDELLSKYIEKEGEGRWRTLPKRAGLLRCGKSCRLRWMNYLRPSVKRGHIAPDEEDLILRLHRLLGNRWSLIAGRIPGRTDNEIKNYWNTHLSKKLISQGIDPRTHKPLNPNNPSTSTATSSHHNTKSSNPTPAAQPNPDNHPLTEQHPNPNFHLDPYDQATTALALVPGSGFEDLPAAAAIPFSCNTSANNNSNEQQVDDDINYCTDDVFSSFINSLINDDAFSAHPQQQHPPPPPYNVAWESTLVEPNNNMPDADRVR

>Maker00016037 EpMYB73

MYWGGMAGNNMGWGMNMIEEEGWRKGPWTAEEDRFLIEYVRLHGEGRWNSVSRLAGLKRNGKSCRLRWVNYLRPDLKKGQITPQEESIILELHARWGNSDKFIPRPSLMVVKNIQVVNNCKKFTSTEEQGCFYSMLNGNASASEASSDDTFWDGLWNLDDLHGNFSVANKANLHNLVAPYC

>Maker00015803 EpMYB74

MVRAPCCEKMGLKKGPWTPEEDQLLVSYIQKYGHGNWRALPKQAGLLRCGKSCRLRWINYLRPDIKRGNFTEEEEDTIIKLHEMLGNRWSAIAAKLPGRTDNEIKNVWHTHLKKRLQKSNQPINSETKTRASKPSIKRCISNSSTVTTQSDPASFTSFPEKDTTSSVSACTTSCDFSYPALTVGKGNNMNISINIKCEDMDSLEPILPQIDESFWSEAATDDNNLTTDNELPVHQYPFDSIESFQQGYGYSSNFDDEMDFWYDIFIRSGDSIELPF

>Maker00015412 EpMYB75

MDMESSEVATKLQNDDDLDLRRGPWTVDEDLTLINYIATHGEGRWNSLARSAGLKRTGKSCRLRWLNYLRPDVRRGNITLEEQLLILELHSRWGNRWSKIAQYLPGRTDNEIKNYWRTRVQKHAKQLKCDVNSKQFKDAMRFLWMPRLVERIQASAANASVSPTMSTITTTAPNAAYSYNNFEFGSASQGNYTTENSSTAASSESLGSTQVSPMSELAEYYYGRVPIGNTGNPNPNYSQTADLSYSDCMSSLDMQAMEPNSNNHQYWVQSAGDSSDNFWNVENMLFLQQQLGDNM

>Maker00016264 EpMYB76

MGRSPCCSKEGLNRGAWTALEDKILRDYITIHGEGRWRNLPKRAGLKRCGKSCRLRWLNYLRPDIKRGNISPDEEELIIRLHKLLGNRWSLIAGRLPGRTDNEIKNYWNTNLGKKVKDHRRQTNVTSSGRLSVRPKNDKTCKTNPITLKPKTSSPPSPDSNLKESLSNVVRTKATKCSKVFFSPRPPSSRQFSHKNSSETESFRPVDEAAAYRDQTTEVLNVNGDNGSTSSFGVGKDHHHHHHPELSSADLLVDFNVGEICLSDLLNSDFSEMCDFSNNSVDGLSPSSDQPFVFSDEILNGWTQSSGTETNVGKNLHLFTSFIESSEEWLAE

>Maker00009536 EpMYB77

MGRQPCCDKVGLKKGPWTADEDKKLINFILTNGQCCWRTVPKLAGLLRCGKSCRLRWTNYLRPDLKRGLLSDFEEKMVIDLHAQLGNRWSKIASHLPGRTDNEIKNHWNTHIKKKLKKMGIDPVTHKPLSEQTQNPPQNQQLQEQHQFQQQQLPDTPDLRIDQNGDPKSRYTSYESSSSLTESKEDEKLQTPLFDDIMNGFCTDEVPLIEPDEILMPSGPTSSSSPSSSSSSSSPRIHPTSSETWSSRNLSGLVITVMTITTATTTVWDCGMMIL

>Maker00010278 EpMYB78

MEKNAGLSKGAWTQEEDNLLRTCVLNYGEGKWNQVPLRAGLKRCRKSCRMRWLNYLKPNINRGSFTEDEVDMIRRFHKLLGNRHACFLVYIYKWSLIAGRLPGRTANDVKNFWHTNMRHMRKEENQRMKAKETISLEEEETEKVEGHVVIKPQPYTFSKNSTWLRRNKVFFCNGETSAGGGGGGFEQPFRPADHHDHSSKSESEEWWKSLLNMEGDEGQDGNSMIEFFDQDFLKMEGLDSCWSHHYV

>Maker00009477 EpMYB79

MRKPCCDKEDNNKGAWSKQEDQKLIDYIQTHGEGCWRSLPKAAGLHRCGKSCRLRWINYLKPDIKRGSFAHDEEDLIIKLHALLGNRWSLIAGRLPGRTDNEIKNYWNSHLRRKLMKMGIDPDNHKLHLHQTLRLSATHHDAATSSQSFKEKPSNLNLNLTIAFPSRSQNLDNSAESSKNVNKDMDHSQPEPTLPLFN

>Maker00010578 EpMYB80

MGLTSDNVASDRVKGSWSPQEDATLLKLVRQHGPRNWSLISIGIPGRSGKSCRLRWCNQLSPGVQHRPFTPTEDALIIQAHAVHGNKWATIARLLPGRTDNSIKNHWNSTLRRRRAVEMSSASGSSESDSILVMKKRSVPCDTPNASDLKESEQPLKRQCLRRISPENDSSKDPMAGPVTTSLSLMPPGERVDDKVKEEKEEEEEDEDEGEGSENGERKEEEEAGLMRMMQRMIAEEVRNYIESLRAHDRLVLRTELGLQLDPSSIHK

>Maker00009955 EpMYB81

MASLKKDLDRIKGPWSPEEDEALHRLVQNYGPRNWSLISKSIPGRSGKSCRLRWCNQLSPDVEHRPFTPDEDDTIIRAHARFGNKWATIARLLHGRTDNAIKNHWNSTLKRKCSAITDPLNPEPNDNQPLKRSASVGAATTVSGLYLNPSSPSGSDLSDTSLPAMSPTRVCRPFGRNSAPVPCIQQPETGSSTHDPPTSLCLSLPGSDSREASNHGSGSIQPISPTQLQPSPPFPAPAAPAGGESVHQVIVPAPFSQRCDQFGREKQFFSAEFLAVMQEMIKKEVRNYMSEIEPNGLCIQTEAIRNAVVKSIGMGRVE

>Maker00010013 EpMYB82

MGRAPCCDKANVKKGPWSPEEDAKLKSYIEQHGTGGNWIALPQKIGLKRCGKSCRLRWLNYLRPNIKHGGFTEEEDNIICSLYVSIGSRWSVIAAQLPGRTDNDIKNYWNTRLKKKLLGKPRKDQQSRSKSKQEGANRITVSNSLLLAENSLNTAIPQPYWPHIPVISPLPYSNQEPSFNDQDSIRKLLIKLGGRFSDEDDHDYHPTLDALNINPHQFPVDTACTQTVCEEQANMAFSSSSSCMINNGMIVNNNQIGLVQETGRLEEIVCSNSTYTYPQRLDGLELLFGDDMMVNEKIAPPADTTSLISLVPSNYQGMPQYTPQQCAFPELRYHRP

>Maker00010162 EpMYB83

MGRAPCCDKANVKRGPWSPEEDATLRTYVEAHGTGGNWIALPRKAGLRRCGKSCRLRWLNYLRPDIKHGGFTEEEDNIIFTLYGQLGSRWSAIASQLPGRTDNDVKNYWNTKLKKKLMAGKLNLKTLTDSGTLPSTPLLTHNVQTQNQFPDFPALPNQNSSASTLPILTDANSAFNINTHTVSFDPAQLYSPGFVDVSEINAGSRNSHMMSLSQEGSSISDSSLTAMDKCLSLRDHSGDEATQVLMNFGFGFPCDLVNGFYFQDKIGELAPSCYPELVDFSYADMKPQGLNQNVV

>Maker00010600 EpMYB84

MGRSPCWSEGGGELNRGAWTAQEDNLLSSYIMAHGEGKWTQLPKRAGLNRSGKSCRLRWLNYLRPDIKRGNITRDEEDLIIRLHRLLGNRWSLIAGRLPGRTDNEIKNYWNTTLKKLRRQSSTVSAQHPMSESTARRDDVTQPRVIQTKPGKCKNVVILQYPSGPKENSRAEADQTSTQMKFTEQSHNSHTPEPQVDVDEHHWFHENYSYSDVNLRGRDNSELQLDEAELRTNWWATGSLEVDDGISATWEKDSLALLLESDDWTLVR

>Maker00009976 EpMYB85

MNNKTVSSYLFTHKIKFQSYTSHFVHCSNKSPYLPVAETNAPRMRAYIINSTQHVSRPIGTLIHSYNIAHVIGVGFIYSQRLLLFFFIQREREMGRRPCCSKQGLNRGAWTALEDKLLISYVQAHGEGKWRNLPDRAGLKRCGKSCRLRWLNYLRPNIKRGNISHDEEDLIVRLHKLLGNRWSLIAGRLPGRTDNEIKNYWNTTLAKKLRPSEPHPPTKSTTSKSRQVTEVTRFDPQPQPEIIQTKATRCKNVVLLPPQHPNEHYCNFNPLASNQMEFDEAHLTTQLTNQSEGSHALRPPVHENQRSDVNMWGCGSEDQCAMNAENLCGDEVFNLDLPLPINELTVGGWTTTATRCFEGEENSDMDLDSLALLLDSVDWP

>Maker00010143 EpMYB86

MVRTTSPSNEGQKREAWTAEEDRMLAGYIRIHGEGKWKNIPKQTGLRRCGKSCRLRWMNYLRPDIKRGNITEDEEDLILRLHKLLGNRWSLIAGRLPGRTDNEIKNYWNTNLAKKGQQKRSKTKSQNWKRETAESGGSQEAYERVGLEPTESASPHVVPDTLTPIQPMNDNYGDSDVAAVESGAFDGQSLSPAGENVDGCLMDFDTGDVDLSEFLDFDSTQLTDFEMFNVKEW

>BGI_novel_G001198 EpMYB87

MEGNKTTPASSDGLSEGAQKFRALHGRTTGPTRRSTKGQWTPEEDEILRMAVQRFKGKNWKKIAECFKDRTDVQCLHRWQKVLNPELVKGPWSKEEDEIIIDLVNKYGPKKWSTIAQHLPGRIGKQCRERWHNHLNPSINKEAWTHEEELALVRAHQIYGNRWAELTKFLPGRTDNAIKNHWNSSVKKKLDSYLASGLLTQFQDVPRVGHPNQPVVSTSSRLQSSGEDNGHKGTEGEELSDCSQESANVSRFPSARDMTTVVLNTTEELNPNEDFSLRKEHIHSQASCIEPYYISLDDVTICISDVAHEEVSCSDFVEQTFSHDPGNSASRDTQLNLHALPSSNLDQDCSLLRSDSADCFQSHDIVNVPFHTSEGLGISTFMGPECMDPAKQEHMLISNKACCRVLHSEAINDGCFSSGEYNKNANTVSSGCISFSANHLTSI

>BGI_novel_G001196 EpMYB88

MEGDKTIQAPSDGFSDGVQKIRALHGRTSGPTRRSTKGQWTPEEDDILRKAVQRFKGKNWKKIAECFKDRTDVQCLHRWQKVLNPELIKGPWSKEEDEIIIDLVNKYGPKKWSTIAQHLPGRIGKQCRERWHNHLNPAINKEAWTQEEELALIRAHQIYGNRWAELTKFLPGRTDNAIKNHWNSSVKKKLDSYLASGLLVPHVGHPNQPMISTSSRLPRSGDDDDHKGIREEVSECSQESANASRLPIAGEMAEAVLHSREEIKPNEYSSLGKERSPSQASCSEPYYIPLDDITISNSNMVHEEASSSRYNEQKYSHEPGNFAGKDREFQLHALPSISSLDLGQEFSLLQRDSAVSDENQDIVNVQFPTSVGLGISTSMDSSKPLDMLIADDACCRVLFSDAMNDGTFSSGNHNVGVDMVLSGCTSNLCQSSDIQISDAGPTSAALVITCPTRSNNFIESSCSQSFPTVPSMPPANCRLEYTAEASQLYGGDDLHFASRTHDSFIYPNDSSISPCIDIIDSAEMHDGPDIVNDASRLVPVNNFGCASDTKRSYNLMDEKTDVHTEHEDTGSLCYEPPRFPSLDIPFFSCDLIQHGCDMQQDFSPLGIRRLMMSSMNCPTPLRLWDSPSHDDSPDALLRSAAKTFTGTPSILKKRHRDLLSPLSEKRIEKRLETDMTSTLTTHFSRLDVMFDDNEAQAAQVLPSTSIQKNKFGDSDKENIDPALKEHVEGKRESATLGYEESEKGTGGNDSQDFQPPLDIDSKLEVYLEAASASEIVQQPSGVLTEHDMNDLLLCSPNQTHLDSDKAHGSSARTPKNMNNISLEEALNQNIPPKSLYENPHISDCSPSIRVREHEISSIPVTCVPSHSSSGPGQNSGDHTGNDAGFESFNIFGGTPFRRSIESPSAWKSPWFINTFLPSARIDTEITIEDFGYLMSPGDRSYDAIGWMKQVKEQTAAAYANAEEILEIDSPNAKAGGKDQERSDPHNPSQSASNTLMERRILDFSECGTPGKDNNKSSATTFSSPSSYLLKGCR

>Maker00009707 EpMYB89

MEDSFAATASAVDQDSKTCPRGHWRPAEDEKLRQLVEQYGAQNWNSIAEKLQGRSGKSCRLRWFNQLDPRINRRPFTEEEEERLLAAHRIHGNKWALIARLFPGRTDNAVKNHWHVIMARKQREQSKLSGKRVFQDVYHDSSSNTSSSNYKRSKSQDLFSSPTFFDFRNIDRNRIIAEYSPSVSLASWNFASMAKHGKDNSYFNPCFNIYFSECSKGSDRYLHRIYPNSASSYGGILPNCSKVVSGPYGSVLGCAGDDDGRSRTTKKELMNLSDTSSTFAKRVSTEQEQADEHREEVPFIDFLGVGVSSS

>Maker00001763 EpMYB90

MKERQRWTAEEDALLCAYVKQYGPREWNLVSQRMNMPLNRDAKSCLERWKNYLKPGIKKGSLTEEEQSLVIRLQEKHGNKWKKIAAEVPGRTAKRLGKWWEVFKEKQQRERKEINKTIDPIDDTKYNHLLETFAEKLVKERPSPSYIMAASNGGAFIHTDAPAPSSAMLPSWLSNSNSTSAIRPLSPSVTLSLSPSTVAALPPFQWLQSERMPENAPPVIGNATTHRTIPAFGDNMLVSELVECCKELEEGHRAWSSHRKEAAWRLRRVELQLESEKACCIREMKEEIEVKIKALREEQMAALDRIETEYREQLAGLRRDAETKEQKLAEQWAAKQSRLGKFLEQISCRPWIAEPNGC

>Maker00001934 EpMYB91

MNNITSGVDAPKVPKSRRSMFAIEEVSAKGNVGGEVALKKGPWTATEDAILMDYVKKHGEGNWNAVQKHSGLARCGKSCRLRWANHLRPELKKGAFTEEEERRIIELHAKMGNKWARMATELPGRTDNEIKNYWNTRIKRMQRAGLPIYPPDICQRVQSQENPNVGTLMSGATQHSDIPQDGSFNVPDLDFKNYDFSRGFLRYGPSIFDAPESSLFEQTAGAPDSDIILLANPLKRFRESDMLCNRLDNSSSCMIPAFDKFVDYTAEPHHHLSSPSNLSLDTNDQFYIDNLHGSHATLNGNTSSSAPISEAMKLELPSLQYLETQPGSWDAHASPLPSLESVDTMIQSSPVEQYELCNVTPDSSGLLESIIYETRLPKGSNNDSLWQIPDTLNVAKSVTEWDVVKSETEWDRQWDIYSPLGHSSASVLTDYTPVSMCSVDDLPFFEYTKGPDMRNETVSQYPFPSVSDRNETSGQIDITRPDAVLNMCWFRDEPGFH

>Maker00011123 EpMYB92

MAEEAKNEGEGLGTTTGEGGGGAVVAEEVALKKGPWTAAEDAVLIEYVSKHGEGNWNAVQRNTGLARCGKSCRLRWANHLRPNLKKGAFSPEEEKLILELHAQFGNKWARMAALLPGRTDNEIKNYWNTRVKRRQRQGLPLYSDEPDRSTTSPKPITPRFEFLHHHPHHSLSSPTPPPHSPLSSPLQPKSTMSDPMIPLSSPSSSTVSPHSFIFNRPAPLLCNPPGFKRFKTSSGYPFRLAPTSTIPTSVSLPDLPTVATPQSAAFAGFEFPGQFNSNMSQIYQAPLLDSDRGGVLNPFPNFSPRLELPSNHFPQAHAIEPEIKFEVQMNNPAALKISGGGNGFPEDPLFEAQAMASGQTTLKKRSYSCLNEGNDIFYGYRGLDDLPLNSSYWSSNAELRLKEEAQDLSKSLNDDLSSMLSVVPSSISISDWQTGNAAEVSNVESSGVVPNDNFGLDNKPITSLFPITTTTNHNENPGCYSWDDLPGLC

>Maker00013009 EpMYB93

LRPTKFHFSIPPAHNHAHINPTLLSIDPCFLSTMGRAPCCDKNGLKKGPWTPEEDAKLIDYIHAHGAGNWRTLPKNAGLERCGKSCRLRWTNYLRPDIKRGRFSFEEEEIIIQLHSMMGNKWSAIAARLPGRTDNEIKNYWNTHIRKRLLRMGIDPVTHTPRLDLLDISSILRSVLGNNSLWNLQGLLGAQALMNPDLLKMATALLSMNNENPELLSQNLQQTIQPCNSQVQAQTGTGPATAQFNSQFQMPMETNNVEGFCGDLRNMGFSDSQEISIPSCMPDNFNLLQQNQAEYLGNPILLQNMRYDSVLSTPSSSTTPLNSPSTYVNNSSADEERDSYCSDFFKFEIPENLDISNFM

>Maker00013170 EpMYB94

MVRGPFFDENGVKKGAWSEEEDQKLISYIQAHGHPNWRQLPKLAGLTRCGKSCRLRWMNYLRPNLRHGNYTPQEEEIILKLHEQYGNKWSLIAEKLPGRTDNHIKNHWHSQLKRRSRERGRKDSELRNKKPAGCNNTNKNSESDIMKVVNELNNILESSSLYRCTESTTSTPADEDNSGSLPSVETESAMKNSPEEDYNSVASWEGYRFQEFSGDFWTEPFISEDSYSEYCYHPNMGVGGSSGDIICSYDGTPLFY

>Maker00012803 EpMYB95

MGRTASYDESGIKKGSWTPEEDFKLIAYVTRYSCWNWRQLPRFAGLARCGESCRLRWLNHLRPNIKRGNYTQDEERTIIALHQKLGNRWSAMAAQLLGRTDNNIKNHWHSTLKKR

>Maker00012629 EpMYB96

MRNPRSDKNGLRKGTWTPEEDSKLIAYVTRYGCWNWRQLPRFAGLARCGKSCRLRWLNYLRPDIKRGNYAKEEDEIIIKLHQQLGNRWSAIAAKLPGRTDNEIKNRWHTILKSKHQVQLENDSVITHDKNETVAPSNHHSESIPYSATSKDPDNGKSWPVSTSSESSDITTESNEDLVGDHDFLFWMRMLSQ

>Maker00012428 EpMYB97

MVRTPCCSQNGMRKGTWTPEEDMKLIAYVRRYGCWNWRLLPQFAGLARCGKSCRLRWMNYLRPDIKRGNFTPQEDDAIIRLHQKLGNRWSKIAACLPGRTDNEIKNHWHTSLKKRSEQESEPHGKDKALKPKDQIPFGSRQLEIRDSATISTITAPSSQCDSIVGASQISGTSGTFPVPSPDSAFSSEENLAVKDYHNLAFPDAFVESMSENFWTEPYMVDISYVPDEQLVPLSPEYEHNAHLWGYNDLYEEYIV

>Maker00013083 EpMYB98

MSSFKYWLINSTVTCGVEMEFNTNLSEARLSPSIGLYIHHRTCLFLHIHTVEMRTPRSYNNGLKKGTWTPEEDRKLIAYVTRYGCWNWRQLPRFAGLARCGKSCRLRWLNYLRPDIKRGNYTKEEDEIIIRLHQQLGNRWSAIAAKLPGRTDNEIKNHWHTNLKNRHQIQLQNNSVTRDKDEIILKSSNHHSPGVTDHAATSKDPDNGKLWTQSISSESSDITTESNEDLVGDHEHSFLDAYAEPLSGSFWTDPYVIDSDISYFPYTESLPPENESQFSAPISNIGQLWTHSGFYDQYSGF

>Maker00012776 EpMYB99

MLALPSLSLSFIFLSFPLFSPKPSNSIPFSLPPSYSPFIPLHLSLSLSPPHHHYASTSLSLSLSLLVSPALFSISPLFSLFSTWERREIFLLNLHRSRHHILQPQDGRQIFTCFSEMPRMIRSDEVEEEEMVVLEAAKADASGEAKQSMKKGPWTATEDAILIEYVKKYGEGNWNSVQRNCGLTRCGKSCRLRWANHLRPNLKKGAFSQEEEQIIIELHAKLGNKWARMASLLPGRTDNEIKNFWNTRMKRRQRAGLPLYPPEVQDEAAMLHLRQNRQYQPPPPHHSSSFSFSSLLSSSNNSHKLIDQLNNSDSDSAYDRSNQHHQNMALPLLSSVSPYGFNYSGVLKTEISSSLSETPPSSYSTSGAHCLMGGSPIGPSVDYYEVAPLSSSTEGNSGLLDALLVESQALSHSENHKSKSKDSSAGRKLALKRKNREDEEDKDTVQLESDGKNGKFQRDDLSPCQLSTEKKAVREKDPLEEINSMDDDLFSLLNNFPTEMPVPEWYRRGERQSLGFEDQQDASVGHPDQQLAWTLGTCWNNLPSIC

>Maker00012843 EpMYB100

MIQQETRKGPWTEQEDIKLVSFVGLFGDRRWDFIAKVSGLNRTGKSCRLRWVNYLHPGLKRGKMTPQEERLVLELQSKWGNRWSRIARKLPGRTDNEIKNYWRTHMRKKAQEKKRALAGTSPPSSKKTGEESFYDTGGPKTMCSVEIEGGEDQAYEQEGYSISMDDIWKDIDVSEENILEPVFDVHSCAPMRSPSWDYSSDSLWVMDQEESRIFHPMGDPIFQCYEPYLTG

>Maker00013236 EpMYB101

MRKPESGKNNNKLRKGLWSPEEDDKLMNYMLNNGQGCWSDVARNAGLQRCGKSCRLRWINYLRPDLKRGAFSPQEEELIVHLHSLLGNRWSQIAARLPGRTDNEIKNFWNSTIKKRLKLMSSTTTTSQNASESSLLSEPNNKDLNNVMGGFMPMFNNNNIIERLPMLEHGLNMQSSGGFFNSNGGPFFTPNGVDTNNSGFYIENGVFGIGSVNIGAEGELFVPPLESVNTITDNLRLENRDTNVNSYFDNINSIVNNCNISGSNNRAENRGGVENLFQEELTLGEWDLEDLMKDVSSFPFLDFSS

>Maker00019815 EpMYB102

MGKGRAPCCDKSKVKRGPWSPEEDLKLISFIHKHGHENWRALPKLAGLVRCGKSCRLRWINYLRPDVKRGNFSKEEEDTIITLHRSFGNKWSKIASSLPGRTDNEIKNVWNTHLKKRLKARSSQSNIAEESSTTSVSSDSFSSEKIQQHSEKQVSQEPIKDLKESPPSSSSSDHSKPEEDEQMIESLFNDDDPLLQIPMESLDFDIWNILENSESFQPNDFQVSSAEDEDKNKWFRELEDELGLGTSEGLNLARNIEDADMAFLESSWSYWPEISNL

>Maker00019873 EpMYB103

MGKEKMNSSSNMMKRKLRKGLWSPEEDEKLMRYMLTNGQGCWSDIARNAGLVRCGKSCRLRWINYLRPDLKRGAFTPHEQHLILHLHSLLGNRWSQIAARLPGRTDNEIKNFWNSTLKKRFKNNNTSSTSSPNNSSSSLSSSEPKNHVLSGNSSTIDPLISMNYSNSSSSSSSSSMQVPIIFGEQLTSDPFFALNHHFDTNIEAAAGNFSLPAEVGNVEEGLYNGDFGILLDPNNIEDTSVPNFEDISVKSHHNNFNNSCFNYTNDDHQIIQRSNNNKADEQEDMYGFGNSNMVINGENLLGDQWDLEGLMQDMSSFPYLEFSKLVE

>Maker00016649 EpMYB104

MSRTSKRFEDEILLKDQTGSHLNDESDGGSAGGVVLKKGPWTSAEDAILMDYVKTHGEGNWNAVQKYSGLSRCGKSCRLRWANHLRPNLKKGAFTAEEEQLIAELHAKIGNKWARMAAHLPGRTDNEIKNYWNTRIKRRQRAGLPLYPPEVCLQSRQERQHGQSNVGINGIERGNHGLLAKNNDEIKNAMLNSLTANQGFLPCVPELPDISSSSILQKGHGPSPNCNFMPSTLPHHKRLQESAIPFLGSTSINGNGFYPFDHIWNNTSDMIAQSFGMHSPLDHSPSFESSLFYSHSLSNGYTSTSKPASESVKLELPSFQYPETDLGSWGTSSLPPVLKSVEVVIESPSPTSALEPNCSSSHNSGLLDDLIHQAKTLSSLRNHYFDRSSSSSSTATPGERADSSTLNVYRTEWEDCIDPVSPFGATSILNDCPAVCADKSIVNEISPAQASIGDMKLESVDQVWIPNSEDQTMSMLDIAGPDGFLASYWFDHGADAVTKLSTDWASTTSSEVCGNGSGAWSDMPDVFQMSDLPSEN

>Maker00023829 EpMYB105

MGRSPCCEKAHTNKGAWTKEEDDRLISYIRAHGEGCWRSLPKAAGLLRCGKSCRLRWINYLRPDLKRGNFTEEEDGLIIKLHSLLGNKWSLIAGRLPGRTDNEIKNYWNTHIRRKLLSRGIDPATHRPLNEASQNPSFSSSASAAKQQESEIVIGGEDSKRPVVLEVCPDLNLELTISLPHHQQNQPDQPSLKNDGIVKVGGSNNGSSTAGDGYDFLGLKSSVLDYRSSVNPNKLERTHKLMYECSF

>Maker00023465 EpMYB106

MKAQPRDVDRVKGPWSPEEDESLRKLVQRYGARHWSSISKSIPGRSGKSCRLRWCNQLSPDVEHRSFTPDEDEIIIKAHSKFGNKWATIARLLNGRTDNAIKNHWNSTLKRKYSSLVADEGAAVDGPPEKKSATVDRAVPIRSISSGGHCYSPSSPSGSDVSDSSLPVVSSSTAIRPVGRDWGNLQPTHQVESHLSPTFNSSKEPCTDLTLSPLGAETKESQKIRNFGPCAEAVVQSEKLSSMATFGPDLLIVMKEMIREEVRNYMAGYNDRLCSNRDGVSAGVSKINIMLVQGGYLVELELKLGAVNFYLNSEDKTAK

>Maker00005527 EpMYB107

MYYSTGGAFRSEESRKIKLIGDMGHHSCCNKQKVKRGLWSPEEDEKLINYITNYGHGCWSSVPKLAGLQRCGKSCRLRWINYLRPDLKRGSFSPQEAALIIELHSILGNRWAQISKHLPGRTDNEVKNFWNSSIKKKLLSHDHLLLPSLATSNGTSVESFFTLTPNPNNNLILNSHHPHHHQDQLYLPTSSSLIQGFEFGHGAEDLKIGNLLHIQNPLQQITPSNCIPSSYEDTWSLVDQESHQILKSDLTNLFSPHYIADNFIMNPSSILQHHESQPVDPNIEPKIFESTIEDYNVCGMLYSSSCASQEQDPLIARIIQSYPSSGFVHPQDQSQIADAAANHMEFIDAIMSSLPLSSSSSSSSSSSLSANNQIVTNPLNLPSCWEP

>Maker00004936 EpMYB108

MGRAPCCAKVGLHRGPWTPKEDALLTKYIQAHGEGQWRSLPKRAGLLRCGKSCRLRWMNYLRPDIKRGNITPEEDDLIIRMHSLLGNRWSLIAGRLPGRTDNEIKNHWNTHLSKKLRNQGTDPITHGKLSSLPSPDNHSSRKKVSKRKKKGKAPENNGGEEEETEKKTIVYLPKPIRIRACLSSSIPRSDSNHNLNSMDSNSASGSTSQEKEEEKKGEVVRGENINNNDYSNYNYKYKYNGDEEDGFVGLLEEEHELVNGSDMEGQSEFPTSAEQHGTLEKLYEEYFQLLNNQESQQELESFAESLLI

>Maker00005382 EpMYB109

MGRTPCCDKRGLKKGPWTTEEDEILVDYIKKNGGHGSWRSLPKLAGLLRCGKSCRLRWTNYLRPDIKRGPFSLEEEKLVIQLHGILGNRWAAIASQLPGRTDNEIKNLWNTHLKKRLICMGLDPQTHKPLSSSSPSNPKATRHMAQWESARLEAEARLSKETSLLTKPSPHSDFFLRLWNSDVGDAFRSLNPNALLSSPGSSSSTKCTASFSAVTGPLPAGVGRVVRHRVAAAVGFSHIP

>Maker00002381 EpMYB110

MAEVKSEECCLENKQSLAASSSSLSESSGSALPKSPGICSPISTSPSHRRTTGPIRRAKGGWTAEEDETLRNAVAIFKGKNWKKIAEFFPDRSEVQCLHRWQKVLNPELVKGPWTQEEDDKIVELVLDYGPTKWSLIAKSLPGRIGKQCRERWHNHLNPDIKKDAWTLAEELALMNAHCMHGNKWAEIAKVLPGRTDNAIKNHWNSSLKKKLDFYLTTGKLPPISKNSSQVDVKDKIICSATKKLLGSSDKGLDAATETSSETTDINKQDDSCKNHLRLTTRDVGDSSNVLANEADSDCTECKPKSSILDFSCSDSEPLSRDNFGINSERRNEKSGLNGEMSSSRLITQPFSQESSTCGSLCYEPLQMEISVPSDSLYLNTFCPQNDYSSTPMTPPVGDFTPPCVKGSEIFRQNAESILKQSPESILKMAAKTYKNTPAILRKRRTGIQTQVTPSKVMKVDEVSHASNGQQRNRNNSDPEVGRSSESPASHGHENDIPNNKVFNASPPYRLRSKRTKQTSVVKSVEKQLEFSFDQEKCNINLRKTEKSSK

>Maker00014644 EpMYB111

MGRAPCCAKVGLHRGPWTPKEDALLTKYIQAHGEGQWRSLPKKAGLLRCGKSCRLRWMNYLRPDIKRGNITPEEDDLIIRLHSLLGNRWSLIAGRLPGRTDNEIKNYWNTHLSKRLSKSQSPPHHQPASKPNKKKKKKKAAKSNRNAEELEAEEEQEEKKTIIYLPKPIRVSALPASRDEDSKNVSASASGSSSQELLQKDMDMSYEGWGEENDVFGFFMEEDHNNDLVNVSISEEGNNMNNSNDIEYESLEKLYEEYLQLLKDNNDSEEIPGEMDSFAQSLFV

>Maker00014977 EpMYB112

MGHHNCCNKQKVRRGLWSPEEDEKLINYITSHGHGCWTSVPRLAGLQRCAKSCRLRWLNYLRPDLKRGSFSSQEATLIMQLHSILGNRWSKIAKHLPGRTDNEVKNFWNSSIKKKLLSYNMFPTSLSTFSDLLLPDNANVGTFFPSSNPNLLLNYTHLHFLQPDHHQLYHPTLSPTPRGLADIKVDNTYYSPNNNFLNVQNPIPESTPSIITMPSSYEDNGDDHNTHDTWSLLGMNTPNQENRITKSDQPLLAEKFMNPSSMIMQHYDDETYRDERLVEPIGPKISESFEDYVLSSIPSSYSQELLQDPLARILSYPSGFCPQDHQTLSVNQMEYVDDILSSSLPLVNNKPYRSF

>Maker00015287 EpMYB113

MGHHSCCNKQKVKRGLWSPEEDEKLINYITTYGHGCWSSVPKLAGLQRCGKSCRLRWINYLRPDLKRGSFSSQEAALIIELHSILGNRWAQIAKHLPGRTDNEVKNFWNSSIKKKLLSHDMLPPSLSTFSDHLLHHNASVETFFPSSNPNLLLSYSHLHLQPNHHQLYLPTSSSVPQALNDHHHNNDDIKVGITSYNPNNSFLHVQNPIPESTQPNIFIPSSYDDDNSNTNNIWSTLGQSTPTQENQITKSDHPLVAEKFMNPSSMLMMQHYDHDEHLVESIVPKICESFEDYVFRRIPSSSYSQELLQDPLARIQCYPSGFFPQDHQTLFSATQMEYIDAILSSLPSSTTNPIVPSSWEP

>BGI_novel_G003098 EpMYB114

MAELRTEERCQENKQSTAASTSSASEGSASAIHKSPGRCSPASTSPSHRRTTGPIRRAKGGWTAQEDETLRNAVAAFKGKSWKKIAEYFPDRSEVQCLHRWQKVLNPELVKGPWTPEEDDKIVELVSKYGPAKWSLIAKSLPGRIGKQCRERWHNHLNPEIKKDAWTLEEELALMNAHSVHGNKWAEIARVLPGRTDNAIKNHWNSSLKKKIRILFSYWKPSTNPKEQFTRFCERYNKTLCK

>BGI_novel_G003094 EpMYB115

MGRKPCCDKEWVNKGTWSAKEDQILKNFIEVHGDGRWSEVPKRAGLNRCGKSCRLRWLNYLRPDIKRGNISEDEEDLIIRLHKLLGNRWSLIAARLPGRTDNEIKNFWNTNLSKRVEQKKCMHNKPSDPITTRTPIIISTSQTSAKHTKISSLSTDIYPAVSSQSQHVIRPQTARCTAKVMVPESNGYILTYA

>Maker00023165 EpMYB116

MKERQRWRAEEDALLRAYVKQYGPREWNLVSQRMNTPLNRDAKSCLERWKNYLKPGIKKGSLTEEEQRLVIRLQAKHGNKWKKIAA

>Maker00023279 EpMYB117

MYTCRFFSVDAKDGQMRETRSGSEFDHSRKTQTQANSNTYALFCSSLSLFCLAQLLYSAVRGVKMEADEKTVVELDGPTAADGGSGDEGLTALGDGGGSNSQGARDRVKGPWSPEEDAILSRLVSKFGARNWSLIARGIAGRSGKSCRLRWCNQLDPAVKRKPFTDEEDRIIVGAHLIHGNKWAAIARLLPGRTDNAIKNHWNSTLRRRGVELDKIKLEPCNMAEDVSLEKVKASSEETQSCGDVNSLRSSEGRDVSSVENMDDKYEDKAQTEDRLGHEIKDPPTLFRPLARVSAFSVYQSLDGSQPTTSTSRPVPMQGPLFQASKPGIEICKMLEGTYGCDLAVPNHCGHGCSNLNGENSKSSLLGPEFIEYAEPPSFSSFELAAIATDISNLAWLKSGLENSSVKMMGDSAGKVISHGSQVNIGH

>Maker00013934 EpMYB118

MEALQGDMDRVKGPWSPAEDELLKKLVKQHGARNWSTISKEITGRSGKSCRLRWCNQLSPEVERGAFSPEEDEIIVQAHAKFGNKWATIAKLLNGRTDNSVKNHWNSTLKRKYSSLIGDAEDQSFQTKRQATVDRSLTIPSVLAGHCHSPSSQTGSDVSDSSHQVVSNSTSLIRPVARPDATLKNEPCTELTLSLPGDKTDELWRAKSSGAIAEHEMALSSILLSSEKKHGGINTFGPELLSLMKEMIRDEVRNYMAAL

>Maker00013914 EpMYB119

MGRSPCCEKAHTNKGAWTKEEDDRLIAYIRAHGEGCWRSLPKAAGLLRCGKSCRLRWINYLRPDLKRGNFTDEEDELIIKLHSLLGNKWSLIAGRLPGRTDNEIKNYWNTHIRRKLLNRGIDPSTHRPLNDPANSAQNNQEAATISFASHAQQEDSNDNNAAVFVEVHRDAKRGVVVERCPDLNLELTISPPHQNQEAEDQVQPILKSGGGGRSLCFACSLGLQNSKDCSCGIVGGINGGAGGSGSAGGYDFLGLKNAVLDYRSLEMK

>Maker00003060 EpMYB120

KAKWGVSQLIRQNQNTEKGYGPLKKIISSETTSSAMAMAAGALFPLRQIKNETGLQRNGKSCRLRWINYLRPGLKRGAQNTYKKPPTDLVQIAQHLPGRTDNEIKNHWHSYLKKKVAKAKEMESHKNKQMQYASSSSDTIDSSPSNQKPTIPIADHFSSLQNNNFIKEAGQSSLPKLLFAEWLSLDQANSGIPAISCDDPFAFGNGSFDQYSDFHEAAIHGVSEGGSYGGEFDNGLTTPSAAASTDVFNSQFNIANQMVGNGVIHFPSFGMSNDLMYI

>Maker00003167 EpMYB121

MRTPVIALPSMYALNTVLGAWTPEEDEVLVQYIKEHGCGSWRTLPKRAGLLRCGKSCRLRWINYLRPDIKRGPFTGEEESTIIQIHGMLGNRWAAIASHLPGRTDNEIKNYWNTHLKKRLHSGRTLEARKSSFGPHQSNIQSESPSVRHVVQWEKARVEAETRLSKELESSCFNPCSQTKTHTDCFLQLWDSEVGQSFRMITEKETASQSPASPASSVFTNMAQERVRRCNPKLEEGTAASESGSYEFLDASDSALKLLLDIPDDNIIFSEGLEQTHSFLSPLHGRCF

>Maker00002686 EpMYB122

MVRAPCCEKVGLKKGRWTAEEDKILTQYIQQHGEGSWRNLPKNAGLLRCGKSCRLRWINYLRADVKRGNITPEEEEIIVKLHAALGNRWSVIAGHLPGRTDNEIKNYWNSHLRRKIYCFIKTLNESLPSTHLTALNTMATSSKRRSGRVRPTESPIKPDKPTPSAHSSHSQNNTVKVFEKVSKPNSTSESYTDAGSKSVGTMVPLCPSDYNGDGELGGKVETMGPYQWLDDEIMKLSYMLESEVYVNPSENYMDHENELVLSRNNDMVGGDLYVLVGKEIIGASSFDSPEISVYDYQWLDWDFNQRNLSEEKSLNMAYLWGNENGGEYRGKFMFILNTIFPF

>Maker00020342 EpMYB123

MQDMKKQHQHQYQQGKEESKKKERHIVSWTQEEDDMLREQISIHGTDKYWFSPCWTIIASKFKDKTTRQCRRRWYTYLNSDFKKGGWSPEEDTLLCEAQKIFGNRWTEIAKVVSGRTDNAVKNRFSTLCKKRAKYEALAKENSTSFINSNHKRIIFQHFYSTDAASEATATIKKMRKAHIPDAATKINYGDRSHKELESPVSQQPRVPFAVLANFNGNCVQDHHPVCNVKFSSYAQNNTQGTFLRKDDSKISVLMQQAELLSSLAENVNTENMDQSLENAWKVLQDFLNRTNKSDLSRYKYPNLQLIDLKNMAEHLKSGNEGSQPCLRQPELYEDSPGSSDYSTGLTIQPFSAGDNLEHSLYKNVGTERKSTEIEKEKGVNDCEKQVLSASTADQEFSSPIQVTPLFRSLAAGIPSPQFSESERNFLMKTLGIESPSPKRSTKALQPPPCKRALLHSL

>Maker00020255 EpMYB124

MSKSPCREKEGLKKGPWTREEDQKLVAYIEKHGHGSWGALPSKAGLERCGKSCRLRWINYLRPDIKRGKFSLQEERTIIQLHAFLGNRWSAIAAHLPKRTDNEIKNYWNSHLKKKLTRRGIDPTTHKPKLHTLGSNLSHMAQWESARLEAEARLVRESNSQFSSSSFPCSPQPVRLVLKKISAQLSPPPCLEVLNAWRNSPSLPTAEDKNKRMHDMYALMLAAADDDLKSPTFTLSFPETMSPSSATTTVGLSGENLLIPPINMINSTVAPVAVESWECMMEDNRDTEVRERRMTSLQDDDIMVAVEPFMAVRCTNAPEGLSSR

>Maker00020412 EpMYB125

MGRSPCCEKVGLKKGPWTPEEDQKLVAYIEQHGHGSWRALPSKAGLERCGKSCRLRWTNYLRPDIKRGKFSLQEERTIIQLHALLGNRWSTIAAHLPKRTDNEIKNYWNTHLKKRLTKMGIDPTTHKPKVDALVSASSSHANLSHMAQWESARLEAEARLVRESKFQLGSSSLPSVQPARLVLNKITARPSIPPCLDVLKAWRNSWSMPTTQEKSTRMHNMYALMLADVDDNLESPTSTLNFPESLLPPPVSTSVKLPGENSLVPSVTMVNSAVLTEVGSWDCLTEENPVKEIIGERRITSLQDDDIMVAVEAFMAERYKNIPEVLSSNSAMNGLNSEDNDVMIYDSNENLAGGDNLGFMDINGDGSLICSNVDFDEKNHYWNSIYSTQLGD

>Maker00020901 EpMYB126

MSAATKPELGSTEEESELRRGPWTLEEDSLLIHCIALHGEGRWNMLAKSAGLKRTGKSCRLRWLNYLKPDIKRGNLTPQEQLLILELHSKWGNRWSKIAQHLPGRTDNEIKNYWRTRVQKQARQLNIESGSERFIDAVRCFWMPRLLQKMEQNASSSSVTNMNSENSAITIACLSPPPGEFTDNAANHASTTAISTNNNNHNNPNFPDHYSLQFQEPFQTSENLTSTTLPCVFDNNNNNVCFNPVQDSYGMEGLNLESLSRTESYDIAGFDYSQSSESQWGLSNMGEYSLWNLDEM

>Maker00020487 EpMYB127

MRKPCCDKQDMNKGAWSKQEDQKLIDYIKKHGEGCWRTLPQAAGLLRCGKSCRLRWINYLRPDLKRGNFAEDEEDLIIKLHALLGNRWSLIAGRLPGRTDNEVKNYWNSHLRRKLVNMGIDPNNHRLGHKFPARQNQTMSGSSASSGLKLNDSKNNVEPTSKPQTGGDEQVSDAASGLEDESSGLPDLNLDLTMNIPSTLPTCASYEDDQKPVHQHHESKSPRNIQLASSPTLLLFH

>Maker00011362 EpMYB128

MNTSVTRKDMDRIKGPWSPEEDDALQKLVEKHGPRNWSLISKSIPGRSGKSCRLRWCNQLSPQVEHRAFTPEEDDTIIQAHAHYGNKWATIARLLSGRTDNAIKNHWNSTLKRKCASMMIDDAGAAGGFDGSFSPQPLKRSVSAGAAVPVSTGLYMNPPTPGSPSGSDVSDSSIPVTSSSQVYRPFARTGGVLPPVETTSSSNDPPTSLSLSLPGVDSSEVSNRPAEPAHIAPPTNNIPLLSVMTPAPPPAPTPTPPLVPTSAPMLVPQQVHLGRDDRSGEGAPVNQNRFSAFNFSAELLAVMQDMIKKEVRSYMAVEIFPGGSAKFTLFTVSDSWSPSLVRKEKDTRNLRRDVGWNQILKNLNI

>Maker00011685 EpMYB129

MGRQPCCDKLGVKKGPWTAEEDKKLINFILTNGQCCWRAVPKLAGLRRCGKSCRLRWTNYLRPDLKRGLLTEAEEQLVIDLHARLGNRWSKIAARLPGRTDNEIKNHWNTHIKKKLLKMGIDPVTHEPLNKQAASQETSSPLQNIPQSSNNHQIKETDGVPNSEENSSSSPAENSSGDESLLLDSICSDDSLMNSLWLDQTPLVDALWDSEPKVENNNNVMGIPSWEDNYTWFLDCQDFGIHDFGFNCFNEIESNALQTLGIEEKDH

>Maker00012152 EpMYB130

MGHHSCCNQQKVKRGLWSPEEDEKLIRYITTHGYGCWSEVPEKAGLQRCGKSCRLRWINYLRPDIRRGRFTPEEEKLIISLHGVVGNRWAHIASHLPGRTDNEIKNYWNSWIKKKIRKPSVSSAASAQSSVDHHHHHHHHLQYNFNNPNQLDFSNQDNLTTKPPPPVQDTIFSSTCPIFMFDTSSLDGSATSVLDNSNTRPEMLIQQDPMGLSSETWNLSHHQIQAFTPPSSAFTAASGMVTSSYLPPLIENVDNMVPIHEVQSCSTMDDEGEMALECLQRQELNEWVDTQQQCPNFIFWDNVEGQLGGEEIAPNSSSLGTTTLSSFPSSL

>Maker00011922 EpMYB131

MGRAPCCEERSGGVKKGPWTPEEDQKLVEYISKNGHGSWRALPKHAGLNRWSKIASHLSGRTDNEIKNFWNTHIRKKLLQMGIDPETHKPRTDFNHLINLSHFLRISSNNNINPLASQLDLTTQLTKIQLLQNLLQIVNTNPYYLLGNNHTLNPLQLQSFMNGTAQEHSNPGGGLFPQPHNQSHEGTSKSWTGVQGGCDLEASGFNDGISIDSSIGHLNQSEMAIPALLSASPATGAGVNRMDGSCNASQVSVDQSSNSSTMFESWEKLLDDKITGSDWKEILEYVMF

>Maker00004598 EpMYB132

MGHHSCCNQQKVKRGLWSPEEDEKLIRYISTHGYGCWSEVPEKAGLQRCGKSCRLRWINYLRPDIRRGRFSPEEEKIIISLHGVVGNRWAHIASHLPGRTDNEIKNYWNSWIKKKIRKPSSSTTSAQSVDHHHHQINHSNTGNNQLHFTTQDHHHNLITTKPAFQETLFSPTCPLFMFDTTPILHHGTTTSNNDSNNIIIINNNNNSRTVAEEVFQDGNVSLMSSETWNLSQHHLPPHPPSATISFTGMDTSATATISTTTTTTSTNYSLPPLIDHNMVNPIQVESCCIEEEGDMGGMDCLRRQDQLSNIDWVEETTGQQQCPNFLFWDNLGGDEIMAPSSSSNVLSSFPSSRDNSDWEIGKTSVSPL

>Maker00004242 EpMYB133

MVEVKRELEEFSFDSPNGDGHQLCFSTPVSGRRGPARRSTRGCWNEEEDNLLIEAVKKQNGRNWKKIAAYLPGRTDAQCFHRWQKVLNPELVKGSWTKEEDDCIIELVRKYGCKRWSLIAKYLPGRIGKQCRERWHNRLDPAVKKDAWTEEEESILICYHQIYGSKWAEIARFLPGRTDNAIKNHWNCSMKKKLEASSPCKCEVNTDASGVYTPQIDMLVEEEGQNLSQMVSPKWSTRLKHNIGATELVHHSASAENFCLEKDSCEGRTLGNSQCGNKIVNKPTAYFDAPLDRLAAEQDRVPLEVSSTATNEMFISPKRSKSSSDASFAVVGKSCSRLLSQPVLANYKEIVQVETKSKINKGQSQKHYPAGSKSCPTEPIEFPSKENLINGYYHTPPKSTYRNFISGSSPESVLRSLAMTYENIPSIIRKRIHRKTSIPENYETSRTPLQTIFCSPEAECLSVDCLHNLKSKQAGFMPFVHKPKTSARSLQGCM

>Maker00004406 EpMYB134

MGRQPCCDKLEVKKGPWTAEEDKKLINFILTKGHCCWRALPKLAGLRRCGKSCRLRWTNYLRPDLKRGLLTEAEENLVIDLHSRLGNRWSMIAAKLPGRTDNEIKNHWNTHIKKSFLRWVLILLLMSLSIKKLLPRIIHPLQSLVLNLTVTIVRLRFSLQRRIQFRSHHQLKILARTNLFRKIAILATTLS

>Maker00004321 EpMYB135

MAQKGKTESSQTRKFVNRGAWTPEEDSKLAQCIELNGARRWKTIALKSGLNRCGKSCRLRWLNYLRPNIKRGNISLEEEDLILRLHKLLGNRWSLIAGRLPGRTDNEIKNYWNSHLCKKVNHQEKKPNTSTPQESTPQNTWESALVEDVITGNEIVNAEVSFDVNEFFDFSPKEGSHELDWMNKYLKLDEFPESNGEF

>Maker00004767 EpMYB136

MGRAPCCDKANVKKGPWSPEEDAKLKDYIEQHGTGGNWIALPHKIGLKRCGKSCRLRWLNYLRPNIKHGGFSEEEDNIICSLYVSIGSRWSIIAAQLPGRTDNDIKNYWNTRLKKKLLGKQRKQQEAQARRANTLKQEFKRHTNNLVIMNQSSPSLLTHQPSASASIQQPYDLNNLRNLLTKLGGRFSPDHLLPNPYECEPCFPQDYHQQYPVPNSMINNCSTSNIFEGYQIENFPTDLSEMGCLTTTQQMDGFKSYNNGVMESSTATTSTESTSWADMSSLLYSPNMVSEYQEKMLPQEVAFEEPTYFGYNNNFSVHGMV

>Maker00004612 EpMYB137

MSMAVDSRKDMDRIKGPWSPEEDDLLQKLVEKHGPRNWSLISRSIPGRSGKSCRLRWCNQLSPQVEHRPFSPEEDDTIIRAHARFGNKWATIARLLSGRTDNAIKNHWNSTLKRKCASMMMIDETTTGAGGFDGSFSPQPLKRSVSAGAAMPVSTGFYVNQQTPSSPSGSDVSDSSVPVACSSHVYRPVARTGGVLPPVETTSSSNDPPTSLSLSPPGVDSSEVSNRASESAHAAPLTNTMRLLSAMVPAPSSSIAPSVAPSGAAMLVQQQVNLGLGDPRSADCGSSAANPNGSAPFNFSSELLAVMQEMIRTEVRSYMAGLEKRQLMELGTHQ

>Maker00023000 EpMYB138

MVRLPCCEESGVKKGPWTPEEDEKLTDYISKHGSGSWRTVPKHAGLNRCGKSCRLRWTNYLRPDIKRGKFSEEEERLIINLHSVFGNKWSKIATHLPGRTDNEIKNFWNTNIRKKLLQMGIDPETHKPRTDLKHLLNLSHLLGMSNNNLMSPWSHAALGLQQPADMTHQLSQIQLLQNLLQLINGNSSLQSSLPLLGNHSSINNPMEAYVNGTNSNLLNNMEPFLRDQELHASPALFPSAPSESHQDNSESWTDQEGASDQDVLGLKNSISRTSSQEMIIEAEFPAPASVLATFNHKESPKTDEVSNRSPPSSIFDAWEKLLEDDTSDSYWKELLE

>Maker00022583 EpMYB139

MGRPPSCEESYVKRGPWTPEEDEKLIDYISKHGSGSWGTVPKHAGLNRCGKSCRLRWTNYLRPDIKRGKFTEEEERFIINLHSVLGNKWSKIATHLPGRTDNEIKNFWNTNIRKKLLQMGIDPETHKPRTDLKHLLNLSHLLGLSNTNLMYPWSHAALGLQQPADMTHQLTQIQLLQNLMQLINGNSSLQSSLSLLRNHILINNPMETYVNGTNRNLLNDMEPFLRDQELYASPALFPSAPCKSNQDISESWTGLEGASDQDVLGLHNNISRTSSQEISQAAIPLPASVLATFNHVESSPNTDEISTQKPPSTIFDAWEKLLDDDTTDSLWKELLE

>Maker00023111 EpMYB140

MMKGEGVNNRNEYKKGLWTEEEDRILIEYIRIHGRRHWNRISRVTGILMRSGKSCRLRWLNYLSPNVKHGSFSEEEDDLIIRLHNLLGNRWSLIAGRVPGRTDNQVKNHWNTNLCKKLGITKQARKPAKDKVPSKSRASQVKNTQNCHMTLSSVPISPLTAVNTEHKATEIQGQESCVDNSVEAIDGAQYCWPPSDKDHFDRTFMLDDYHTFVLHSPPRLMEFFNEFDVM

>Maker00022990 EpMYB141

MKKLKQQQQQQQLGKQEESQKKERHIVTWTQEEDDILRKQISIHGTENWAIIASKFKDKTTRQCRRRWYTYLNSDFKKGGWSPEEDLLLCEAQKIFGNRWTEIAKVVSGRTDNAVKNRFSTLCKKRAKFEALAKENNASSTNSNNKRTIIQQGGDNTESTSKAAAPTKKMRRAHIPDAVEKINFGDRSHKQYAATINQQSRAPFSILAHNLPDQHEASNAKISRNAHNCKPQGTFLKEDDPKISVLMQQAELFSSLALKVNTDDVDQSLEDAWKVLQEFLNQTKESDILKYKISDLQLEDLKDIMEEFKSSNEGNQQCWRQPELYEDSPSSSEYSTGSTLLPHSVSDNLEQSLLQDIGSDVKSVQLGDQKGVSECGKEVLSATTTGQDIFTSCEEPISNDGIVFTSSSKEFTSPLQVTPMFRSLVAGIPSPQFSESERNFLMKTLGMESPSMNTSANPSQPPLCKRALLHSL

>Maker00022743 EpMYB142

MKAAWSNCPVYSSQEHSNALTASDLSSSSMGMVYVDMGSLSLSSSSSEMGNNVGGTSWGTFPFMRDHLPNTITTTDNDKDALECKASESSTDYENLADHNRENPNENSGVSAGKEADSGQSKLCARGHWRPAEDAKLKELVALYGPQNWNLIAEKLEGRSGKSCRLRWFNQLDPRINRRAFSEEEEDRLMQAHRIYGNKWAMIARLFPGRTDNAVKNHWHVIMARKYREQSSAYRRRRLSQSVYTRSTEDQNSSLATPTTRGSSVTEPPPPPPYCLVTNNNILATIPYPHFRGANGGVDYGFNGSPRMAAGIEAFSSNTTKQAPLPHSSFYAHHTPFDVFSGGRGNDMVGGALGQSVYRENRLSEYELYPQHHHPPPPPLNGFYPQEDDFNPYMVPFMQQPLQSNSNVNPSFLHNFSPNLTASNVSSGEPSSSENRDKLLSRPSPPPPFIDFLGVGAT

>Maker00002509 EpMYB143

MSSVTKRDMISSNEEENEMRRGPWTLEEDSLLIHYIARHGEGRWNMLAKSAGLKRNGKSCRLRWLNYLKPDIKRGNLTPQEQLLILELHSRWGNRWSKIAQHLPGRTDNEIKNYWRTRVQKQARQLNIESGSKNFIDAIRCFWMPKLVQKVEQKQNSPPTKSYSSLTYTNLQVSSEPSMVSTNNNSVIPSLSSSSSFQPANLSSSNYSIPSNFPSDSWQFSQLLEPSELPASPCCLLENNGYYYSNLNQEHMLMNNNSMINGYGMEGLNLQEPTTVMESYEMLPFDYYQPAADSEWTVGNMADTLWNMDAL

>Maker00000043 EpbHLH1

MKNQKLGDAVITDTDPIYVLYMQCEPILPACLAMNTRLPGWNFETDAFVTNQKKPIGPDQELVELLWQNGQVVMHSQTQRKPVVNSMDSRQFQKNDQSIIRTNESYGNSSHLIQEDDAVSWIQYPLEDPLEQQFCSNILSELPSSGVESDKPIRQVEEGKYMKFGPYGTPHVTASSQTPNIKPFHAQEFSGNPMPSPRVHFPDSTQKNISFGGSQNVLNFSHFSLPSKVASTYSNFQSVEKGNGNMLQNKAKECSVMTVGSSHCGSNQVPLDPDASRASSNGVWTTTLYAEPGPQMDDVQKSIPQCEKGKSEVLEPTLSSSSDGSGSLAKTCSWSTRSLRQKRKKMDTEELEEQSEATEPKSAVENKVAQGTRSSRRNRAAEVHNLSERRRRDRINEKMRTLQQLIPHSNKTDKASMLDEAIEYLKSLQLQLQVMWMGAGMAPMMFPGIQHYMSQMGMGMTSPSFHPIPNPMQLPRSSLDQSISVAHTPNQGLMRQTPVMGPFTYQNQMQNQALSEQYARYMGYQFMQSASQPMNVFGYGSQLQHSQAMIPPSSSSGPLNGDANNNDTLSGKIG

>Maker00001432 EpbHLH2

MGGHENAMGFQHRNESILSCPLSGLGANVSDLSNPFLGSSVWDPLVSLSQTQSFGGSSLVSHSEFANSSYPIVLEHQGLSGSSRLVPYISESNFADMITKIPSYGNGDTANAGYESTYNPNKEADFEKIAINEAQLQGEHSIPEEGATTSALIANRRKRGLGYNSTLNPNKVGRSAEGEALKGSLATSSDGVEEQADKKPKLDPNTSADLRGKQSVKQAKDSPHSAEAPKENFIHVRARRGQATNSHSLAERIRREKISERMKLLQELVPGCNKITGKAVMLDEIINYVQSLQQQVEFLSMKLATVNPELNLDAERILSKDMLQSRIGHGALGGYGGSIIGSGHLFSSGNLLGGLGSIPSISPNIPSLPPSIMDQELRNIYGMGYDSSTPLQNLGPNGQ

>Maker00000880 EpbHLH3

MELCEHDHFLEELLALRRETWDTIPSESNQVFSNGWGFDCVDQNSVPFLPNSSCQEIPQGYHNNYAFNEIYSSLLDEFPAPQNVDSSSSSYNVLDAPFLGQEDHPLSVMEELEDPSLLGEELQSLDLQTPCKIEPSQSPEMPVFNMGDASLERKNRAKKLQGQPSKNLMAERRRRKRLNDRLSMLRSIVPKISKMDRTSILGDTIDYMKELLEKINKLQHELEEDSDMGGIFKDAKQNEILVRNTPKFEVKRRNEDTQIEICCAGKPGLLQSTVNTLEVFGLEIQQCVISCFNDFTVQASCTEVHYSQDYALSLSLSLYIYIYIYWCYVLMLLSYLSRNRNWSRGQY

>Maker00000166 EpbHLH4

MIMALEAVVFSQDPLSYICCKDLYSLAAAWNHNDHAEDKVPVGLISSNTAEPRHVHATWDSKSFPEACTARPSLVSPAPSGRRKRRRTKSSKNKEDIENQRMTHIAVERNRRRQMNDYLAVLRSLMPPSYVQRGDQASIIGGAINFVKELEQLLQSMEGRKKTKQREPECSSPFSEFFMFPQYSTHANHSNTSAQQRAVADIEVTMVDTHANLKVLTKKRSGQLTKMVVGLQTLRLTVLHLNVTTHDDMVLYSASLKLEDGCELNTVDEIAAAVNQLLRRIQEETPFC

>Maker00000716 EpbHLH5

MDDSFSDILEDKEFGGDDLFAILESLDHFSDFPPIDGAVVSTKENEETSRLVSQKSTSSSALQEPEETELEASPKSKRQKLALTTSLEDPNPDGQQRISHITVERNRRKQMNEHLSVLRSLMPNFYVKRGDQASIIGGVVDYINELQQVLQSLEAKKQRKVYSEVLSPRLVSSPRPLALSPRRPPVSPRLSLPISPRTPQPGSPYKPRLQQGYLSPTISGSLEPSPTSSAASSSLNDNINELVANSKSAIADVEVKFSGPHVLLKTVSPRIPGQALKIISALEDLALEILHVTVSTADEAMLNSFTIKIGIECQLSAEELALQIQQTFC

>Maker00006491 EpbHLH6

MACNSSEGLGDDFLEQILNVPPSAYANSESSPLPPMVLQLGSAGSAAAGGGGLRESLGMGMGMPLGLNLEQGFLRQDGNRVRYRDDAVEGNNVTNHHLHLNNSNNNGATSSSSTSGISERESVQRITGLFSAFGPLHLRPTTVRSPPAPSHLPHQPFHSQPQNPVSAAAVPQPPAIRPRVRARRGQATDPHSIAERLRRERIADRMKALQELVPSANKTDRAAMLDEIVDYVKFLRLQVKVLSMSRLGGAGAVAQLVADVPLSAVEASQGVEGGGNLQAWEKWSNDGTEQEVAKLMEEDVGAAMQFLQSKALCIMPISLASAIFRTSQSDAYTLTKPESNSHS

>Maker00007179 EpbHLH7

MVLEAVIYPPGDPFSYGVKDLWDHSLHQHQHQHPSLDFLQNHHLYADWDCSSPPHFNDLPLSNPSSSDANNPTISLPSEPHHLDPSISMPSRPKRRRPKTRKNQQEIENQRMTHIAVERNRRKQMNDYLSVLRSLMPDSYVQRGDQASIIGGAINFVKELEQRLQFLGSQKETEGTWESSPSAAKLPFSEFFTFPQYSTTSSSGCDSNCRPGREGVGEAIQSSVADIEVTMVESHANLKVRSKKRQKQLLKIVSGLHSMRLTILHLNVTTTGEIVLYCLSVKVEDDCKLSSVDDIAAALFQMMNTIHQEAIFG

>Maker00006380 EpbHLH8

MLSSLTTFLIETLSIHLPPSVLLYLAVSRYFATAMEREMHPTAHVVPWVGGEVEDEASWTRTNNEAEFKDDDIALGGPLTSFKSMLEGDWYMNSAINLAPQDLLTLPSHQETADIVFCSNPADSFLVQPLDSSSSCSPSFTLDPSQSQHFLQPKPCFPSLLNVVCDNPLDDGFDLGSDSGFLGPFRGHHSPSSSVLMNYPGLNTQSQMGNFELSSNSEFLATRLLPIADNTVTLCGGFCPAVSGGFDDPGGAPLLNRPKVLQPLEVFPPVDAVPTLFQKRAALRQSSLGADRLGALEISALRFEASSTGLEDMERKRKRVLDSEIEEDGIDASGLNYDSDELNENDKAEENEFNGGINSNANSIVTGGDQKGKKKGQPAKNLMAERRRRKKLNDRLYMLRSVVPKISKMDRASILGDAIDYLKELLQRINDLHNELESTPPSSLPPPSTSFHPLTPTPPTIPCRVKEELCPSSLPSPKNQAARVEVRAREGRAVNIHMFCARRPGLLLSTMRALDNLGLDVQQAVISCFNGFALDVFRAEQCREGLDVVPEEIKAVLLDSAGFHGMV

>Maker00007307 EpbHLH9

MQQQMDNSELYQFLAGNGSFFSPAPYGLPPAAASSMHSFCSSSYYPMDSSEITADHTPQQERALAALKNHKEAEKRRRERINSHLDRLRTLLPCNSKTDKASLLAKVVQRVRELKQQTSQITELETIPSETDEITVLSTSSDYAGDGRLIFKASLCCEDRSDLIPDLIEILKSLHLKTLKAEMATLGGRTRNVLIVAADKDHSIESIHFLQNALKSLLERSNSSERSQRRRVLDRKLIV

>Maker00008292 EpbHLH10

MDSNSLPTYQHQHQPNSGLLRFRSVPTSFLANFIQPLDSDVDKASNPWETSESEKLVSRFVNNNDMDSSRYQEFEAKAPTEATLDHMSSFNGLPPHYPRQNSTASSAMDSSYGLAGSLGMDHQTQPKSFTSNLLRQSSSPAGLFSNLSYPNGYSTIKGVGDYGGANGNNGEGSPFMSKLKNPFSFLSRSGSALGMLSQISEIENESVEATNPDNGRLGSMNGYGSGFPYSSWSDASHISENLGGLKREPSNYEKLFSNAQFSFLTQISCDAQNGPLGNRVQILSHHLSLPKPSGDMGALEKFLHFPDSVPCKIRAKRGCATHPRSIAERVRRTRISERMRKLQELVPNMDKQTNTADMLDLAVEYIKDLQKQFKSLNEKRANCKCMSMQKADASDVV

>Maker00006851 EpbHLH11

MVTSSRAAPLRRSSMSYCVPDFEIEMDDVEYPIPTSSGVTRQKKPSVSEDDIMELLWQNGQVVMQSQNQRPQRKPPPERYTDSVIPAGPSTVREIRPSQQSEQHYQNQHIFMQEDEMISWLHYPILDDPPLDHGFCADILYAQPPTVNNSTMQNPNRTSQATELRQLPRSVAPRPPIPPPRKPEQVQTMIPNFAHFSKHNARGKPAGPSSSKTAMMESTVVDSCDTPAVMASASRVSGTFRNSAEPTERDAAPGTMSTASGAAPSTTSVGGDTSIREATTCEMMATSSPGGSSGVAELASRPPHEDRKRKGREPDDTEGHSQEVYVESAEAKKQICGSSSTKRSRAAEVHNLSERRRRDRINEKMRALQELIPRCNKSDKASMLDEAIEYLKSLQLQVQMMSMGCGMVPMMFPGIQQYMPAIGMGMGMGMGMGMGMEMGMNRPLMPFPNMLAASAMSPTATAHLGPRFPVPPFHVPHAPQPDSSRMQATAPNQSENNMLPSLVTPDPSQSRIPNFTDLYQQYLGPHQMNLQFMQNQAMSNSNVSKSSTSKSHENAEKTQPVRLEIQNARGNTRSGTRKEGRRKLCLGHKFANGFHESDSNI

>Maker00006653 EpbHLH12

MEGQSNRENPPKMMPFCSGGSSNHAYHSAENVAAAVGKNGNESQGTCLPEPYHNYVEYLREASPYLLNSSAEYVLGSCCPHVGSFYNFNEVNRTDRDFLYNDPGKAFCLKATDSSAGIDFRKVGLWRPDREGQQSKKFATNKHAESQAPRKGESSGDKHSASKLDPIVTGGAPGFLPKSRSSSSKQKASAADRQRRQRIADNLKALHDLLPTPAEESPAYILDDIIDYVKYLQLQIKELSGNRLQGELTAAMPLTFREGYGHYINHQMLNEPLEEMIGKLVEEHPAAAAQLLESKGLFLLPMSLVEDLRQAMQMFGGASPV

>Maker00006977 EpbHLH13

MSSRRSRQQSSSASSSSISDDQIIQLVSQLRQVVPEMRDTRRSNKASASKVLQETCNYIRSLNREVDDLSQRLSQLLATIDADSAEAAIIRSLINP

>Maker00007945 EpbHLH14

MAEFTADFQNLRPSFPFLEIDPSLELINQFTEMMSPLVLDNSSLHLQGLIPFYQEPDFPGNVEGAFPVFPADNEIHGSQKRKAMDATETSSVNPSPAVSESGSKMSSGRGKRAKSNVTEEDKTKEVVHVRAKRGQATDSHSLAERVRRGKINEKIRCLQSIVPGCYKTMGMAVMLDEIINYVQSLQHQVEFLSMKLTAASTFYDFNSETDAIETMQRARAFKAKGLSRFGEEGYGGGVSCFQPAWPL

>Maker00007279 EpbHLH15

MIIPLLVSEILIKKITNTSTGNKLSEGRNNPNEHRKKKMTHKEIERQRRQEMASLYASLRSLLPLEYIKGKRSISDHMNEAVTYIKHLQKNIKELSAKRDELKKHSDSNSDTSENQQKSMNFTVNENNGAVGIEIISNFKDERFPLSKLLQLLLDEGLEVVTCLSSQVNGRMLHSIHCEVIKT

>Maker00024558 EpbHLH16

MARSAIGQQEELEDDDEDHLTGNGSSSHRVKVDEATREQRVNAHRSKHSETEQRRRSKINERQAKRLASTFILCICPCEIPCVVAVLTGLELIEMQLIGLMLKTDLSAHPCIPGVESPARFQILRDLIPQNDQKRDKASFLLEVIEYIQFLQEKIQMYEQSYQGWNQEPMKLIPWRNHREPTENITSQAIQNGSSHEHNDVFSPSLPLNAQNTMESDLSSTIICKAGPPGLASEAVPLSMQMQLDMFEPTVSSIPTQHLHEVVSNTENTPPHTQPQVCLSRPKEVTCDFPNNNLKEQDELTFESGSVCISNAYSQGILDTFVQALRSSGVDLSEANVSVQIDVGRRSIASTSSSKDHENRFVNNPAVPCSGVDYCSEDSEQALKRFRTASS

>Maker00019453 EpbHLH17

MEPTSEGGGGRLSRFRSTPASWLEALLKEEDEEDPLDTSQCFTQLLATGTDNLSKNRASFDSLAVDGGGFDAGSSLAGFSRQNSSPAEFFENSMVATETGYLSKFGVQANHDASPQDIDVSSMASFNPIRQNVEHSGKAPDMDIDKILEDSVPCRVRAKRGHATHPRSIAERVRRTRISDRIRKLQELVPSMDKQTNTADMLDEAVAYVKSLQKQIEVLLTCIVFVSCPSSSFKDIGVYLSYDCYLSSLNWNLQSNNRNVNAQFKSKLAVAPMECLPCLCNSEISYSCLDEDHSRSSAFGDISSFSQTISPGRFAVCDTFFCKI

>Maker00014243 EpbHLH18

MALPMYTYFNWDTSPQYSIRDPEMSSYSSVFQEETPTPPPPPPPPPPALPDYSDHESLLFLDNPNSGLFDYYYYYSSFPELTYPLHPQYQTPKLPSDYSIFPGEEQIVHPYPKRQKLCSEDINYHLPYHNDNYYSFDGFDQQSNNHPYYSSSLMPEFNISEVQPLQLPAPAPAPAPAPASDCCEKKMCGGGEAKATVSAQSVAARERRKKIAEKTQELGKMVPGGCKMNTAEMLLAASNYVKYLQAQVSMLQVMNTLTDDKASTSSDYQALLGCPCLEEKLYLQEKCLVPQHFVTTLTNHTDIQTRSSVIKDLNQLITVTETEKKQIQ

>Maker00014133 EpbHLH19

MEVDPCTLLPEFSTEKISSSSSLEVENTENQKEIIDHHRLADQTLYHQPETNSTAPFSFSEACLSFSHLDGNLGHDAEKPEWKICTDWIEFDAAMNNGQSEGHKWRSKKTPLPLPHHIVVERKRRQDLNERFLALSRVVPGNKKKKDKASILGEAVNYMKDLQERIKNLEEKNKKRVMESVVFVKRSSSSSSPYSNQYLPHYRNNNKINNKGGYCNYPTYEESDDDNFSNKRSCLLLLPEIEAKVIERSALIRVHCGNDHKAIFPTLLSAVSKLHLIVVTANAVPFGNHTDITVISQVYISYHIINTL

>Maker00003730 EpbHLH20

MVGPENTNWLFDYGLIDDITVPDESFTIPASGFSWPAQALNGSSNNSVEIDGTLGDSDGVKESGSKKRGRSESCAAPSSKACREKLRRDRLNDKFLELGSVLEPGRPSKTDKAAILVDAVRMVTQLRGEAEKLKDANVKLQEKIKELKAEKNELRDEKQRLKAEKEKLEQQLKAINAQPGFVPPPPAIPAAFAAQGQAPGSKLVPFIGYPGVAMWQFMPPAAVDTSQDHVLRPPVA

>Maker00003826 EpbHLH21

MEGFDWSSGTCGNTPDRSSHGFTVNPGVLESGFASSSSLVLDSQIGELLEASVKDRNKGVSSERSIAALKNHSEAERRRRARINAHLDTLRTVIPGANKMDKASLLAEVIRHLKELKGNEAEACEGLMIPKDSDEIRVEEQEGGFNGFPYSIRASLCCEYKPGLLSDIRQALDALHLMIMRAEIATLGGRMKNVFVMISCKELNEEDAEYRQFLAGSVHQALKSVLDRFSASQDLLGIRKRRRISIFSSSTPGEFW

>Maker00004083 EpbHLH22

MDNNFVTAHSGSDDASASSLDPMFSGVSLQSILSLNHSVFSDYTAGAGLPSLAFLSEDSLSLNMQGEVTHGSNIGDHMPETKEKKPILVPKSEPCLFEDIANGGTTSHPFDFFSNHCPPNFYDSSTFSLLPQLHSIESFSQKRPRLDSLISAAGAMPALAQHNLHSPSVRSDIFPVAPPYNSKPSSLSDAPIGDKSSPLIPQSTLARQRRLKLSDKTRCLQKLMPWDKKMDQATLLEEAYKYVKFLQAQFNALQSMPSAPFLSQCGTSLSQLSVQNGEVFGDLERLNRNQVLQVLVNSPVAQTRLYSQGFCVFSVEQVTLLNNISGRRLLLQQMLSDNASSKSLLN

>Maker00003914 EpbHLH23

MDIDSPHNSNWLYDYGFDISVAGAEFPVVPSGGFNWVSQTLNGSSDTSLDLEYSLDNSTVLENGPSKRLKSESCASGSKACREKLRRDRLNERFLELSSILEPGKLPKTDKAAILSDAVRMLTELRTEAQKLKETNDELLLKIKELKAEKNELRDEKNRLKMDKEKLEQQVKGINVQPTYLTHPSAAFTAQGQTASHKLVPFVGYPGIGMWQFLPPAAVDISQDHLLHPPVA

>Maker00004135 EpbHLH24

MDAHQDPLMQINDFELHDFIDDTNFDQFINLLRGDNEDSFCNFDSDLINGCLADNQFIPLPANPFECNAAVNAYDPSSTLSSFSCFDGEVKGGGGGGDEENDSSATTITTPETTLSGNAKQRPKTDRFKTLISERRRRGRMKEKLYALRSLVPNITKMDKASIVGDAVAYVQELQEQAKKLKAEIDGLEASLFAAENDQGSVQNPIKVLNNLFNYPICKKIMQMDVFQVEDREFYVKLVCNKGEGVATSLYRALESLAGFNVQNSNLATVSDKFLLTFTLNVKGCEPEINLPNLKLWVAGSLLNQGFEFITTFHA

>Maker00004076 EpbHLH25

MESDLQHHLPLFRNQQQINSGFTRYRSAPSSYFTDIIDKEFYEHVFNRPSSPDTERVFSRLLSSFADDPPTQTLPPVTVKQEVCQNQQPQPQAIPSIINDHSLLRPLQNNMTTFDPAPRDSYKSSARPPLPNQSLHSANRLPPMKTGLGTTSNLTRHSSSPPGFPSATRGMRTLGAGDNPTELANVPAARTLKNQPTYSSGLMSSTAEIGDKNNAELENPQNEAFGESQGNDFLSGFPVDPWDDSAIMSDNITGLKRYRDDDDDDDAKPFSGLNAPETKNERGSQGSGPSPLTHHLSLPKTAMEMAAIEKLLQYSDSVPCKIRAKRGCATHPRSIAERVRRTKISERMRKLQDLVPNMDKQTNTADMLDLAVDYIKDLQKQVQTLTDSQSKCTCLLRQHQ

>Maker00018412 EpbHLH26

MMRDHMLDSWRLHWQKALSKFQWQGIIEDPAFLHQWNLNTVVDPTLLPLAAAFGETLQQHAFSNPTFYPKTSMEATLTGIERPLKQHKPNNWSPNTSDQNQTLDTDYDSCPNLLSFVNSNYMNQLGTLKPKEQTTCPEMNSIIPSDLLVSHGSLGNQNFVFNACQDPRKIGTTPKISQTQDHIIAERKRRQKLSQRFIALSALVPGLKKTDKASVLGDAIKYLKHLQEKVKALEEEQSRKSTVESVIVVKKTQLSADEADCSSDSADPCEEPLPEIEARFCQRSVLIRIHCEKRTGLIERTVSEIEKLHLIVTSSSVMTFGSSALDITIIAKMDMEFCMTVKELVRSLRSAFESFM

>Maker00018008 EpbHLH27

MAEEFQAGICGGNWWNVNTSSRSVFPLGSSLLCSVAANNNGGSYSGTWQTDPFVDLKLPIRSCDHEGLISDDHHDSLGFLVEQKPKQNESSSSASENGSILMDSTLQFMGFGLPSSPTTLLGRPESNFHSVLEEEAGVDSSNSEMIQKGQVSSIKEGFKAMTQEFSIDQQNLSYGYGLYDENNLLHPHPQPQPQPEDSLFTNPSMTYSYNSSNEVSPSWSKASHFLKPYSEPKQQLGGFHFSNNNKNPFWNNASAEALDDGPPSPHVFASSPPSQTFQHKPTNCSSLITKHKRDEAPVSEPAFKRPRMETPSPLPTFKVRKEKLGDRITALQQLVSPFGKTDTASVLHEAIEYIKYLHDQVSVLSAPYLNNRPPVQQQQGCDEKESKEAKQDLRSQGLCLAPISSTYPLANYEIATELWTHTYGGALMR

>Maker00018038 EpbHLH28

MMKNISASSPRSDRKVSERNRRSQMRTLYSKLYSLVPHQGMNSLPDQVDEATNYIKKLQIKLEKMKEKKLSLLGIENEPDNSGVLRRNRAMIMGSKSPKIETHHIGSALEVCVVTGLDCQFIFNDFIRILHEEEADVVSANYSVVQDTVLHTIQCQVGECGNRAARISNRLKRFVNGSGAF

>Maker00017451 EpbHLH29

MMQNIPPSSSSRKDRKVTERNRRSQMRALFLELYSLVPHQPSKELISLPNQLGEATTYIKELQIRLEKMKEKKLSLMGIDSNKTNTKRTLINDSRGMIMGSKSPKIEINHMGSVLEVSVITGLDCQFVFNDFIRILHEEQADIVNASYSIVQDAVYHTIHCQIGECGNRGVRISERLRRFLYGSDAF

>Maker00017733 EpbHLH30

MVYCSYNSNSSPGSDISRLIDPTSHTIVDSNGVSQSGLASPNSLVLNSEKEELMKSSAKIGKKGASEAKALAAMKSHSEAERRRRERINGHLDTLRGFVPCNAKMDKATLLAEVINQVKLLKKSAVEASRGFLIPMDDDEVKVEPYNHEGREGSMSYMASICCDYRPELLSDLRQALDGLQLQLMRAEISTLGGRVKNVFFFTCCNKEHMNIEACQLLPSAVHQALSSVLEKASSSLEYSLGMPLRSKRRRLCFIETSTSS

>Maker00017705 EpbHLH31

MSSRRSRQQSGSTRISDDQIIELVSKLRQLVPEIRDRRSNKVSASKVLQETCNYIRSLHREVDDLSERLSQLLSTIDADSAEASIIRSLINQ

>Maker00017698 EpbHLH32

MERHMLPRAPSVPWIEEEVDDTASWTRTINEGESKDQDIALGGPLTSFKSMLEGEWYMDSTINSSHQDLPSLSVHQETRDIGFCSNPTDNFLLQPIDSSSCSPSFTIDPSQSQPFLQPKSCFPSLFNAACSNPFDNGFELGSETGFLSHFQGNQTSTSHVLMGFSGLNTQPQMGNLELSSSTEFSATRLLPVADNGVAFGGGFSPIGLDGYDGSGSTLFSNKAKVLRPLDVFPPVVAPPTLFQKRAALRQISGGIDKLGTLEISPSRFHDYSNMEKKRKLAEEGEIEEPSIDASGLNYDSDELNENNKVEENGNNGGSNSNANSTVTGGDQKGKKKGLPAKNLMAERRRRKKLNDRLYMLRSVVPKISKMDRASILGDAIDYLKELLQRINDLHHELESTPPCSLPPPSTNLHPLTPTLPTLPCRVKEELCPTSLPSPKNQPSRVEVRLREGRAVNIHMFCARRPGLLLSTMKALDSLGLDVQQAVISCFNGFALDVFRAEQCREGQDVPPEQIKAVLLDSAGCHGMV

>Maker00017378 EpbHLH33

MMLALSPSMFSPIEWALDEPTCHNNHNLMSKDAATALEFICSFDQVQVEQVQKSTPISLAISRDPTMVKKLDHNASERDRRKKINTLYSSLRSMLPFADQTKKLSIPATISRVLKYIPELQLQMEWLIKKKEELLVKISQQGDAASKESRRKMTHHDVATACVVSISRLNDGEAVIQISSDKLHKTPLSEILLCSEKEGLFLLNASTFETFGDTVFYSLHLQVDKTCRSELEILREKILSICENKERIFQCFQI

>Maker00018383 EpbHLH34

MEQTTVGLPGSSLAIRDHDKTALESIQFNQEIQEIMAPPPENASSFTALLELPPPQAVELLHSPDCGGTAATSSASGKSPCQINNHKPYLFHSFNGNLTFPANAALIERAAKFSVFAGENSHPEDSSLVAPSSGANLDKVKNEPPETDSNPSSMQGCVSDSAVENQTQRAAKRKEREKKVKGSSKKSKGVADEASAEAEKLPYFHVRARRGQATDSHSLAERARREKINARMKLLQELVPGCNKISGTALVLDEIINHVQSLQRQVEFLSMKLAAVNPRIDVNLDSILASEGASLVDCNFPSPPLMWSEIPVNGNRQQYQQQWQVDAFHQPLWGREEGNHNFITPDNSFLSYDSSANSASLHSNQLKMEL

>Maker00018400 EpbHLH35

MFALSPSVFSHIERGLGEHTSHNDQQYLTSRDVRNLDFICSSDQVQVEQAQRSTPISLATSSDPTMVKKLNHNASERDRRKKINSLYSSLRSVIPLADQTKKSSIPATISLVLKYIPELQQQVEGLIKKKEELLLRISRQGDAASKEPPRKMTHHNAATACVVSTSRLNDGEAVIQISSHKLQKTFFSEILLCLEKELGLSLLNASTFETFGDRVFYSLHLLVDKTCRLEQETLSEKLLSICEKMKGISQSF

>Maker00017672 EpbHLH36

MDLNSHQNYQHQHHHQSNSGLLRFRSAPSSLLANFRQAVESAGSERRISRFASNCNNCDTASPSFQESEHKPPKPPKEAALDSMNSQQGHSGTGLPPHYPRYSSSMDSSYGLMGSMGMDRETQPRSFDSPLLRQSSSPAGLFFNISFPNGYGAMKGVRNYGAVNISSNGELTPPINGLKNQISFSSRSTSLGVLSQISETGCEIGATSPDDGRLGTNSSDSRYYGPGFPYASWNDTSHVSENLAGFKRDETSNEKLYSDAQNVELGNRVNVLSHHLSLPKNPAKMAAVEKFLQFPDSAPCKIRAKRGCATHPRSIAERVRRTRISERMKKLQDLVPNMDKQTNTADMLDLAVMYIKDLQKQFKSLSEKRANCKCTSTQKADINRVV

>Maker00018558 EpbHLH37

MEEVLELARAKDTKERMAGVERLYELLEASRKSLTSSEVTSLVDCCLDLLKDNNFRVSQGALQALASAAVLSGDHFKLHFNALVPAVVNRLGDAKQPVRDAARRLLLTLMEVSSPTIIVERAGFFAWRHKSWRVREEFVRTVTSAIGLFASTELTLQRAILPSLLNDPNPGVREAAILCIEEMYAQAGPQFRDELQRHNLPSSLVKDINARLEGIQPKVHSSDGISGGFVTGEIKPLSFTSKKSSPKAKSSSRENSVFAGDGDAIEKSIDSIKVYSEKELIREIDKIASTLVPEKDWSIRAADYPCFRGLLKQLVGPLTTQLSDRRSSIVKQACHLLCFLSKELLGDFEACAEMFIPVLFKLVVITVLVIAESADNCIKTMLRNCKVARVLPCIVDCAKKDRSAVLRARCCEYALLILEQWPDEPEIQRSADLYEDLIKCCVSDAMSEVRSTARMCYRMFAKTWPERSHRLFPSFDPAIQRLINEEDGGAHKRHASPSIRDRGALVSLTSQASAPSNLPGYGTSAIVAMDRSSSLPSGTSAKSLGKGAERSLESVLHSSKQKVTAIESLLRGLDLSNKRNSAFQSSSLDLGVDPPSSRDPPFPAVVSASNHLDSLTTESVISGVHKGSKRNGGLGLSDIISQIQASKNSSKASYHSNASFEPLSALPSFSSKRASERLQERNSVDDNGNNREARWFMNPHIDRQYSDAPYRDANIRDPQIGYMPNFQRPLVRKNVAGRISSGQRRSFDDSQLSLGEVSNYADGPASLHEALNEGLSSGSNWSARVAAFNYLRTLLQQGPKGIQEVVQNFEKVMKLFFQHLDDPHHKVAQAALSALADIVPACRKPFESYMERILPHVFSRLIDPKELVRQTCSTTLKVVSKTYSTDSLLPALLRSLDEQRSPKAKLAVIEFAISSFNKHSLNPEGAANTGILKSWLAKLTPSVHDKNTKLKEAAITCIISVYSHFDSTALLNFILSLSVEEQNSLRRALKQYTPRIEVDLMNYLQNKKERQRSKSSYDPFDVGTSSEDGYVGLSRKTNILGRYSAGSVDSDGSRKGNSLDSTLVKASFCQAASGENQEHSYQNHEIVSTSGILGSKTKDLVYAVNSMGQNFGSHDSQLGHSDSGMNFEGLSTPHLDVNGRISLEHSNLNVSEGFGREKEDPPELDLDHHSTEAVKINSSKYVEPGIPQILHLICNGGDGSPISIKQTAFQQLIEASAENDHFIWIKYFNQILTFVLEVLNDSDSPVRELALSLVVEMLKNQVSNEAEHCLTIVLSQYDPFRCLSLIGRLSQEELMTQIPSFLPALFEAFGNQSADVRKTVVFCLVDIYIMLGKAFLPYLQGLNSTQLKLVTIYANRISQARTGKTSNAEGLKRVKKMSTTSPDDGIMELFWQNGPVAMQTQNFRPLRKPQPPNHSGHVIPSGPSSATDIEIRPSHQAENYNNLNQHLFMQEDEMVSWLHDPILDVDHNFCANLLYSPQTPVSNSSQITDSTITQTDVRTSQATGLLQPSTSVPPRPPILPTWRTDTKIHEPVYFSKHNTRVEPGPSSSRTAVRGTTVVDSCATPAAAATSVASEAGRSSAAPTEGDACHMSISTAGVAAPSTTTAGEGTKAMEPEITAASSPGGSSGSAEQSDRSPTEDRKRKGREVDDFERRRDRINEKMRALQELIPQCNKSDKASMLDEAIEYLKTLQLQVQMMSMHMGCGMVPMMYMGMDMGMMAFSNVGPSSLFPHQMPFPFIQNQTMIRQNISEPSTSKGHQDPQDHHSGYAFYHSFQASKLFSTVLYPYSSIRFRFLFLSSELQNSQLNFNETRRFSAFQKLSGIEGLSGLNLMILIGISLEAITWFDTKETRTN

>BGI_novel_G002352 EpbHLH38

MAEKLCANEEDKTILESVLGAPATAFFNSAVSNNVFSDLIVPSGCDLGLQARLSQIVEVSKCTYAIFWQVVGLKSGGSALVWGDGFCRDHKGGDFNGAGKEKDEVKSRVLHKLRACFGGSKEADSARSDRVSDLDMFCSTSMYYIFGFDSRYGPGSSFKSGKSIWAWDFSHCLNQYESRSFLANSAGFQTVAFVPLKSGVVELGSKEVMPEDFNILEMVRTTFGEPSSGQAKAFPKIFGRELSLGDSKSQSITISFSPKVEDDSGFTSDSCEVQALATNHVFGNSSNGSIADSNEAKLFPQLNQMIGGNFNAQARVSCLDLGNEDLSPHPDERKPRKRGRKPANGREEPLNHVEAERQRREKLNQRFYALRSVVPNISKMDKASLLGDAITYITDLQMKIKVLEAEKGLMNKDKQVPLTDIDFQARQDDAVVRVSCPLDTHPVSGIVKTLREHQIVAQESSVSTADDKVIHTFSIKTHGGESAAVQLKEKLEASLFKD

>Maker00018521 EpbHLH39

MSVPLITSRLQSMLQAAVQSVQWTYSLFWQPCPQQGILVWADGYYNGAIKTRKTVQPMEVSAEEASLQRSQQLRELYESLSAGETNPPTRRPCASLSPEDLTESEWFYLMCVSFSFPPGVGYVTGKSICQEAACMARRCKRSGQQNIFKSYSSKVALLCPLSPDFNDLNSFPHQSARIQTVVCIPLLDGVVELGTTDKVQEDLTFIQHVKSFFIDHQLQHHPPPPKPALSEHSTSNPTTSSDHSRFHSTPTPLALYSAVDPSPATPAALNDNLDEDDDEDDDEEADSESEAETARNSRPTSTPEASGAAAPTAAEPSELMQLEMSEDIRVGSPDDGSNNLDSDFHLLAVSHPTDQERRADSYRAESTRRWSEPVLQLPASEGAPLEDLTQEDTHYSQTVSTILHNQSIRWAESPSVDYRTYSSQSAFAKWNVRTDHIQHHHLHLSADGTSQLLLKYILFTVPFLHAKYQDDNSPTPRDATDPATRLRGKGTPQDELSANHVLAERRRREKLNERFIILRSLVPFVTKMDKASILGDTIEYVKQLRKKIQDLEARNRQMEADQQSKEQQRSLERVGSDKRKMRIVEESGGIRKPKTVEESPPPSPSGVVTSVQVSIIESDALLELECESREGLLLDVMQVLREMRIEVTTVQSSLNNGVFVAELRAKVKENANGKKTSIVEVKRALNQIIPHND

>BGI_novel_G002362 EpbHLH40

MGVSSQPLPVFLSMRNLPQGTEQDGNSSYGCFRRRVLAARGTKQKARRGAPRKVLVKRRARMIEGSRRRAMGINTRIRTLKRLIPNSYSESMGLDGLFRETAEYILSLQMRVRVMQIMVDVLTGSDD

>Maker00017791 EpbHLH41

MTKKTHAIRPDSSSSYSSHLASMYSKDFVELVWQNGQTLVQGGSSSRALKRPPCTAYSINPSHETYEEDPCIIKRARLNTLYSLLDSSHRNSQQTNDRCSHPVDLKYSERCSLGNKPRHEFSNSKAEGESSSMPTGGNTQLCSTSLQSQRHELGSDQTFGKINFPNFLRPALFFKSTYQGNSATRPSKGAVLARVQEFKATDSGSIKSLDQPGVIDSAKGSHSLKGVHPYQIAFTSDKANPTAPLDAEKDQETLPDEQSEAVGHDRVLKTQGSHGQYHNQIHGVKGKTAAEFSSDPLVASSSLCSLGASNDPTLRNRNHEDTDDSTFISDNEEETEDTMKETPAHEGSRAKKIRNTQMHNLSERKRRDKINKKMRALKELIPNCNKMDMASMLDDAIDYLKTLKLQFQILSVGSGVYMPLMMSMLAAAGGGMAMKPGIGIPSSIPPQFGNCSILPQVAPPHAHAIANNTLQMLPGFPNHLLPFQFPMSHPPFIPIPPKPLYPFLSPHLTPLHESDLNARLVSAMPASAQVCSFYL

>Maker00018245 EpbHLH42

MPLYELYRMARENLDSKDVNRTCATDQSSVFECRPENDFFELVWENGQIAMQGQSSRPRRSPISKSLPSHCLPSHNAKIRDKAVVNATDIRMGKYGDLESGLNEIPMSVPADEIDVSTDEGMTSWLNYTIDDFLQQDYSPEFMHELAGDAINEIPASDNYSLRDKRHNCNKGFRDCHRNPVRDVSSSEPGIVSRALSGGEVETTRTKASTGQLYLSSSQQCQASFTSIRSRVSDITENNTSNATQHSPSGETAQVPSAFGGFSSIKIQKHDPIMPSTRSTSTIINFSHFARPAAIMRANLQSKGKPSSVSLARSESMVHVNKDAVATSSNPPESNFINSRSELPGVSNMHRQQELGSSKIDLKPLELKSLEQKGATSEQSDHACNKDTSKDDQTPNQVLVESGAKGPTACGKSTEQVVASSSVCSGNGVEMFSDYSNQNLKRKSRDTEESDCHSEDVEEESGSVRKAAPARGAIGSKRSRAAEVHNLSERRRRDRINEKMRALQELIPNCNKVDKASMLDEAIEYLKTLQLQVQMMSMGAGLYMPPMMLPHGMQHMHSPISPFPPIGVGMPMGLGMGYAMGMPDVNSGSSRFPMIHVPQMQGAHLSVPPMSGPTALYGMARTNPQVFGLSGQGLPVPVPPRAPVIPLSGAPFMNPSALGLDASGTTGIVENMDSASASGLKDPVPNVNSQVMQGSGGCNPTREMSTQCEATNVGQEESALVEDNSGHASDADGKGVVNPGKEDNL

>Maker00017263 EpbHLH43

MCLPEGERDSHTSMALEAVVYPQDYPFGYGGGKDLYPHCHWPYHDDFNIPKQQDLSFVGPPTQDYGLGDWTDICSSSPTSMLPRMNELHLPSHPSSDTTNNPTPSSEPHHADSSSSRPKRRRARTRKNKEEIENQRMTHIAVERNRRKQMNEYLSVLRSLMPDSYVQRGDQASIIGGAINFVKELEQKLQYMGGSQRNRGEIG

>Maker00008625 EpbHLH44

MAEFTANLHAFSSSFHLLDIDPSIELMNQCTGMNPHVLDNSNLNCQSLMPFSCGSFFGSPEPQFPRNREENSHGLVQHANHDAMPVLVPSFPVEDEIHESKKRKPMDVAETSSANSTPAVFESGSKLKNSSGGGSKRVKSNVPDEEKQKEVVHVRAKRGQATDSHSLAERVRRGKINEKLRCLQSIVPGCYKTMGMAVMLDEIINYVQSLQHQVEFLSMKLTAASNFYDFNSETDALETIQRAMASEARNLSRYGRGEYGGISLVQPTWRF

>Maker00008707 EpbHLH45

MDINQNSTIFTNVNNSTRNVDKMEMDHHLHHQIPVYSSCPRCPNLPVQITPSSSTTIPSSDVVLGEGGGEGGEGEELGAMKEMVYKMAAMQPVNIDPATIRKPKRRNVRISDDPQSVAARHRRERISEKIRILQRLVPGGTKMDTASMLDEAISYVRFLKRQIRLLQSQPSPTPPQFITNTLPNTTTRSDIHSDHSLFAQSDILHFPAAPTSAAAMAMPLGLGFSSVYNHES

>Maker00008597 EpbHLH46

MAAFSFQYHPFLVDSPFFSSAPIKMTAFTEQDHTTNPAPQFHETPFNITNQENSCFDQSSKLNMSDTEPSSLTKNQSPETSAVLDRLEPGEQVTQKVTPMDKKKRNRNRSPSSSPQSKEAVGEKNKKQKKSNNGEVKEGNKRKDEKKNLKKSPEEPPTGYIHVRARRGQATDSHSLAERVRREKISERMKMLQRLVPGCDKVTGKALMLDEIINYVQSLQNQVEFLSMKLASVNPVFYDFTMDLDSLMVRPEKSNCIASPLPPSVPQCSLNQVTTFADTTTMTTTTTTPSTGSQFWDVEDPRQKFLHPYGISNSLSALQGNKLDLSIPVGVREFQTTTRISEKEILMLERPKLGLESVLSVEGRRNWNWLPKP

>Maker00008967 EpbHLH47

MDPPLVNESTFSPANPSAYSLAEIWPLPGDHTAGPGLGLRMPSLGHNLGPFPDNSIHRDGSVDESTVTEQSGGGGGAAAVAARGNQRKRKDATSEDESSKMVSVSTANDLNDSSNKRIKLGVSKDENGLKAEAEASSVAGSKSAEQSTKPPEPPKPDYIHVRARRGQATDSHSLAERARREKISERMKILQDLVPGCNKVIGKALVLDEIINYIQSLQRQVEFLSMKLEAVNSRMNINPAIEGFPAKDLGDQAFDVAGMIFGSQAGRTYAQGAQLGWLHMQIGGGFERTT

>Maker00021634 EpbHLH48

MSGRKSCRASNFNENEINDLVSRLQALLPRLNRRTDSRVSVSKILKETCSHIKKLQKEVEELSERLIELMDSADTRDIDEEGLRRLLQH

>Maker00021296 EpbHLH49

MTDPLLPNSLLTPQHRQVDSSSHLHSLPPVPMSNYEVAELTWENGHPVMHGLSGLPTAHTKPTWSRAHDTLESIVQQATCQRQKSNFMVPQDHTPTSVSSTVAPSNGTTMPSENSGRAQSEQTLTRKRVQSDSGYLGTNGLEECEPSNGGYASVSATFCRSLKKIKTFEDDSPSLCGSENGDQEEDRDTKEEVGRSNSTRRNRAAAIHNQSERRRRDRINQRMKALQRLVPNANKNDKASILDEVIEYLKQLQAQIQMMSMRNIPQQMMMLMQQQLQMSMLTRMGVGLGLGMLDTNTTTAPRAAIPPPFPPLTVAAPAIAPSFMMPPFIQVQAPFPKPELSSAATTNSSVSSMPDPYSTLLSQSVNMDIVNSMATLFRQQQVRENDQAMGSLSQQRRGQGN

>Maker00021508 EpbHLH50

MIMQPSSGEMQQGINTSSSSSSSSMNSSQIPLHHQIQPPSSMHPQQPDLHDDFLDQMLSTLPSYSWPDLNPKPFWDLNPSASDDNDNAAFHYDDQPSLPSKFRDPHINPASSAPKSAAAEAVALMLQRQLLMSRTVGGAADSGLLQMPLSLGAAVNFEPSRNDVVDGSSFKSHIPAGEGTVQSLYNGFAGSMHDAHQASNQTQHFQHPQGQNFGGSGASTNQAPANGAPAQPRQRVRARRGQATDPHSIAERLRRERIAERMKALQELVPNANKTDKASMLDEIIDYVKFLQLQVKVLSMSRLGGAGAVAPLVADISSEAAGDCIQSSTARNSSANQTASSSNDTLAITEHQVAKLMEEDMGSAMQYLQGKGLCLMPISLATAISTATAHCRNPLIPAAASNHHPSGNAGEGPSSPSMSVLTVQSASAVNDGAVKDAASVSKP

>Maker00021561 EpbHLH51

MENFGDQCKFYWDDNVLFPTEEFESSWGLDGPISGYYDSSSPDGAASSSAASKNIVSERNRRKKLNDRLFALRAVVPNISKMDKASIIKDAIDYIQHLHEQEKSIEAEIMELESGKNMMMSNTGNNNMNSSFDHELDLPVLLRSKRNKTAQQLLESVSSRNSLIEVHELRVTYMGEKTVVVNLTCSKRTDTMVKLCEVFESLKLNIITANITCYSGRLSKTVFIQADEEEKDIVQIKIQAAIAAVNDPESPMSM

>Maker00003501 EpbHLH52

MASVWPITHPLHTPSSSSSSSSGFLMSDMFGLNLEPEGDREDDDDDDDEEELGAMKEMMYKIAAMQPVDIDPSTIRKPKRRNVRISDDPQSVAARHRRERISEKIRILQRLVPGGTKLDTASMLDEAIRYVKFLKRQIRLLQSPPPQHCSNMGSITTGNHNTTLADWPYTPNSLPASTSTAAITGMGFNAGDSSSGFNHEVISE

>Maker00018803 EpbHLH53

MMSRANGVAWMDETREDENSTTPSSWANVNANISIEKNQNPSHSGFVQNKDEMGSLCALKPMLEIDEDWYIANNGIHNQPDIIRDITFSPNFADQDSLLLNPVDSSSSCSPSSSVFNNLDPSQVPYFLPPKPTLSSLLNVVPCNNPLVHGFDVGCDVGFLEPQASSSSLLRGFTDFSSNNNQISLPNLCSVPQVPTSGMSQVTESSFAGFQGFEEGPGKPLFLNRPKILRPLESLPPLGAQPTLFQKRVAFRKKIANNDTGRDRGKSEIGEGNEKKRKMGSWEEVEDTSFDGSGLNYDSDELIEDNRTEENGKNGGNSSNVNNTVTGGDRGGDKKGKKKGMPAKNLMAERRRRKKLNDRLYMLRSVVPKISKMDRASILGDAIEYLKELLQRINDLHNELESTPSGSSLSTPASSFHPLTPTPPTLPNRIKDELCPSSLPSPNGQPVRVEVRMREGQAVNIHMFCGRKPGLLLSTMKALDNLGLDIHQAVISCFNGFAMDIFRAEQWKDGQDVHPEQIKAALLESAGFHGVV

>Maker00018846 EpbHLH54

MAAFSHYQYYLSFLLDSSSFVLSTFGEQPHINSTPHPHQIPSSFSNHETNCADNPTSRMTVSSGYNEPIVPEPHSAPHTSMPQVKLENGDHVTLCDHKKRRVNYRNGSSKEGTERRNKKRKKRNNGVVKEEEKPKAEEEQEQKKIPQEPPTGYIHVRARRGQATDSHSLAERVRREKISERMRMLQRLVPGCDKVTGKALVLDEIINYVRSLQNQVEFLSMRLASVNPMLCDFDMAQDTLTGTPDRLNSIASPLLSVPQCGTDQATDFVNDYVLDNSASVFLQGQRSNVFSSQDAVSEFWDAGDERRGFPNLCGFNTNLCSFQC

>Maker00018838 EpbHLH55

MRKSKRDSSLKVDRKTIERNRRIHMKALCFKLASLIPSHYLKNTKDMISQQDQLDMAASYIRNLREKLEKLKGKKEEAMKLKETNSNSGMENMFMGSNLPLLELRDLGSSIEVMLISGLNRNFMLYEVINVLEEEGAEVVSASFSTVGDKIFHTVHAQVKLSRVGVETTRVYQRLQELITPLKSWDKETKFGTQIFLEKIRVNSEGLDAEKNN

>Maker00019135 EpbHLH56

MSSRRSRSRQSSGSRTITDDQINDLVSKLQQLLPEIRDRRSDKVSAAKVLQETCNYIRSLHREVDDLSERLSELLATTDTAQAAIIRSLLMQ

>Maker00015442 EpbHLH57

MTPSDGTAASNSRDEMKFTRRFLRSLSKMRNPRPSSSSSLPSSSFCILSQFCFLFPFSPLALSLILPSTAQDSLLHMYQSSSSSPSQSQSGLTRYGSAPGSLLTNAVDAVIGEADREFSATALGSRPPVTHFFSGESSESSCKVNSSSPDPKDKPPRLGLQRSYGLHDGAAIGFGSSSSALLRQRSSPAGFLSHLTATQADNGGFSISRGSGSYSAQGVSNSGHGVSRLKSQLSFTGQESLSQISEVSENVVDGINSDNGHHRAAHSYATTSFGMDTWDSSNSIVFSAPPSKRAKNIDGDILNCLNALESQFSLPQTTLEMATVEKLLHIPEDSVPCKIRAKRGCATHPRSIAERERRTRISGKLKKLQELVPNMDKQTSYADMLDLAVQHIKGLQTQVQNSRIAPVDVNKIHSVCLDERLWRKKVDRVQGF

>Maker00015391 EpbHLH58

MEKDHDNYHSAPSFPQTFKAVQVDYSQSQYPLTHHHQNLMKFRIGEASGQSSHMNDNGMVDYMLHNNSTPSPQQQQQQQPCGFYSSSFDKLSFADVMQFADFGPKLGLNQTKNCEGEEDQSGMDPVFFLKFPVLNDRSLEDQNMMGTDEGENVQEDGVREDGLEGPVSDSNKSVQQQQQGLVGEDMEKNYSNPTSTVQENNTKSKRKRPRTIKTSEEVESQRMTHIAVERNRRKQMNEHLRVLRSLMPGSYVQRGDQASIIGGAIEFVRELEQLLQCLESQKRRRLLGEAHSRQVGDSSLAIQAQQPPFFPPLPLPNDQMKLVEFESGLREETAENKSCLADVEVKLLGFDAMIKILSRRRPGQLIKTIAALEDLQLIILHTNITTIEQTVLYSFNVKVAGDSSFTAEDIASSVQQIFSFIHENTGIFSNITGNEWGVGNVGMVAESPEDKILNSDYEEDDYELGSQRISYHVAFGVVDLSEMTLLEVLGFGLRETAIDGRVCQL

>Maker00015809 EpbHLH59

MSHITVERNRRRQMNERLKVLRSLTPRFYIKRGDQASIIGGVIEFIKELHQVVEALESKKRRKSLSPSPPITLDHQVDSPLGVGPADSFKDVGAISCNSSLADVEVKISGSNVILKVISHKIPGQVAKIIAVLERLSFEVLHLNISSMEDTVLYHFVVKIGLECQVSLEEVAVEVQRSFCSAAVTTAEM

>Maker00015735 EpbHLH60

MDSLEGGSCWDFIDYSLLNDSPSAPTDFLWSNQSASSENDFSGGAIVPLDNTRKRGRPNLSSNPGTKACREKMRREKLNDRFSELSSALEPGRPVRTDKSAILDDAIRILNQLKNEAQELKETNEKLLEEIKLLKAEKNELREEKLVLKADKERVETQLKALPVSPAGFMPPHAAAYPAGVNKMAVFPSYGYVPMWQYLPVSARDTSQDHELRPPAA

>Maker00015630 EpbHLH61

MDSVFSFSDAERLDFLQSVFHSYGCAYICLWSYSSSSNRLMFLDGFHNVQNHQASPSTATVDRWTFDQYKGLEFDVTSDDRIPGLAFRNRHSFLELQQSDLLRLASTDIQRQFFLGETQKDGKNSLFPEEFSRQPQPDRVEQKNPPPCCSSSLRSLSTGSPENTPHIFSNIPGGNLLPEMLQNEHDAIVRAFIHVISSPSSNNPSSCHQPNLPDVRSKEVSAFKGYRPSEMDSSKPNFPRQSLLKRSCQISRGLHLMRMREPMHQPIRHTSTQLLHVISERRRREKLRENFQALRSLLPPKTKKDKASILTAAKKTLESLIEEVKEIKKAQIKARELTCQETKTSSSNESLSVRVSHVVGSSSSEERFIDLHVTVRGQNHLHLVDIMIQLLEFLKGVQHVRLICVDANTHIAQGTAINEATLRLQIEGSEWDEPAFKEAVRRLVADLLMVNNIN

>Maker00016254 EpbHLH62

MENIGEEYRHYWETNRFLQTEELDSWGWDEAFSGGYYDSSSPDGAASKNIVSERNRRKKLNERLFALRAVVPNISKMDKASIIKDAIDYIQHLHEQERSIEAEITELESGKQMKQNSSDIDYEQDLPVVLRSKKKKTGQQLYDSVVTSTNSPIEVLELKVTYMGEKTILVSLTCCKRANTMVKLCEVFESLKLKIITANISCFSGRLLKTVFVEADEEEKDLLEIKIQTAIAALNDPESPMSI

>Maker00015546 EpbHLH63

MQPCSREMQGINSLLNSSQIPLPDLPQIQNSQMHHFDPSSHEDFIDQMLSNLPSSSWPDLNPKTFWDDTPPPHTAQNVAFQYDDQASLATKFRNHQISGDGSSPSSKSSTAALMLQAHQQLLMSRGIAGDSGLLPMPLSLGGGDVDRSQNDVMDRFKSPNPGGEGSVQALYNGFAGSLHGGGQASNQTQHFPHPQGQNLSSSGAATNQAPGGGTPAQPKQRVRARRGHATDPHSIAERLRRERIAERMKALQELVPNANKTDKASMLDEIIDYVKFLQLQVKVLSMSRLGGAAAVSPLVADMAASEGGGREGTASRPPPTAGPFLGAPTATKRRRQPPTTALR

>Maker00009651 EpbHLH64

MALEDHKSVLEEEDEFSNDSSISNHCPGFVYKVTSIHPEETQSLINFKANSGYNPVMNGGESLLSFQQREYCVWETNGLHQWSQISPRSTSSDPRLIQDFNCIQTASGYGKEKQLQVESSYGWPYSEPTVPFDNNSLQDSSAAEETSFHKRLSMGENMQASKKQCTTSVAASKTPKPTKPNPSKDPQSIAAKNRRERISERLKILQELVPNGSKVDLVTMLEKAISYVKFLQLQVKVLATDEFWPVQGGKAPDISQVREAIDAILSSQKDRSSTSK

>Maker00010626 EpbHLH65

MALEYHKSVLEEEDGFSIDSAISTYSPPVNHRIVCRAISYHLDEAQSLINFKVNGVAMEQRSSFKLRVHMGGSSAAKEASFHKHLSTGESMRASKKQCTTSGTASKTPKPTKSNPSKDPQSIAAKNRREKISERLKILQELVPSGSKVDLVTMLEKAISHVKYWQLMNSGQSNEGKHLISLKLKKPIDAILSSQKDMNSTSK

>Maker00009772 EpbHLH66

MENFYYSSSGSSWWPVQGTNSNGPSPFSVQTPLLPHHISESCSVQFCEFPHSWSAEDKAASASKSHSQAEKRRRDRINAQLATLRKLIPKSDKMDKAALLGSVIEHVKDLKRKAMDIGTSFTVPTEVDEVSIDQDESSSPINKVNYKVKGNNIVIKASVCCEDRPELFSELIKVLKGLRLTAVKADIASVGGRIKSILVLCSKDSDDSVCVATLKQSLKSAVTKIAASSSSMTSSCPARSKRQRFFLPSPCLH

>Maker00009558 EpbHLH67

MDDGFSDIFENKDLDLGGDDLLAILDSLEDFTNFPPIDESLTVCSKESDETTSALEPDTLAQLRTSPRTKRQKLVESSEEPKISHITVERNRRKQMNEHLSVLRSLMPCFYVKRGDQASIIGGVVEYINELQQLLQSLETKKQRKVYSEVMSPRLVSSPRLSPRLIPPVSPRLILPISPSTPQPGSPYKPQPRLPNSLEPSPTSSASSSINDNLNELVANSKSPIADVEVKFSGPHVLLKTLSPPIPGQALKIISALEDLSLEILQVTISIADESMVNSFTIKIGIECQLSAEDLAQQVQQTFSPHYHHNLASLIAF

>Maker00009898 EpbHLH68

MNGEDQLKSSTLGQDNQMDTMMMMMQLPDEFSGAYGNDAFSDNYALPELSAAATRTDTDIHGTMLHFMDNCPMTSSSSFTNLQSATISFTNPTTMVQEQSPPPCGGNAYVRDSEKGISVAAMREMIFRIAVMQPIHIDPQSVKPPKRRNVKISKDPQSVAARHRRERISERIRILQRLVPGGTKMDTASMLDEAIHYVKFLKSQVQSLERAAANRSPGIGFPAAMSSASSTSYLPIPSKPYQVPPQQHAGAFLEWGWLY

>Maker00010106 EpbHLH69

MSFFPTGVDDSDENTSIKEHRISPEDDPGDFICPVENAAEVLQTKRVPARTSSKRSRAAEVHNLSEKRRRSRINEKMKALQNLIPNSNKTDKASMLDEAIEYLKQLQLQVQMLMMRNGLSLHPMCLPGGLQSMTLPQTALNFEEPNRCQNSSRGIASSANAECLVQPDFSLAKHCSISDQPMIMPSMTNMANPDASSGFKFQVGQVDPSVSNGKRFIC

>Maker00009727 EpbHLH70

MESLGAFPDVEMSCFRGMFASEQHAHSPQFLGQSSLLLGEHDEMLIGIESAFCSAPEAGENECMFDPLDALNSDLQFISQGSSFSSNCSGDSFFSANPCHTNYCFSDTNQALVNDACMSMNVCMMHEKNTITVIPSSGDVLMEESVNFTGDGRGDKLESCDQSQAEFTAFPAVQVRLKRKFDVPEQEVSAEDKISINTSVNQKKKPRVSRDVQKRKKNAEVKKNQRVFGNGHEIEETNTGLEGQSSSSYSLEEDNASRENSGGTSSESKSIGTHNTNGKTRASRGSATDPQSLYARKRRERINERLRILQNLVPNGMKVDISTMLEEAVHYVKFLQLQIKLLSSEDLWMYAPIAYNGVDIGLNH

>Maker00009834 EpbHLH71

MGGPENAMGFQHGQETIMNCTSSGLSTNASEIAISSVSMAKPSVLANHFLGSSSTWDPLVTLNQTQTFGGSSMVTHSEFGGSSYPLMLENQALSSTSHLVQYMSDSNFAVPSYASGSFSEMVGSFPQHGCCDLANTRYAPSYNPNEEAAMKRVPLNGAQSQLEDSTPEGVTGSDPDGSGRKRGLDHNPTFSPNKNAEGEPAKDSSGKSSDAVKEQDEKRQKVEQNNTSADLRGKQSSKQAKDNSQSGEAPKENFIHVRARRGQATNSHSLAERVRREKISERMRLLQELVPGCNKITGKAVMLDEIINYVQSLQQQVEFLSMKLATVNPELNFDVERILSKDILQSRMTNGTIGGYGMNSSHSFPGGSFQGNLPGLPNTSSPFPPLPQSVLEHEFQNLYGMGYDSSTALDNVGPNGNTQIF

>Maker00010416 EpbHLH72

MEVSGHDFFEELLALRREAWDTIPRPDEENQLFFPNAYNTFDCFDPNSLAFLPNSFSQQVPQTSYNNYNSGFNEIYNSLLLDEFSTPQIIDSSSYTTLDTTTPLSTPPFLVQEENNPISSMVEEDVFFGEELQSLDLKTTCKIERSSQSPEMPVFNMSTCNLERKNRPKKLLGQPSKNLMAERRRRKRLNDRLSMLRSIVPKISKMDRTSILGDTIDYMKELLKKVEDLQQEIDVDSNMVGVLKDVKPNEIIVRNSPKFDVERRNVDTRVEICCAGKPGLLLSTVNTLEALGLEIQQCVISCFNDFTMQASCSEELEQRTILGSGDIKQALFRSAGYGGRCLMRENNM

>Maker00010345 EpbHLH73

MLPFRSFYGFESLLDHSAYSQEPSSSIKSMELDGGKSILASSSKSERKSMEACKSHREAERRRRQRINAHLSTLRSLLPNAVKSDKASLLAEVVRHVKDLRKQVDGVTRHHDGELSSSTISGESGSDHGDSEQWGFPGESDKATVSYCDGAGERKLMKATLCCEDRPNLNRDLEEAIRSVRAKAVRAEMVTVGGRTRSVVIVQWTDGEGEEVEALERALTAVVENRAMVGSELGRMSLGQKRARQYYRLPT

>Maker00009479 EpbHLH74

MALEAVVFPQDPFHYSYMLGHGYDLEAEQERGFAANWDYSSVLQNVKESWDCSNSSPHEALSPATSTGRRKRRRTKTTKNKEEIENQRMTHIAVERNRRKQMNEYLAVLRSLMPPSYVQRGDQASIIGGAINFVKELEQHLQSMQGQNKMQKLHSKHNPFAEFFAFPQFTTPATQSSTGCTATNDAVMGQNQKEAGVADIEVSLVDSHANLKILSKKRPGQLVKMVVGLQSLRFCILHLNVTTTLDDTVLYSISVKVEDESQMNTVDEIAAAVNQLMSRIQEEAAFC

>Maker00012568 EpbHLH75

MEAFDWSSGVRGNASETSPNGFLVNSSAIGSGFASSSSLVLDGEKGELVARPVKWERKGVPTERSIESLKNHSEAERRRRERINAHLDTLRSVISGAKKMDKASLLGEVIRHLKDLKRNAAQASEGLMIPKDNDEIRVEEQEGGSNGLPYSIKASLCCEYKPGLLSGIRQALDALHLMITRAEIATLGGRMKNVFVIISCKEHNIEDAVYRKYLAVTVHQAIRSVLDRFSSSEDLFGTRKRRRISIFNSSPSGGFL

>Maker00013277 EpbHLH76

MTSTTIVFPITPSFFEKPRTMEAQGNDPHHQPPPPIKVERKIIEKNRRNQMKILYSTLNSLIPDHNPKEMLPLPDQIDQAINYIKSLEAKVNMAKEKKESLMGHRKRSLVSSSSSVIEAKRTVLRSPRIEIREIGSSLEIALITGLDNQFIFYEIIRILHEENIEVVSANSSLSGDSMHHVAHAEIGQSLFQFGATKVSERLKRFVLGSASDVEMQPVFWGFDIASESWDF

>Maker00012497 EpbHLH77

MESDLEQHSSMLHGHQQQQLNSGLTRYRSAPSSYFTNINDKEFDEDMFNRPSSPETERIFSRFMNSFANEAEDSPAQHLSSVRQNSAVKEEMDQQSQVLPSMDNEPVALRQQHNSISNFVSAPQNLFQSSRRPPLPNQSLTSSAMEGAYSTGGHRLSCMKTSDANNSNLVRHNSSPAGLFSNINIESYAAMRGTGVLGAGSNSGEEANYSSAMRLKNPPKYCAGLMSSIAEIGDKSSTENNQDSESFAEGQGNEFITGFPADPWDDSAEMSDDVAGIKRFRDEDAKTFSETQNEAGNQPRPAPLAHHLSLPKTSAEMAAIEKFLQFSDSVPCKIRAKRGCATHPRSIAERVRRTKISERMRKLQDLVPNMEKQTNTADMLDLAVDYIKDLQKQVQTLSENHAKCTCSHKQRQ

>Maker00019830 EpbHLH78

MALTSYFNWESSLQADLNLEASIFHEEDPPEGEVLVAPDCNHQHHHLPHHYYYKSPLDPNTSYLDPCCSNLLYPHHDLLPCDPFIYENHYDSLLLPCPKRQKYLPYDQSGMEFITPVNGFFDGNSSNNNIVVPSVPEFYDEVGMGDIMTAESGGVVVEGGGGGGGRKKKEEKTVSAQSMAARERRRKITEKTQELGRLVPGGSKMNTAEMLHAAAKYVNYLKAQVGILQLINTLQEDEAFIPSEEMQKLITSPVVEEKMYMEEKCFIPTVFFTTLTNHKDIQSRPSILNDLNHLISSDIIKIKDD

>Maker00019908 EpbHLH79

MQTIEQNRQAYLLLRLSVPVCILSSDTDSRHSKISTLDFQNMSVLYSPSILNYPDGELRRNPEMDSIHHLNSGLLRYSSAPSSFLASLVQNSHGCFNEEASRRMDNHHPLHHHSSSSSSEMEDMFAKLSSSTNGWIKREEAGDSVSEGHSGYNSNYGSQMICPTQSQAQQVVQSLENGSLGSSGNAFNVSFNSTESKIRSNNLFRQKSSPAGLFNNFSVENGFATWRTVGSFKACDHVSNGQASTSASNLNDTSRAFSSRPYSCSRQLPDIAEDGNETLEEATCVEGRRNLRNENNGSNTECYVPSFTSDFWDGSSFNDSKIASDNDELMFSTSSGLETQNADFGYQNVGLTHHLSLPSSSTKMSDIEKFLQVQGSVPCKIRAKRGFATHPRSIAERVRRTRISERIKRLQDLFPKSEKQTSTADMLDLAVDYIKDLQRQVKMLTDTKAKCKCSSKPEAELSAL

>Maker00010982 EpbHLH80

MCFFLSRHYPISVSRTSAGEKPEMYEHTPFGPNTMADGAVSLKDSLPDAPPQLPSLIDPGNLTGCNNGLEENLKLTVEELSYHQHQNHPPLHAEDVSAFSNGIAASSLDVTHPQHLVLNVGNSNNNENLVQEVIEVLPYDHSAWDPKVQEFQDSGYSNHHHGQQQQTQQFQQFETQSYNSSSILDPPPPYPSSNLLDLLHYNPTQKIPSIHNSMGFLGDIPIGSDSTSSCSVLYDPLFHLNLPPQPLALRELFQSLPRGYSLPTSSRNGSLFGGGDEIEGNGIVYQDGDGSQLDNGVLEFDRGTARVGKGRQGKGTKHFATEKQRREQLNGKYDALRSLIPNPTKTDRASVVGEAIEYIRELTRTVNELKLLVEKKRFGMDRCKRQKPEDDAGESCNMKPFVDPEGCIRTSWLQRKSKDTEVDVRIIDDEVTIKLVQRKKISCLLFVSRVLDELQLDLHHVAGGHVGEYCSFLFNSKIYEGSSVYASAIANKVIEVLDRQYAAAAPHTSGSY

>Maker00023903 EpbHLH81

MDCTAARKTQKADREKLRRDRLNEQFVELGSILDSDRPKNDKAIILADTVQLLKDLTSQVSKLKEEYTMLTEESQELTQEKNDLREEKASLKSDIESLNNQYQQRLRIMFPWTAMDHSVMMAPPSYPYPLPVPVAVPSGPIPMHPPMQPYPFFANQNPGVIPNPCSTFVPYLAPNTVVEHQSTQYVSPPLPPGSHSHALGTQDSRNKPSRDSKAENKEGANDVTTDLELKTPGSTTDQELSSGQRKSSRLSMKGSSYTEGSSSGRCSSSHSVQDSSSSSVVGGRKADE

>Maker00023670 EpbHLH82

MLRCLNATGNCSDLTVLEKQIMRMKTQQEHQQYQQQLQYHQNQTFYRESALGEVIAKSIKPELAHLHYPCFRFGSSGFEVNSAISITTSMASAEDPRGKDHVSDKEKTSSACGKHSFKKRKSHKAHSSKVIAESETKEKKMKGSGEEGESKKTEQNSSKNTTNNNNKETCGGTSKSNSKASEVQKTDYIHVRARRGQATDSHSLAERVRREKISERMKYLQDLVPGCNKITGKAGMLDEIINYVQSLQRQVEFLSMKLATVNPALEFNLDDLFSKEVFSACGPSFATIGMGSDMNNPAYLQFNSGQQFPASIPETFLDSCGLAQILPTSTLEYDFHNLLNLGFDQARAASSSLSQPFTGKKIILC

>Maker00023552 EpbHLH83

MSDEEAHKQHQMMGLWENQSWGLSNSDEKLPSMDPPLIIGSSSNYNNNDPLIVDQKEEDEGINVEGDKTTSTSSRRSRGGRRTGRTAEEEIDGIGAGGGGEADDHEIHIWTERERRKKMRCMFANLHALLPQLPSKTDKSSIVDEAVSYIKKLENTLEKLQKQKQERLQQVQSASTTTFLYDPSANINSQWHPYESREAFMADQGASFHNSSSNNNLATTNDLNIGTSTSLSLPRIMNNPIGFQTWTSSNVVVNICGDQAQYNVCSLKKPGVLTSICFVLEKHKIEVLSAHVSSDNYRSFYMIHAQAIRASMANRDSNQFQEAFSVDESFRHAAAEMMLWLS

>Maker00006164 EpbHLH84

MEENPSSSRTDRKFIERNRRYQMKALYSELRSLVPHQSPREAIPLLDQLEEATNYIKKLQMNLEKMKEKKHNLMGTNQRSNFSKHREMIKKSSESPWFEIQEVGTSTLQVVLRSALDSEFTFNDIIRLLHEEGADIVNASYTVAEDAVFHSISCERKFLCIELPMCLERSSLQPCIQSSN

>Maker00005102 EpbHLH85

MDDAVEYSDRYCHMMSFDEQDEEFLRNILQQPASAKSHLSSPQSETESEFFSAINFSTAGSGNRGSTDENHVMSPKAYILSFDHSNVIPAPAAMSKQEESGTTELRSLKRIRETQNIEPRKKSRSSSEIENHIMAERRRRQQLTEKFIALSATIPGLKKMDKATILQEATRYVKQLQERVTELEKQNDNTKASTISSKNRETPKSDALYKTNEVLPEITARVSGNQVLFGIYCEKQKGIEFQILALLENFHLCVTSSSVLSLGNSTLGITIIAQMGDEYRMVVNDVMKALRQVLG

>Maker00005088 EpbHLH86

MDHAKKDFLDHMMMSFDEEDQEFLRNFLQQPRCDNYHLSSPQIKTKNEFSTAVVISTDDGSGSRGSSDQNHVMSPKAYILSVDHSDVIPAPAMSKQEQSSTSEPSSLKRIRTTHNIEPKKKRRSSSETVNHIMAERRRRQQLTERFIELSATIPGLKKIDKATILQEATKYVKQLQERVTDLEKQNNTNTEASMIMSKKCELYKNEDLHKPNELLPEITARVSENQVLFGIYCEKQKGIESKILALLQNFHLYVTSTSILPLGNSTLISITIIAQMGEEYKMTVNDVMKTLRQVVSKSREVDPY

>Maker00005131 EpbHLH87

MLSLSLQFSLQLYSAHENLEYTEATKRRLKTKFPDLAFSWMHILVLTAARSRTESSSMDGLSWDASHSVENLPILWSNMEQQELEASLDPSFPNNSMQEMQKQAQRGGHHLYLNYEKLVIKEMVQLSEAEAELGTTSNSKTSLSQLGGGTSLVGGPYCMNNPVCNFDPSEFNTAHQHPQLVNNCSQNDSFSTASLDCLFSATNSNTDPSVEDDGVSLILSDSRRSNLWNFGYHNGGSESESTTKSTNADHETLSQGSSDLYANQGIKIAESTEAKCLKRSIDDAFDQYPYFSIEEGGSSSFRLIKEKNNNNLPKPKRPRWDSRLPTSSNINFRQPHSSSSSMEEPDPDAIAQMKEMIYRAAAFRPVNLGMEVVEKPKRKNVRISADPQTVAARLRRERISERIRVLQKLVPGGSKMDTASMLDEAANYLKFLRSQVKALESLGNKVDAMNCPPTNSASEDERREGRKGFKDSQTFSIQKGQAIITPMIKIRALTTMLKTLSRKLLLENFCEKKERDI

>Maker00005387 EpbHLH88

MHLHWFSGFPVLTFYLCSDFHYPTSLWRSSTPSPLLETCIFFAFYGSFFGEHLEVNCLEPDFFSTEDEEQLYISSLEGEKMPFLQMLQSVESPQQFFPFAEANFQTLVRLQNLKKQPWEENVYVPRLETQIQAKELESCMTHDAIEIHSPVKSETNEDSHRLHHSASMVQKEPTIQAENSSRDPKRKKRKRTRPIKNKEEVENQRMTHIAVERNRRRQMNDHLSVLRTLMPPSYIQRGDQASIIGGAIDFVKELEQLLQTLEAQKRIRKNEEIGSPYGETVKPSLLSYDGEREEESGGKEEEEVKEAEIIEVTVIQTHVNLKIRCCKRQGQLVKAIVGLEDLRLSILHLNITSSQGSVLYCFNLKIEDECKVGSAKEIAEAVGDIFRRIDN

>Maker00005335 EpbHLH89

MTSCSPPPPPLLPPLLLPSPLPPSPTSSSSSSSLQHKLQFLLQAQPHSWLYAIFWHTSHDDRGRLRLSWAHGHFQASSRPRNHFFNVLDSLVDDNLNDAEWFYVMSLTRTFSLSDSSSLLAKAFSSTSLLWLNLTEHDFQFHDCDRAKEASLHGIRTLICIPTAHGVVEMGSSDIIPKNSTLIQQVKALYGSSDLIPSISPGNKQLEPVDFLDVEETVSFSDIGIITAVPDEEQDKTHADSRGKFRKKDHPHLSFRSYVDSEHSHSHSDSDGPMIEKAQPKKRGRKPGLGRETPLNHVEAERQRREKLNHRFYALRAVVPHVTRMDKASLLSDAVSYINELKAKIEKLESQLPREPAINNGSKKVKLEMVETVESTVDQSGPSGRGCRLGLGPCGLEVDVRIIGPDAMVRVQSENMNHPGARLMDALRDLELRVHHASMSCVNEVMLQDVVVKIPQGLRGEEGLIKSAILRRMDL

>Maker00005448 EpbHLH90

MKLELGLGGVLWNDEEKAVVASVLGNRAFEYLITSPVSNENLLMTIGSGESLQNKLSDLVDRPKVSNFSWNYAIFWQISRSKKSGDWVLGWGDGCCREPKGEEEEGRVTGLLSLRLEKDETQQRMRKGVLQKLHTAFGGSDEDNYAFGLDRVTDTEMFFLASMYFSFPCGHGGPGKCLASGKHVWYTDALSSDYCTIVLVPTDIGVVELGSVRIVGESLELLQVIKSEFSTQLSIPRVSPLSPLPAVVHEKRNENGVFSGVAVGGDKVEGVPKIFGQDLNSGNSGRPHFREKLAVRKMEERPWGGHSQPNGNCISFPNARNGVHVSSWAANQGGKQAGPADICPSKSPASNVPEVANRSTQDFRPSNYQPQRQVQMQIDFSGATSRLSVRPINAESELSDVEASCKEEQLAATDERRPRKRGRKPANGREEPLNHVEAERQRREKLNQRFYALRSVVPNISKMDKASLLGDAIAYIHELQGKLKIMESERERFGSTSSDAPASVADPKVENHNQATDVNIQAAQDELIVRVSSPLDAHPMSRVIQTFKDAQISVVESKFAAANDTIFHTFVVKSQGSEQLTKEKLIAAFSQESNCL

>Maker00004887 EpbHLH91

MKLELGLGGVLWNDEEKAVVASVLGNRAFEYLITSPVSNENLLMTIGSGESLQNKLSDLVDRPKVSNFSWNYAIFWQISRSKKSGDWVLGWGDGCCREPKGEEEEGRVTGLLSLRLEKDETQQRMRKGVLQKLHTAFGGSDEDNYAFGLDRVTDTEMFFLASMYFSFPCGHGGPGKCLASGKHVWYTDALSSDYCTIVLVPTDIGVVELGSVRIVGESLELLQVIKSEFSTQLSIPRVSPLSPLPAVVHEKRNENGVFSGVAVGGDKVEGVPKIFGQDLNSGNSGRPHFREKLAVRKMEERPWGGHSQPNGNCISFPNARNGVHVSSWAANQGGKQAGPADICPSKSPASNVPEVANRSTQDFRPSNYQPQRQVQMQIDFSGATSRLSVRPINAESELSDVEASCKEEQLAATDERRPRKRGRKPANGREEPLNHVEAERQRREKLNQRFYALRSVVPNISKMDKASLLGDAIAYIHELQGKLKIMESERERFGSTSSDAPASVADPKVENHNQATDVNIQAAQDELIVRVSSPLDAHPMSRVIQTFKDAQISVVESKFAAANDTIFHTFVVKSQGSEQLTKEKLIAAFSQESNCL

>Maker00002136 EpbHLH92

MSSTRTTHSISKLTSPQISSHKYFIKHMECSIPPTRNPTSSAASSSKAKKKNGKKSKGGVKLSTDPQSVAARERRHRISDRFKILQSMVPGGSKMDTVSMLEEAIHYVKFLKTQIWLHQTMINSVDDDCQSFPNHEQFTSLQQNPSSSSLGSVQHLTQTPLEQSCCFQSEVATTILDANMNYWPA

>Maker00002052 EpbHLH93

MENEFFLNAASLHFQPPPPSSMPTWQSFSSAMDVQASVPNCSSDQPQDCFYNNSKWEKSTDHHGLQFDSALSSLVSSPAASNSNIMPNNENFVIRELIGKLGNIGASPYINGSNSTDTSCYSTPLSSPPKMNMMNQLVKENLPSLGKSMLMNSDVAEFSSDPGFAERAAKFSCFGSRSLNGRTYQLGINNGQFPQRSTPLMENDKLARVSSSPSLKALGGSHMELANSQEESTISEQVPNGVNANSRKRKAPSKGKTRETSTSTNPTAKANETNEDSNAKRSKPNEGEGDEDGTVKMEEDSKGGTSNGGDEKQNKSSSSKPPEPPKDYIHVRARRGQATDSHSLAERVRREKISERMKLLQDLVPGCNKVTGKALMLDEIINYVQSLQRQVEFLSMKLASVNTRLDFNIDSLISKDMFQSNNSLAHPIFPLDSSAQAFYGHHTQQNPGIHNNILNGVVTHSTVNPLESASLCPNIGMQLPPLNGFNECASQFPIGFCEDDLHTIVQMGFGQTGHGKSPSDSQNFNGSNQVSHMKVEP

>BGI_novel_G001343 EpbHLH94

MSFCVPTLNVDNNIPSTPKTSLPSNSNPTVPDDLPMFDYEVAELTWQNGQIWMNGMGLPRLPAKPIIGTDNSGTNNRSYLLTSNSKHRGSGTLESIVNQATSFFNYNKSMSLSAGKDGGDKDKLVPWFESHHQASAVSNNNTMTMDALVPCSNRTDEQSTAVVDESMGGGIGLRGVGGSTYVRSSGGGNGAARLTKVARVPMVHPWDQSLSERDSHDVTSDTCDKELDLAYTSASMGSQENTSCVRLCTKMTVADDHDSICHSTQIEEDEEDEKKERIVKSSSSNKRRRAADVHNQSERKRRDRINQRMKTLQKLVPNSNKTDKASMLDEVIDYMKQLQAQVQMMNRMSISSMMLPMTLQQQQLQLMSMMAPMGMGLGIDMNTMARPNIPNIPPILHPSITGFTPMASWDASGAGNRLQGAMPDFLSSFLACQTQPMTMDAYSRMAAMYQQMQQVSASISKN

>Maker00014818 EpbHLH95

MSVFHVAVALSVVSFPVVASSCFSVFNFSMKIEVGLGGMLWNEDERDMVAAVLGARAFDYLVNHSVSNENLLMAVVTDENLQKKLSDLVERPNVSNFTWNYAIFWQISQSKSGEWVLGWGDGCCREPDDGEEDGLRGILSLRMEDEVQQRMRKRVLQKLHATFGGSEEDNYVYGLDRVTDTELFFLASMYFSFPRGHGGPGNCLASGKHLWLTDAQKSPTDYCVRSFLVKSAGIQTIVLIPTDMGVVELGSTKLVRENLELLQAIKSGFSIESTFPRTKSIPPPPEKRDELAIFPVLAVSDKAEIVPKIFGQDLNKTNPVRTTHFREKLAIRKMDDRPWLGVGGHSNGVHARNGVHASAWTANNGARQPSPAETYAPRSSGSNMPVLDLVNGVNNFQPQRQTPMQIDFSGAASRSVVRPNGGEPEQSGADASFREDLPGGTDERRPRKRGRKPANGREEPLNHVEAERQRREKLNQRFYALRAVVPNISKMDKASLLGDAIAYINELQAKLKAMESEKERPGGSTSGEASMESNPNQNQSPDVEIQAGHDEVVVRVSSPINSHPVSKLIKAFKDAKVNVIDSKLAAANDAVFHTFVIKPQGSEQLTKEKLIAAFSRESNSSQPLSLTVLDAEVLVFIQDLCIRVEVLVKFSTAGVT

>Maker00014967 EpbHLH96

MDDLIISPSSSSSLVCIPPRDNQPSLQQKLQFLLQSQPDWWVYAIFWQISNDDVNAGNLFLSWGEGHFCGTKDASPRLSSSTNIATTTSAAPHYPDVHPYRARFVNGIQSLLNDNCDINTTNICSDLHDTEWFYIMSLTRTFSVTSSASLHGKAFASGSPLWLKGDHDLQFCNCERAAEAYLHGIETLICIPTNHGVLEMGSSDLIPENWGLVQQAKALLGSDLIAKQPSNNLIQFFDDQNISFADIGIIAGVQEEDDDSHVPDNKKSRSDNNNKKKADDRNKAISGFKFGYADSEHSDSDCNNPSTEKRTPKKRGRKPGLGRETPLNHVEAERQRREKLNHRFYALRAVVPNVSRMDKASLLSDAVDYINELKSKIEELESKIQREPNNSNNNKKDVKTEMAETTDNQSTTTSTVEQTTSSTTGGASASGIEVEVRIIGGEAMVRVQSEKANHPGAKLMDALREMELGVHHASMWCVNELMLQDVVVKIPDDLRNDESLKSAILARFTS

>Maker00015087 EpbHLH97

MERLQGPINSCFFEERWEGKCFEQGLIENEDEEENLLISSLEDKMPFLEMLQIVESQSPQFFPFRDTNFQTLVNLKKPCEVEEKEKACIPGRTTEVQIHAPPELDSCVTHETAEMPSRSPAKSENCRRKRRRTRPTKSMEDVENQRMTHITVERNRRRQMNDHLGVLRSLMPSSYIQKGDQASIIGGAIDFVKELEQLLQSLEAQKRMRMNEDIGFSYGTTSCGKESNYGVEVKAENKSELAEIEVTVIQNHVNLKVQCKRRTGQLVKAIVGLEDLRLTILHLNITSSQAAVLYCFNLKIEEGCKLVSASEIAGAVEEMFRFINGS

>Maker00014719 EpbHLH98

MDGFSWDASRLVIDMPILWNNQQQRDIEEEAGYSMPDFDSSALYQVQETHNEPAQTDHHHYHLYTACDLVPDFPDTNNMAHHQQQQLLNGGVSHSDVDGGDGNVSMILSDNCGRNIWNFSNSGSAASSSENNNPPKAKKTRWDHKLPSSSNINFQQENEEPDPEAIAHMKDMIYWAAAFRPVNLGWEEVKKPRRKNVRISTDPQNVAARQRRERISERIRVLQKIVPGGSKMDTAATLDEAANYLKFLRSQVKALKSPGDKVHSMSCNPFSFINPSDYHI

>Maker00014856 EpbHLH99

MVMEPNLSPNNSFSFHHLEPDGQSFSNQFDFISLDGAAFVTDIFQPTGSLCSHAESDHTSSTVTVAADVDTRFNDNSDSTSTDLFESYRKRPAVSAFTLSFDNSSIIPKTNKSSNLTKTTSSLKRTRGDRKVEPRTKPISSSEAIRHIIAERKRRQDLTQKFIALAATIPGLKKTDKASVLCEAIKYVKQLQGRERELENGENRKGTKRKVESPDVCCCCRTHEAIPEMEARVWEDQVLIRIHCERRGDIMLLMVELLESIHLSVLCCSSLPFGNSAVSITIVAQMKEKYSMKLNDLMKYLRHRCMLKSKDSSSV

>Maker00014850 EpbHLH100

MDQVFWESWLSQPLIYLFNYVGPFSLIDLSSLVFKDMEAEVLKANNTHQDEEEFFLRGILQSPAELSSESEAVLSPLGVDGGAASNHSVFHSQQCASQRAPPTAYVLAFDQSTVIPAVPEPGFHSLNHGDDISISLKPKRTLENLNDEPRLNRGTKKARSSAGALDHIMAERRRRQELTERFIALSATIPGLKKIDKASILSEAISYVNQLQQRVRELEEEANNKMMKKSQSSAENNVVGGLHHSEMESDDCYTPNDVLPEVKVRISDNKQVLIRILCQKQKCTMLKILDELKNLHLNVFGNSVIPFGSYAVDITIIAQV

>Maker00014885 EpbHLH101

MSLFDGDQLVLQETDTTAAAGVHSNGSSYDSQKHVVPTATPATFVLSFDKPTTSHDCFDLNNKVFFGDVISATPVSMPSSSSTPKSKSRSRSSSESLHHIMAERKRRQIMREKFIALSATIPGLKKINKASVLEEAISYVKQLQNRVKELEEETAKTSIDSLILLKKSHLYVVEESTCREKKSGDCCNRLKGALPEIEARVFKNEVLIRIHCGRQEDVLVKILSQLKTHRLTAPKSCVLPFGNTLSITIVAQMGENYGMKAKDLVKSLKLALL

>Maker00005800 EpbHLH102

MENDLFLNAAALHFEPSSSPPPPSMLTWQSLSTALDIPVSSELLQQDCFYYPTWDRSPLDHHHHHHGLQFDSALSSMVSSPAASNSNLSGDNFVIRELVGKLGNIGSSGEISSYLNGNNSADTSCYSTPLSSPPKLSIPPLMNNHLMKDKLPMALNSSVAEFSADPGFAERAAKFSCFGSRSFNGRSSRQFGMNNNADITQRSSPLMENGKLPRVSSSPSLKALGSQTENKNSPLHSQEESTISEQNPNGESGVKPSHPDVNSRKRKVSSKGKPKETPSSANPPKFAEANEHSSAKRSKPTEGEGHENGQVKPEEESKGGNNNGGDEKENKSNNSKPPEPPKDYIHVRARRGQATDSHSLAERVRREKISERMKLLQDLVPGCNKVTGKALMLDEIINYVQSLQRQVEFLSMKLASVNTRLDVSIDNLISKDIFQSNNSLAHQAAIFPLDSSATAFYGHDPAINNTIPNGTVTHCSVESLDSALCQSLGFQLPSFNGFNEDASQYPMTFCEEDLHTIVQMGFGQTENGKTPIQSPSMNGSNQIPQMKVEL

>Maker00013655 EpbHLH103

MEASGRISEEWSSLSGFYTSEEDDFMTQLLGNNYSVPEELLYENSSMGFPCAFWPAHESTVVSLTGINNNTSYFPSSVANITNLFSFSQGSSSSNDSGISYYPDDPLANFDSISMGFSLGDSKIVPYSGQSNDHLRQQINENTDEESGLDPVKIVLADADKNLQADRECEIIVSESAEKGKSTRLEKSEKRSRSSLEVSKNKRNVRSKKDTKSASVNNNEEDRSFQRQSSSSCCSEDESNASQELIAGATSSPKDSASLKSNGKSVSNKGPATDPQSVYARRRRERINERLKILQNLVPNGTKVDISTMLEEAVQYVKFLQLQIKLLSSDELWMYAPIAYNGLNIGLDLNISPSKQP

>Maker00014088 EpbHLH104

MSDREKERENQSISDHSKKQMHPEKKSNSRGKEKYVTIDEIEIESHDLQIQPSAERERRKKIRTLLGSLHAMLPHIPLQADKGTVVDETLKYIKTLERTLKDLQKQKLERLQSVSDPCGTSRETFMAEKVSSSSSSSSGPSVPHYNPVGFRFQSWISPNVVVNICGRDAQFNVCCSKNRCLFSTICFVLEKHRIEILSAHISCDNDRRFYMIQAQENRDLDQIPGALSADQIFKQAAEEITLCVS

>Maker00013577 EpbHLH105

MSDQDRENQSDSDNTVESSEKSLREKRAVEDESHASCERERRKKMRLLLGDLQVLIPSVRPKADKATIVGEAVNYIRTLETTVEKMEKQKRERLKSASHSLNPTGNNTSGAFIACQASSSTATPAATSIPHNPVRFQTWILPHVVLNICGEQAQFSMCFPKKQGLFSVICYVLQKHHIEVLSAHISSDNNQSFYMIQANVSIYVFLQANRHSEAFSAEETFKKAAGDIELWLT

>Maker00014043 EpbHLH106

MPRCLNSSDMTVVETQRDGIKWQHQHQFHHHNYHSYFSSNGFDAAFSSSLQLQNYPASMMINVTGVGGSGIADVPSNVESVKPDPGLENGRLESGKLEMPGLGFVSSGFDVSSAISRTSSCPPSAAVEKMRWTSGRESFKKRKFDKVQNPKVVAENEKKKKNKTSNEEGESKVTTNNNDKNRETSAESSKENSKASEVQNQKPDYIHVRARRGQATDSHSLAERVRREKISERMKYLQDLVPGCNKITGKAGMLDEIINYVQSLQRQVEFLSMKLAAVNPRIDFNIDDLFTKEVFPACAPTSFPAIDMSMTPNPAYLQFDPAHAALDMDINPPDFGIRRTISGPISMPPETLLDSSCFPQTLPFSTCEADLQNLYNVAYDQARSTTSFPSQPFTGLIEASNLKMEM

>Maker00013643 EpbHLH107

MGLPSIPNNVYKQLRFRLSFAEMMRILALAPLSSLINHTAGDMNISTQNLSERLRPLVGLNGWDYCVYWKLSQDQRFLEWMGCSCAGAESSQNGEELLFPFSSAAPCRDNVFPHPRTKSCAFLYQLPTSIILDSGYAFNFSDFTLLTIVIFKSCISPIYIMIRIHAETLLSNQPSWVNYSNSLDPNTLEETIGTQVLIPVPGGLVELFVTKQVPEDHQVIDFITAHCNMDMSFDIDVNSISHMQSNMNPLLGDENDGNTIASETSSLPPHEQCNSPLNFMQQFNHSNPQNRMKSDAFFEDSQGPFLLDKTQQFGEPLEKKEQQGDDKDLIKHVVGRSDSMSDCSDHNEDEDDGKYRRRNGKGNQSKNLLAERRRRKKLNDSLYNLRSLVPRISKLDRASILGDAIEFVKDLQKQVKELQDELEEHSEMDERQPPDNGVGIEVPKADQTKIQNGFRVGKSGNGYISKRKQEFDAPNDKQTQQMEPQVEVAVIEGNEMYVKVFSEHRAGGFVRLMEAFHALGLEVIHATVTSHKGLVSNVFKVEKKENEMVVKAEEVRESLMELMRNPSRGWNHEMSSSAASENGVARDFHQHHHLHSSHMPPYTPHHIHHIHT

>Maker00002890 EpbHLH108

MFPSQRGINEMVIEFSSRPHHHQQHQISEDLILDGHPHASLDIINFSDKQLQGSTNQRKKLLYGSSDDKINQYRIPDKDCKKKKEKLVHREIERQRRQEMATLYASLRSLLPLEFVKGKRSISDHMNEAKNYIKHMETTIKELSAKRDKLKKLSYDSSNPETQECEEYTSSGCFSIREINNNGGVGIEVSSCFGDERLPLSKLLELVHQEGLEVVNCVSTQVNGRLIHSVQCEVNSSDRTDVPTLRRKVVNLVPSFRCSD

>Maker00003075 EpbHLH109

MSRVKNHHIPLPSSDNNYDDEARVCDNEKKIIRREIERKRRLQMTSLCESLRSLLPFELIKGKRSVSDHIGEAASYIKDLKEKTQELQIKRDKLKQEANSSLLGAEINESSSHSNIVRCVSISPFPAGLEISVDCGLEESSFPLSLLLEILMQEGFNVLNCVSTQVNGRLYHSIKSEVDNSESLDVAGLEQKLNDAILWSRYNCLD

>Maker00003000 EpbHLH110

MMNPLHPVGDEDLFFQISFLNPPSLSIQTIPEDQILHDLGDNDHERCPPNYESEVIPKGKGKRPKQQEFPAKIVKDADKKWVHRQTERQRRQEMAKLCASLRSVLPVEYIRGKRSASDHMEGAVNYIKRLKNKVKQLKAKRDELMKNNGNGISVIVHPYTSGAEILCSYSFRKYGFTQSTLLSMLIKEGLNMVSCTSTKTDERSNHAIRLEVPHIPMLACLIYRLNCYL

>Maker00003031 EpbHLH111

MERFRTDSVMPLPSLVSQVPPSLASAQPVVAMVETGKTSGKRRRQRGLERSETLVMERDRRLKMTHMFAELQATVPGLLSKATREVIVNETINYIKDLEKKKKRLEDLKGLVMEPDVGRKLMLPCTSNRSSSITLSVSGNVAFFGIQSKAQFGLITVIFKVFYKHNAEILAANVSVNDGELTLAITAVLVHDGDDNEQVSAEKIKTEILSL

>Maker00009383 EpbHLH112

MERFRTDSVMPLPSLVSQVPPSLASAQPVVAMVETGKTSGKRRRQRGLERSETLVMERDRRLKMTHMFAELQATVPGLLSKATREVIVNETINYIKDLEKKKKRLEDLKGLVMEPDVGRKLMLPCTSNRSSSITLSVSGNVAFFGIQSKAQFGLITVIFKVFYKHNAEILAANVSVNDGELTLAITAVLVHDGDDNEQVSAEKIKTEILSL

>Maker00009314 EpbHLH113

MSHIAVERNRRRQMNEHLKVLRSLTPCFYIKRGDQASIIGGVIEFIKELHQVLEVLESKKRRKSVSPSPRAPREAFHLDHHNSPLKEEVGAASCNSSVADVEVKISGSNVILRVISHRIPDQVSKIITVLESLSFEVLHLNISSMEDTVLYQFVVKIGLECQLSLEELAIGVQQTFCSEAVN

>Maker00020633 EpbHLH114

MTDYRSSPTMNLWTDDNSSVMDAFINSDISVPWPPPPQPQSSASTTNPGPDPTKTLSQSQSSVALFNQETLQQRLQALIEGARESWTYAIFWQSSYDYSGASLLGWGDGYYKGEEDKGKGKAKTLSLAAEQEHRKKVLRELNSLISGSSPTNDYAVDEEVTDTEWFFLVSMTQSFVNGDGLPGQSFYNSTPIWVTGGERLSVSSCERARQGQVFGLQTLVCIPSANGVVELGSTELIFQNPDLMNKVRVWFNFNNPEIGSWPVMGFNADQGENDPSSLWLNDPSSGIEIRDSANTTVPSAVPSSNNSHNIQIIAKPMHFETPLSSTLTEAPSGVHVPNAQYSQQQRQLPQQSQSQAFFSKELNFSEYGFDGNGVKNGNLHSLKPESGEILNFGESKRCSYGGNGNFVTGQSPFVAEDNKKRRSPTSRGSNDEGMLSFTSGGILPASNIKSGGGGGGGDSDHSDLEASVVKEADSSRVVELEKRPRKRGRKPANGREEPLNHVEAERQRREKLNQRFYALRAVVPNVSKMDKASLLGDAIAYINELKSKLENLEISKDDLQKELDSVKKELELATSKDSRPPPSDKEHNTSNNNESGKLIDLDIDVKIIGWDAMIRIQCSKRNHPAARLMAALQELDLEVHHASVSVVNDLMIQQATVKMGSRFYTQEQLRQALSSKVGDVR

>Maker00020701 EpbHLH115

MALLESLSSNDLFNFIIHDTISATPFPHHVHEPFSEANFLLENRVTLMEHDNNASASSSSRKRHSDELQVPIVQGRKKRRRKPKVCKNKEEAETQRMTHIAVERNRRKLMNEHLAVLRSLMPESYVQRGDQASIVGGAIEFVKELEHLLQSLEAKKLQFLQQEVTQSNEESCSIFKLTSPPFAQFFVYPQYTWSQAPNKYPSKAKAAIADIEVTLIETHANLRILSRSSPRQLSKLVSGFQTLYLSVLHLNVTTMTPLVLYSISAKVEEGCQLGSAEEIAAAVHHLLRRMEEEASLCG

>Maker00020299 EpbHLH116

MDPGAMMNETSYGNGGANTAPYNLADIWQFPMNVGAGLGDSGGGLGLRRPQFGHNLGQFVDFPGANRDVPGNDQISSEQRGNHGVNNRKRRDSEEESTKGVSTSNGGNGVNDGGEGKRLKASGNRNESRDTKTETEPSSGKPADQSNQPAPEPPKQDYIHVRARRGQATDSHSLAERARREKISERMKILQDLVPGCNKVIGKALVLDEIINYIQSLQRQVEFLSMKLEAVNSRLNPSIEVFPPKDFGQQSFETAGMAFGSQATREYSRGSSPEWLHMQVGGGFDRTS

>Maker00020533 EpbHLH117

MSSTRRSSSSSRQSGAAASTDITEAQINDLVSKLRQLIPELRAPRRSGKVSASKVLQETCNYIKSLHREVDDLSERLSELLASTDSDSAQAAIIRSLLM

>Maker00020873 EpbHLH118

MGYTAKKKKSSSFSSMVHRLYSFSFFGGHKKTSKAVAAPSVAEATMVAAMKHFSSPHKVRNTSKAVAAPATMVAAMKHFSSPHKIYEQKSKAVAAPGGVGEATMVAAMKHFCTPHKRRMRAGKGNQEDDEYEEEEFGSKKEGPSSAPNNNNTNKDAKAIDKASAIRSKHSVTEQRRRSKINERFQILRDLIPHSDQKRDTASFLLEVIEYVQYLQEKVQKYEGSYQGWSQEPTKLMPWRNSHWRVQSFGQPQAVKNGSGPVSPFPGKFDEGNITVSPTVISGSQNMIDPEQSRDIVSKSAERHAELECKGIPLPMPMHANLSVPVRSDGALAHPLQGPVSDGQPTECSANSEPQSQQEELTIEGGTINISSVYSEGWVVAKSYSFPLCSLRIGKALGPVAAVYELAILDFLT

>Maker00020658 EpbHLH119

MYEEMTGFSAGTASLLSEDCFSQMATSVRTQTHNPSLPENVGLSMENLAFSSQDTIANISASALVEANPYGDNIVNTDHMVEEVNHHDSNQLSLSFDLSKWDTTMNHHHQNHNQHQDQPLLYPTPDPRNCNLFYSLPSSASASSFLNSSLSFGNPAIQKAFELHSPSGHGGLIGGSHPTLGAENTHVSSVLLQDPFLHLNLPARPPAIKEWFQSLPSRNDFLLFGGDDDKEGSGVASYLDADREGEFGDDNNDDVLKFSQDLAPIGRKGGGRGGKRVRQVTTEKKRRVDFTSKFDALRELIPIPTKNDRASLLCGAIEYIQELRRSVDDLTLLVNKKRCGKERIKRHKVDEEDDEEEASGDLESCNLKPPSEPDHHLHAFNSCIIRSSWLQKKSEDTEVDVRIIDDEVTIKLVQRKNTNCLLYASKALDELELDLQHVAGGHIGDFCSFLFNSKVYICRFFCVRECHSQQTYGSHGSSSLKECH

>Maker00020407 EpbHLH120

MNRMRKEEDQPQCSSSHAINNIQSYQEQLLLQQMMQQQQHQNPDVFGGNSNNNTSNNERGLMFPGVSLVFQPPPPPPPPWSLPPVHSFNPVQETDQFLVSPPSSSLYAGPFNRRVPSLQFTYEGPSNSDHHHLRIISDTLGVGPVGQPSSAPFGLQAELGKMTAQEIMDAKALAASKSHSEAERRRRERINNHLAKLRSLLPSTTKTDKASLLAEVIQHVKELKRQTSIIAETSAVPTEADELTVDDASDEDGRFVIKASLCCEDRSDLLPDLIKTLKALRLRTVKAEITSLGGRVKNVLFVTGEEDELSGEEQRHQYCISSIQEALKAVMEKNGSGDENGSVKRQRTNNNINILEPNRSL

>Maker00020560 EpbHLH121

MEQNSSSNIQEFMAFLNDSFSDQPLNLPPHSLHSSISPNSVHEDAETTFPVSETLLASSPSFSAPSSAEKSKSMVISKKKRNSNRYEVLEEKPKEVIHVRAKRGQATDSHSLAERVRREKINEKLRYLQELVPGCYKAMGMAVMLDVIIDYVQSLQHQIEFLSMKLTAASMHYDFNTCENNVAETLQRNTNTTWEVQEIEKMGEEGYGGYSHFNPTWPSLR

>Maker00020823 EpbHLH122

MDDADGFLDQHCHMTMMCFDQEDEDFLRDIMQQPSGHHVSSQTQTENNVESPRCNIDVKPVLISKQDRPTTSDGCRLKRSRAPCDDVETRKEKRSCSERLDHLMAERRRRQQISERFIALSAAIPGLKKLDKATILDESTKYVKELQERIRELERESSLISMKKCELQGNESNEAVHEIEVKVAENQVCIGILCEKQKGIELKIMRLLESFHLCVTSSSVLPFGNSTLGITIIAQMGDEYKTTMKELVKNLRQVVLHEVDLC

>Maker00020880 EpbHLH123

MDEPWQTSFPQLLDLDDVEDFHDIMMSFDVEDENIVQKPGRYYFSSQGDQTEDHNGGSGHHGSKVVNHVMSPRAYILSFEAEQGEPNTLKRCSAKRVRERNNVEPRKRRRRSEPLDHIMVERRRRQQIAERFIALSATIPGLKKVDKVTILDEATKYVKQLEERVRELEKESSVISMKRIEVCEHGDLCEDSEGVPKIEVRVSENQVLLGIFCEQHKGIQLKIFTLLHRFHLHVTSCCVLPFGNSALDITIIAQMDEEHRMRMRMNDVVKNLRQLLLKLGEMGDEDRMRLDDDDVENLARTDSIADE

>Maker00012061 EpbHLH124

MNKKKVEFLFEMDQWKPEDLSKSVSVVADLAPSSLQPQSQLITVTPNRLQNSHTESRQRPEAEAQDPVVAARKLQKADREKLRRDRLNEHFLELGNALDPDRPKNDKATILTDTIQVLKDLTAEVSRLKADHAALSEEAHELMQEKNELREEKTSLKSDIENLNAQYQQRVRVTLPWTAIDHSVVMAPPYSYPVPIPIPSGPISLHPSLQPFPFFGNQNPSAIPNPCSTYVPFSAPTNPPIEQPSGQFASTSHISSKQDSRCKSPDHERTSNADRCGESNDVATELELKMPGSSTRQESSSGTRKGKHSSRKERNITDGSASSRHSSSQGLQDSSNSVGDIPKADK

>Maker00011748 EpbHLH125

MGDVDMPRTSGGEAISMDMISMMMQLEKFPLQFSDPFFNTPTGYPQTEGGNIATTSFPAFPNPNVSPDFVSSPVQQPFAPPPLPQPPSDDDLPATSLGNRYNPSISSTFPFASDKMNSMAAMREVTFRMAVMQPIQIDPESVKPPKRRNVKISKDPQSVAARHRRERISERIRILQRLVPGGTKMDTASMLDEAIHYVKFLKKQVQTLERAGVSRPIGDAFTGISRIPTFSNGGFNANA

>Maker00011873 EpbHLH126

MGDMYNNSSSNLDDININHYNFPASFTSALSSSSPQDEFSLFLRQILLPSYSSSPSSPLFLTHTGKNAHTSMAGFCSSRARLDNDQPPDRSSVFQNAISVVDASIGVSTSNDVFLSSLGTYASANMKESSTMNMPSSSFGVSENEFDEYDCESEEGIEALVEEQPAKSVPPRSSSKRSRAAEVHNLSEKRRRSRINEKMKALQNLIPNSNKTDKASMLDEAIEYLKLLQLQVQMLSMRNGLSLHPMCFTEGLQAQQFCRMRSEIEENPLQYVSKSSLSDKPNARDQPLVPSPSYIIYSEGSFGLEPRGPFQPKTTSEMRSDEILQHQQLNASHSDANPLGGSQAVHTHDMQAAEKKDEGFETGIAGKDELAMILRNSNDNVTHTSRFRRLRAVKRMTTWRQRNQVSEL

>Maker00012210 EpbHLH127

MMEISSKYFTELGMEDPSPFEHYPVEECLGYEVGELQGLSGCGDSNSCFNSKRSRYDWFPVESPESVAPPRAAKQLKSFNNGWVSCTTTIADPIVPSSNTSTSNSPSQIISFENSNSSSDVASHPFYSLESGVVKPKTESIVFTDNLDFSAPFSVSQPSFDPKLDSFFLPQEKKAPATIARNSIQAQDHVMAERKRREKLSQRFIALSAVLPGLKKMDKSSVLGDAIDYVKQLQERVKTLEDQVAKKTVESAVFVNRSILFADDDGSLSDENSSSRSDQPLPEIEARVSSKDILIRIHCDKHNGCAPTILSQLQQLHLTIQSSSFLPFGDTALDITIVAQMNKEECLTAKDIVRSLRQGLRHLI

>Maker00012137 EpbHLH128

MVPQSLKKQLALAVRSIQWSYAVYWSASPNQPGVFEWSEGYYNGDIKTRKTSQAVELNSEQIGLQRSEQLRDLYESLATAESSPQAKRPSAALSPEDLTDTEWYYLVCMSFVFNIGQGLPGRTLAKGQPIWLCNAHFADSRIFSRSLLAKYEQSNQGTAIFKSFVNFQTVVCFPFLEGVLELGTTDLVSEDPSLIQRIRTSYLDILYAKVCKSSGVALNTRDGEDVSCAVFDHNAFDVKLTLESEYEVANATSPNNSSNEFQANKQADERLIIERINGVASHVHSWQALEDELSNCAHNSLNTSDCISQTFAGSEKNASVPKGDHCGQDLQECKNTKMTSVDIRRDDWHYQRVLSSLLKISDQLIMGAHFQNSHCESSFISWKQEGSMGCQWPRGETKQKILKKVLFEVPRMHIDWLLESQEENDHKEGMRPEADEIGLNHVQSERRRRAKLNERFSTLKSMVPFITKDDKVSILDEAIEYLKKLEKKVRELEARREIKDSETRTKRALQDVVERTCDNYCNNRTDNGKKPVMNKRKACDIDETDTETETDSVRLKDYSNNVTICMNDNEILIEIRCPWREGILLEIMEAVSNLHLDFHSVHSSEADGILHLNMKSKIRGAAFTSTKMIKQALLKAAWKY

>Maker00012273 EpbHLH129

MSVAEGRPPVTFLATIMVTGEGLRLSDEKRNGLRFLHYLNNFPPLKQLHSLPQAFCVWITISPSHLQREEKARLDRKVMELTQLGFLEELLAPRRETWSTFSSGLSEIFPNSAYPFVDGLTVTEFNSSYTRNEDSALFPAQESNPSMEDEEFGFLDSENQSRQEINSACKIGEQATEAPVFNTGLRGEKKPKSKKLEGQPSKNLMAERRRRKRLNDRLSMLRSIIPKISKMDRISILGDTIDYMKELLERIGKLQEERAQEDINQINLQGNNSKEMKPNEVMARNSPKFEVDKRDQVSRVSICCAARTGLLLSTVNTLEALGLEIQHCVISSFNDFSMQASCSEVYMILIRTGISSEEIKQALFRNAGYVGRCL

>Maker00004708 EpbHLH130

MSCSSSSSRSLVSLGPESSSTLQQRLQFILHSRTEWWAYSIFWQACKDDGNGGNKRQVLNWGDGHFRGSRDLKSKDSSSSNELRCPSRARKTRLIPNTMEDDGHVTDLEWFYTVSLTRSVAAGDDVVGRAFSSGTPVWLAGELQFHECERVREGSAHGIKTLVFIPTTIGVLELASSELITQEWSLIQTAKSVFDFDNGFITSAPPPPPLLSESHHHHFNNESFMDSETLLLSASADTTTKPRPRRKARNDAMKEQELSPLNHVEAERQRRERLNQRFYALRSAVPNVSKMDKASLLSDAVTYINQLKAKIDELEASFKMHPQKQELQEGATAMRHWSSTSEPAVGVEVNIVGTEAMIRVQCLDVNHPAARLMDALRQLDFHVHHASISRVKDLMLQDVVVRVPAGKFMTAHTIKNEILQRLQN

>Maker00004238 EpbHLH131

MVPENMKKQLALAVRSIQWSYAIFWSASVSLPGVLKWGEGYYNGDIKTRKTSQAVQLSSDQIGLQRSEQLKELFESLKTTEATPQIKRPSAALSPEDLTDTEWYYLVCMSFIFNIGQGLPGRTLAKGQPIWVYNAPFVDTTVFSRTLLAKTVVCFPFLEGVVELGTSDLISEDLSLIQRIKTSFLDILNASKPGSTFKTRNNTDVASEAFGHEAYDAKLTEMGCVITNKTSANSSNVIEANQLVDVTHLIEMINGGASQVQSWQVMDDALSNDVNNSNSSDCSSQTFADSEKIASVPKDQNLTNHSAQGLQECNKTKMTLVDHRRDDWPYQKILSVLLKDSDQLILGAYFQSSFKESSFISWKKGGSMSCHRLRGGTPQKLLKKVLFEVPRMHMDGLLECQEDNDCKEQMRPEVDEIGTNHVVSERRRRAKLNERFLTLRSMVPSVSKDDKVSILDDAIEYLRKLEKKVRELEACRELMDLETKAKKTPQDMVERTSDNYFNRNDNGMKPMINKRKACDIEETWQENDSIALNDSSTNNITVNLSNTEVLIEMKCPWREGKVMKIIEALGSLPLDLQSIQSSEAEGILHLIIKSQLRGPTSLSEKKIEQVLQNAAWKC

>Maker00004271 EpbHLH132

MNSIDDDQFISHDIASVLQGQYLQPSLSCESLSSYKSSTGETIFDRPSKLYLKSNNSWNSTISELASSSSSQLLSFDNSNKSSAVNSTNFFYGFEYSTLKPNNHQFSDMKYSQNHNFEPRSTEGINDRPSPHVRDHIMAERKRRQKLSEGFIALSDLVPGLKNMDKVSVLGDAIKYVKELKQRLELLEEKKKKRRVESVVIVKKSQQCREDDWSSTSERSNGVSEEEALPEVETRVFEKDVLVRIHCQKQKGLLARILAEVKVFIFVLSTAASCRSETSLLTSPLLLRYKNILYGGKLQLDDEGSREEPTSGYFKFLSTCDISEV

>Maker00004561 EpbHLH133

MASQVDSVFMLQDKAAFPSNGYYLQSQRNIKERDRPEGRNPRVQDHLLAERMRRGKITQHFIALSALIPGLKRVDKVSVLEEAIKYVKQLQEKVKILEERQITKNKGVKSVVFVKKSQVMHSDEHVSDTSFDFSTDIENIHDPQKRYGAVTEADVEARVSDKNMLIRIQCEKQEGTVVKTIREIEKLHLSVVNATVMPFGRSILDITIVAEMEDEFNLSVKELARNLREALLQSM

>Maker00004512 EpbHLH134

MMEISSAVYLPEHGMEDMTLFEQYPMDIFEGLDFQSLVSESYSSYECPYSDRKQNSQSIAQPAPRPSKQVKSNIKAEASNSDSRLISFEHSNTHRFHNLDSIAKLKTLMNERVLDFGSSSDQGAYYEDIQFGQCEQQTNMAGTVTRNPLQAQDHVMAERRRREKLNQKFIALSSIIPGLKKMDKATVLGDAIKYVKLLQEREKILEEQAAMKRKESALLMRRSILFAGDDGSSTSNEISDQPLAEIEARVSGKDILIRVHCEKHKGCEAAILRQLEKLHLTVQSSSILPFGETTLCLTIVAQMDEDHCMTAKDMIGNLRKAMALDSA

>Maker00004499 EpbHLH135

MGDLYGYGKSNIIPSSSSISPPDEFSLFLHQILLPPSSAPPPSSLVTHMPENAPSTVGHEDASLSHPSLLLQDVISAVDSTSKNHAFLSSYIKGGSAANVSSSSVGVSGNETDEYDCESEGGTVEAMVAKSGPAGRSSKRSRAAEVHNLSEKRRRSRINEKLKALQNLIPNSNKTDKASMLDEAIEYLKQLQIQVQMLSMRNGLNLLPMCFAEGLQPLHLSQMRVECGEEEDRSCPLNFTATLPLDHASNLSNKRIVPSLSNLTNSETSFGLDSQIAGHLRPFESSSGQICREDILRHHQLNADNSNTNPLGDSQGVKGFESGMVATVPLSFDEQNDRSDGGMKPCSQEEISNELF

>Maker00004286 EpbHLH136

MDVDDRSMDMMAMMMQMEKFPEFFEPFYPEPQFSTSTTTTTTSLPILTNNNLINNPSPSFVTNTINPIPQLMTPNLQPKDVGFRNTDNPNASSSEKKNSMAAMREMIFRIAVMQPIHIDPESIKPPKRRNVKISKDPQSVAARHRRERISERIRILQRLVPGGTKMDTASMLDEAIHYVKFLKKQVQTLEQAGARTMGYGVPTTLNMNNYSTFMKGYHQSSQMVGSSMQMLS

>Maker00023061 EpbHLH137

MNLWTDDNSSVMDAFIGSDLSSLWPPPQSSASDITIAGPDPTKALPQPSVCLFNQETLQHRLQSLIEGARESWTYAIFWQSSCDYSGASVLGWGDGYYKGEEDKGKGKAKTMSSSVAEQEHRKKVLRELNSLISGSSASPDDAVDEEVTDTEWFFLVSMTQSFYNGGGLPGQAFLSSSPVWVAGSERLAASTCERARQGQVFGLQTLVCIPSASGVVELGSTELIFQNSDLTNKVRDLFNFNSPEGSSMWPSNASTATDQGENDPSSLWLNDPSGIEIRDSVTTVVPISVPSSTQSHNNNQNTAKQIQFDTRGSTTLTETPSSIHIPNAHQNHQQQRQQQQRQQHHSLNRGLFSRELIFSDYALDGNGSKNGNSHSLKPESGETLNFEDNKRNAYSGNGNLLSGQSQFNAEDKRKKSPTSRGSNDEGMLSFTSGVILPASNMKTSGGGDSDHSDLEASVVKEVDSSRVVEPEKKPRKRGRKPANGREEPLNHVEAERQRREKLNQRFYALRAVVPNVSKMDKASLLGDAISYITELKSKLQKIESDKNGLQSQLDSVKKQLELANEDSGPPPLEKEPKPSNHQTNKLTDLDIDVKIIGWDAMIRIQCSRKNHPAARLMAALKELDLEVNHASVSVVNDLMIQQATVRMGSQFYTQEQLRLALSSKVGDVR

>Maker00022433 EpbHLH138

MELTQLSFLEELLAPRRGDPWTTMSSGLNDLFPTTSWSFDSFDDNPSALSSFNLPFSALPPPLIDQRFEYSCPFLDSFTGSNVVSAPPLQAQEDEELTFLGFENQSSEEANNNNNTSSYCKIEEQVMEAPVFNMGLCCGEKKPKSKKLEGQPSKNLMAERRRRKRLNDRLSMLRSIVPKISKMDRTSILGDTIDYMKELLEKIGKLQDEMEKEGGNNKINLIGISKEVKATEAMVRNSPKFEVERRDEDTRINICCATKPGLLLSTVNTLEALGLEIQQCVISGFNDFSLQASCSEVAEQGACTSSEEIKEALFRNAGYGGRCL

>Maker00022925 EpbHLH139

MQTRNYYNSEVYKLRNSFSRGTIPETYRNSFLKPKSKADTKLLAAKKHSEAEKRRRMRINGQYATLRTILPNLVKMDKASVLAETIRQVTELKKNVSELEVASASSSGDISVFPSAADKLNLERCNDEAGLVKATLSCDDRPGLMLAITRALKSVKAKVVKAEMVTVGGRTRNLLWVQGLENGNEGIGVLKRILKVVMRKPTFKVHRLTQ

>Maker00023085 EpbHLH140

MDPAAINIMNDGSFANGNTDTAPYNLVEIWQLPMNGDTGLGESGGGLELSRPQFGEFTSGPNQDGSGNDLNCSEQRANGFNRKRRDSDDELVKGVSATSNGGSGNSNDVNDGDGKRLKASSNRNESHDAKTKTETSSEKPVEQSTQAASEPPNPAYIHVRARRGQATDNHSLAERARRKKISERMKILQELVPGSNKVIGKASVLDEIINYIQSLQRQVEFLSMKLESVSSRVNSGIEIFPPRDFGQQTFDAAGMAIGSQAMQEFSRGSSPEWLHMQVGGGFERAI

>Maker00023071 EpbHLH141

MSSRRSHRSRQSGVDGTEISDAQINDLISKLQRLIPELQSTRSHHHQVSAAKVLQETCSYIKKLHKEVDDLSDRLSELLASTDSHSAQAAILRSLLTS

>Maker00002635 EpbHLH142

MKREEDHGQCSSQSINNFHSYQEQLLLQQMQQQNSMFPEVSPILQPWAIPPVHSFNQAAHFSNPVQVDHHDPYLLPPPPPPPGPYAGLFNRRVPSLQFAYDGSTSDHLRILSEALGPMVQAGSAPFGLQAELGKMTAQEIMDAKALAASKSHSEAERRRRERINNHLAKLRSLLPSTTKTDKASLLAEVIQHVKELKRQTSLIAETSPVPTEVDELTVDAADEDGKFVIKASFCCEDRFMCRRESDERSGGVGICKRVRQDGK

>Maker00002522 EpbHLH143

MVTIVGFFYYIVCILQKAVGFKRKRNNNRDGEKQKGVIQVRAKRGQASDSHSLAERVRREKINVKLRCLQELVPGC

>Maker00007729 WDR

MGASSDPNQDGLDEQQKRSEIYTYEAPWHIYAMNWSVRRDKKYRLAIASLLEQYPNRVEIVQLDDSNGEIRSDPNLSFEHPYPPTKAIFIPDKECQRPDLLATSSDFLRIWRISDPEEHPTPRVELKSFLNGNKNSEYCGPLTSFDWNEVEPKRIGTSSIDTTCTIWDIEKETVDTQLIAHDKEVYDIAWGGVGVFASVSADGSVRVFDLRDKEHSTIIYESSEPDTPLVRLGWNKQDPRYMATIIMDSAKVVVLDIRFPTLPVVELQRHQASVNAIAWAPHSSCHICTAGDDSQALIWDLSSMGQPVEGGLDPILAYTAGAEIEQLQWSSSQPDWVAIAFSSKLQILRERWLKA

>Maker00001466 WDR

MDNSTQESRLRSENSVTYDSPYPLYAMAFSSTPSLASPHQRIAVGSFVEDYNNRVDILSFNADSPSIRHQPLISFDHPYPPTKLMFHPHAHSSLRKSSSDLLATSGDYLRLWEVRENSVDAISLLNNSKTSEFCAPLTSFDWNDIEPKRIGTSSIDTTCTIWDIEKGVVETQLIAHDKEVYDIAWGEARVFASVSADGSVRIFDLRDKEHSTIIYESPQPDTPLLRLAWNKQDLRYMATILMDSNKIVILDIRSPTMPVAELERHRASVNAIAWAPQSRRHICSAGDDSQALIWELPTVAGPNGIDPMSMYSAGSEINQLQWSAAQPDWIAIAFANKMQLLKV

**Supplementary File 3.** The sequences of transporter genes involved in the network.

>BGI_novel_G000295 (phosphate transporter_2)

MADDQNVLNALDVAKTQWYHFTAIVIAGMGFFTDAYDLFSIPNVTKLLGRIYYTEQGSAKPGSLPPKASMAVNGVALCGTLVGQLFFGWLGDKMGRKKVYGMTLALMVVSSLASGLSFGHTAKGVITTLCFFRFWLGVGIGGDYPLSATIMSEYANKKTRGAFIAAVFAMQGFGILAGGVVSVIVATAFDHAYKSPPYKVNPAASLPPQADCVWRIILMFGAVPAALTFYSRAKMPETARYTALVARNEKQAALDMARVLQVELEAEQPKIEEKPSEIRERNSFGLFSKEFARRHGLHLVGTCVTWFLLDIAYYSSNLFQKDIYTAVGWLPAAAEMSAIHEVYMVARAQVLIALCGTVPGYWFTVAFIDILGRFFIQLMGFFFMTVFMFALAIPYDHWTKKENRIGFLVMYALTFFFANFGPNATTFVVPAEIFPARLRSTCHGLSSASGKAGAIVGAFGFLYAAQSKDPAKTDEGYPPGIGMKNSLIMLGAINFVGMLFTFLVPESKGKSLEELSRENENGDGDQNVDHAADSSSSRTVPL

>Maker00005004 (H+-transporting ATPase)

MAEELDKPLLDPENFNREGIDLERIPLEEVFEQLKTSPRGLSSDDAEARLHIFGPNKLEEKRENKILKFLSFMWNPLSWVMEAAAIMAIALANGGGEGPDWQDFVGIICLLFINSTISFIEENNAGNAAAALMARLAPKTKSALTGESLPVTKRTGDEVFSGSTCKHGEIEAVVIATGVHTFFGKAAHLVDSTEVVGHFQKILNMCQEKDEIAGKVHAIIDKFAERGLRSLAVALQEVPEKSKESPGGPWTFCGLLPLFDPPRHDSAETIRRALNLGVNVKMITGDQLAIAKETGRRLGMGTNMYPSSSLLGRNKDESEALPVDELIEKADGFAGVFPEHKYEIVKILQEKKHVVGMTGDGVNDAPALKKADIGIAVSDSTDAARSAADIVLTEPGLSVIVSAVLTSRAIFQRMKNYTIYAVSITIRIVLGFTLLALIWEYDFPPFMVLIIAILNDGTIMTISKDRVKPSPTPDSWKLPEIFATGIVIGTYLALVTVLFYWAVVETTFFESLFHVSSLSSDSEKVSSAVYLQVSIISQALIFVTRSRGWSFLERPGVLLMCAFVIAQVVATLIAVYAHISFADITGIGWGWAGIIWLYSLIFYIPLDIIKFTVRYMLSGEAWNLLFDRKTAFTSKKDYGKEDRAAQWVLSQRSLQGLMTTELSEAKNGMGRRSSLIAEQARRRAEIARLGELHTLRGHVESVVRLKNLNRSVLQSAHTV

>Maker00022863 (TRANSPARENT TESTA protein)

MPIREASNSQLGEAKVPLLEPHLSVLAPKNEQGDADETLSRRVWVESKKLWHIVGPAIFSRLASYSMLVITQAFAGHLGDLELAAISIANNVVVGFDFGLMLGMASALETLCGQAYGAKKYYMLGVYMQRSWIVLFICSVLLLPIYLFATPVLKLLGQTEELSELSGTVSMWMIPLHFSFAFQFPLQRFLQSQLKTGMIAWVALMALIVHIIVSWLFVYQLQFGVIGTAATINISWWVLVFGLFAYTACGGCPDTWTGFSIEALSGLWEFVKLSAASGVMLCLENWYYRVLILMTGNLQNAEIAVDALSICMTINGWELMIPLAFFAATGVRVANELGAGNGKGAKFATMVSVGTSMVIGIVFWTLIMVFHNQLGYIFSSSKAVLDEVNNLSLLLAFTILLNSVQPVLSGVAVGSGWQSYVAYINLGCYYLIGVPLGFLMGWFFHLGVMGIWAGMIFGGTATQTLILCLITTRCDWDKEAEKAYLHLKKWENPNHQVA

>BGI_novel_G000936 (phosphate transporter_1)

MGKEQVEVLNALDVAKTQWYHFTAIIIAGMGFFTDAYDLFAISLVTKLLGRIYYQQGNSLKPGTLPPNVSAAVNGVAFCGTLSGQLFFGWLGDKLGRKTVYGMTLLMMIVCALGSGLSFGHTSKSVIATLCFFRFWLGFGIGGDYPLSATIMSEYSNKKTRGAFIAAVFAMQGFGILAGGLFAMIISAAFKSRFDAPPYQVDPTGSTVPEADLVWRIILMAGSVPAALTYYWRMKMPETARYTALVAQDAQRAAADMSKVLQVEIQADPQKIEKKTHQTKGQSFRLFSKKFLSRHGLHLLGTSSTWFLLDIAYYSQNLFQKDVFSAIGWIPPANHECSRRALLHCQGSNAHCSVQYDSRLLVHRGFH

>Maker00021223 (H+-transporting ATPase)

MAAGKASITLEEIKNEAVDLERIPIDEVFEQLKCTKEGLSSQEGTNRLQIFGPNKLEEKKESKFLKFLGFMWNPLSWVMEAAAVMAIALANGDGRPPDWQDFVGIICLLVINSTISFIEENNAGNAAAALMAGLAPKTKARAGVREVHFLPFNPVDKRTALTYIESDGSWHRASKGAPEQIVALCNLREDAKKKVHAIIDKFAERGLRSLAVARQEVPERTKESAGGPWQFVGLLPLFDPPRHDSAETIRRALNLGVNVKMITGDQLAIAKETGRRLGMGTNMYPSASLLGQDKDASIAALPVEELIEKADGFAGVFPEHKYEIVKKLQERKHICGMTGDGVNDAPALKKADIGIAVADATDAARGASDIVLTEPGLSVIISAVLTSRAIFQRMKNYTIYAVSITIRIVFGFMFIALIWKFDFSPFMVLIIAILNDGTIMTIAKDRVKPSPMPDSWKLREIFATGIVLGGYLALMTVIFFWAMKETSFFSDKFGVRHIGESPHEMTAALYLQVSIVSQALIFVTRSRSWSYVERPGLMLVVAFAIAQLIATLIAVYANFGFARVKGIGWGWAGVIWIYSVVFYVPLDLLKFAIRYILSGKAWVNMLENKTAFTTKKDYGKEQREAQWALAQRTLHGLQPPETSGIFNDKNSYRELSEIAEQAKRRAEVARLRELHTLKGHVESVVKLKGLDIDTIQQHYTV
